# Coloration of nylon textiles with natural dyes: a critical review of recent progress, dye–fiber interactions, fastness, and functional performance

**DOI:** 10.1039/d6ra07080a

**Published:** 2026-09-25

**Authors:** Muksit Ahamed Chowdhury, Mohammad Mahbubul Alam, Nayeem Ahmed, Md. Shahinoor Islam, Badhon C. Mazumder

**Affiliations:** a Department of Textile Engineering, Ahsanullah University of Science and Technology Dhaka 1208 Bangladesh; b Department of Textile Engineering, Northern University Bangladesh Ashkona Dakshinkhan Dhaka 1230 Bangladesh badhoncmazumder@gmail.com; c Department of Chemical Engineering, Bangladesh University of Engineering and Technology Dhaka 1000 Bangladesh

## Abstract

Textile coloration is challenged by the use of petrochemical dyes, intensive auxiliary use, high water and energy consumption, and the production of complex effluents. Natural colorants offer renewable alternatives, but their environmental superiority cannot be assumed for durable synthetic fibers. Nylon requires dedicated evaluation because its amide backbone, terminal functional groups, semicrystalline morphology, and grade-dependent free volume make its dyeing behavior unlike that of other fibers. This critical review systematically searched Scopus and the Web of Science for English-language journal articles published during 2000–2026; screening and duplicate removal yielded 158 unique studies. The reviewed evidence supports chemical compatibility between nylon and several natural-colorant classes through ionic interactions, hydrogen bonding, dipole interactions, hydrophobic partitioning, coordination, and diffusion into amorphous regions. Acidic conditions commonly enhance uptake by protonating amino end groups, while crystallinity, free volume, temperature, pretreatment, and colorant aggregation control diffusion and fixation. Microwave, ultrasound, plasma, and enzymatic processing can improve extraction, mass transfer, surface accessibility, or mild fixation but are not inherently cleaner without quantified energy, chemical, and scale-up data. Adsorption and kinetic models are often statistically adequate yet mechanistically overinterpreted. Light fastness, extract reproducibility, wet durability, and retention of functional properties remain major weaknesses, while metallic mordants may conflict with cleaner-production objectives. Environmental inventories, toxicity, cost, and pilot-scale evidence remain substantially weaker than coloration data. By integrating nylon chemistry, processing, performance, sustainability, and industrial readiness within a structure–process–performance framework, this review identifies standardized extracts, repeated-use durability testing, environmental and economic assessment, and pilot validation as priorities for credible cleaner coloration.

## Abstract 2

Graphical abstract illustrating natural colorants for nylon/polyamide textile coloration, functional color development, cleaner processing, and scale-up toward sustainable textile applications. Image created using ChatGPT (Enterprise).

## Graphical abstract

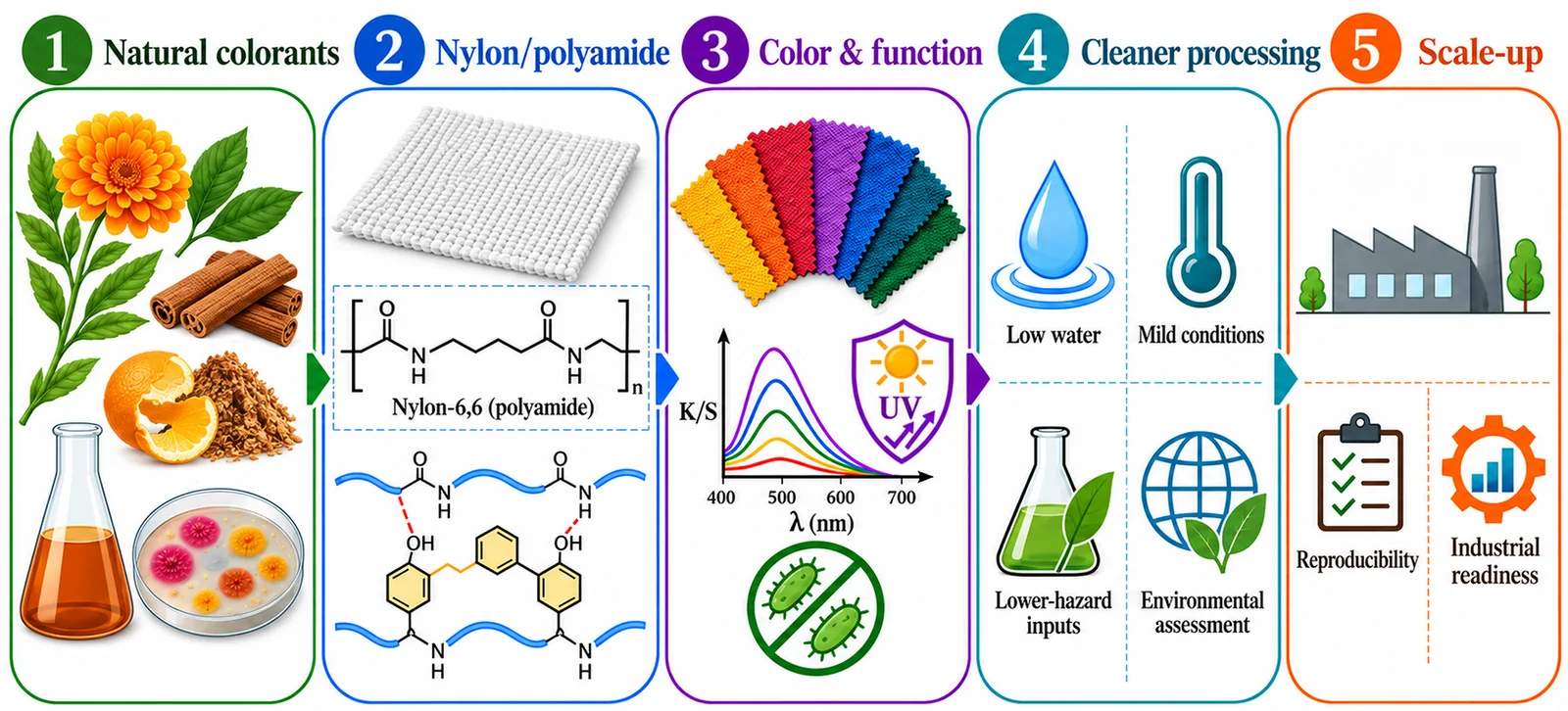

## 1 Introduction

Coloration is one of the oldest and most visible technologies used by humans to impart aesthetic value, cultural identity, functional meaning and marketability to textiles.^1,2^ The early materials used to make textile dyes were primarily derived from plants, insects, molluscs, minerals and other naturally occurring substances before industrial dye chemistry was developed.^3^ Indigo, madder, weld, cochineal, turmeric, annatto, saffron and other colorants were used not only in the dyeing of textiles but also in food, cosmetics, medicine, trade and cultural activities.^1,4,5^ However, the seasonal and regional fluctuation of natural dyes and the lack of uniformity in terms of shade and source were increasingly becoming problematic for large-scale textile production.^3,6,7^ These challenges were overcome by the discovery and industrial production of synthetic dyes in the nineteenth century, offering improved brightness, a broader color range, reproducibility, ease of use, and more reliable supplies.^8,9^ Concurrently, synthetic coloring has also led to greater reliance on petrochemical feedstocks, process auxiliaries, and water-intensive processes and resulted in effluents of high chemical complexity.^6,10–12^ The use of natural dyes should thus be considered again for coloring systems based on renewable raw materials and low-toxicity chemistry while offering faster processing and maintaining the functional properties of textiles.^13^

The reassessment is particularly relevant for synthetic fibers, which are used widely in apparel and technical textiles because of their durability, dimensional stability, resistance to abrasion, ease of care and processability.^14–16^ Nylon/polyamide is one such fiber because it possesses high tensile strength, elasticity and resilience, dyeability, and thus is used in hosiery, sportswear, carpets, apparel blends, and technical products.^17,18^ Nylon is distinct from other hydrophobic polyesters because it has amide linkages in its polymer backbone and terminal amino and carboxyl groups. These functional groups can interact with colorants through protonated amino groups, hydrogen bonding, ionic attraction, coordination, hydrophobic interactions, and diffusion into the amorphous regions.^13,19–22^ However, the semicrystalline structure of nylon can hinder colorant diffusion during dyeing and may require higher processing temperatures, auxiliaries, and longer processing times, making the process more complicated and increasing the environmental impact of dyeing.^23–25^

The conventional coloration of nylon generally uses a dye system such as synthetic acid, disperse, neutral, metal-complex, or reactive dyes, which may be supplemented with acids, salts, levelling agents, fixing chemicals, high-temperature conditions, and multiple wash cycles.^26–28^ These processes can offer good, consistent colors but also have raised issues with chemical use, colored wastewater, auxiliary load, and post-treatment needs.^29–31^ In response, natural and bio-based colorants have been renewed as potential alternatives for nylon coloration. Research in recent times has focused on plant-derived extracts, agricultural and industrial waste-derived colorants, animal-derived colorants, microbial and fungal pigments and bioengineered indigoids and enzymatically generated phenolic pigments used on nylon/polyamide substrates.^13,20,22,32–44^ In addition to color, a few systems have been reported to provide ultraviolet protection, antibacterial or antioxidant properties, fluorescence, pH responsiveness, or enhanced thermal properties.^20,35,39,40,42,44–47^ However, environmental superiority does not come automatically from being natural.^48–50^ A natural-dyeing process for nylon can be environmentally weak if it relies on toxic metallic mordants, high liquor ratios, poorly defined extracts, solvent-based pigment recovery, poor fastness, and repeated redyeing. Therefore, the cleanliness of using natural coloration needs to be evaluated in terms of the integrated process-performance trade-off and not only the source.^51^

**Fig. 1:**
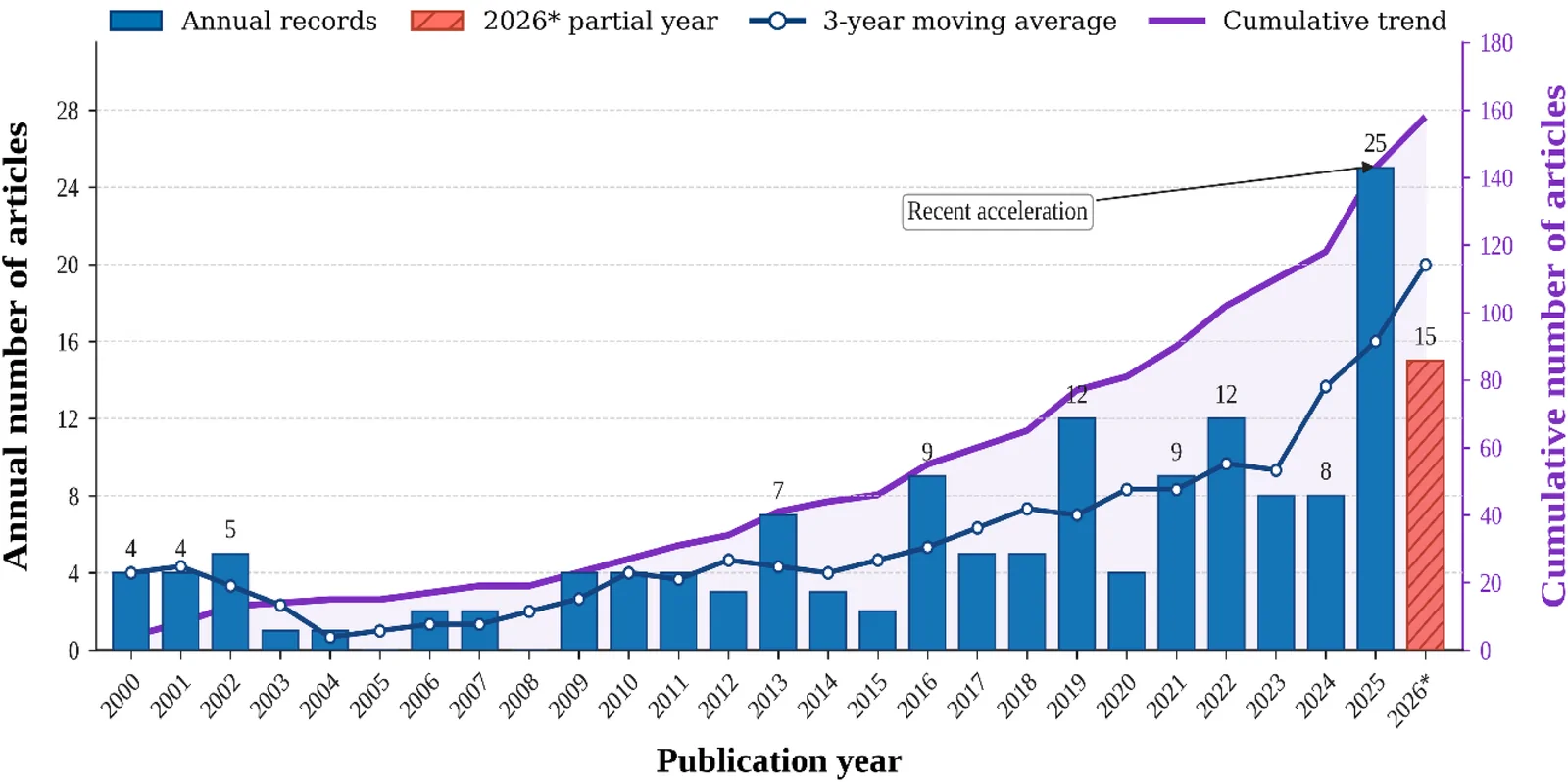
Publication growth in the reviewed literature on natural coloration of nylon/polyamide textiles from 2000 to 2026, showing annual output, the three-year moving average, and cumulative article count. Data for 2026 cover publications indexed through 30 June.

The publication record compiled for this review is presented in Fig. 1 and reveals a steady increase in publications after 2020. This growth suggests that the natural coloration of nylon/polyamide is no longer limited to scattered experiments but is now a growing research direction in the fields of cleaner production, natural products chemistry, polymer science, functional textiles, and sustainable dyes. The evidence base included 158 unique articles that were identified *via* a screening process in Scopus and Web of Science, thereby ensuring a wide and contemporary evidence base for the critical synthesis. The growing volume of studies requires not only summarization but also a structured approach to evidence appraisal, since there is significant variation among the reported results across dyestuffs, nylon grades and constructions, mordanting routes, pH and temperature conditions, liquor ratios, dye fastness testing, by functional-durability testing, and sustainability verification.

Though a number of reviews have been published on natural dyes and natural dyeing methods for textiles,^7,52,53^ to the best of our knowledge, there is no specific critical review that has summarized and analyzed the natural dyeing of nylon/polyamide textiles in relation to dye–fiber interactions, fastness, functional durability, cleaner production, and industrial readiness. Previous reviews tend to compare natural dyes on cotton, wool, silk, or a combination of these fibers, and synthetic dyes are either included in the latter part of the review or discussed only secondarily when discussing nylon as a secondary fiber.^54–56^ Thus, several specific questions concerning nylon that remain unanswered: which classes of natural dyes are best suited to polyamide chemistry; how the grade and textile form of nylon affect dye uptake; when acidic protonation is advantageous or disadvantageous; whether biomordants can compete with metallic salts; how the dyeing process, including microwave, ultrasound, plasma, enzymatic and low-water processes, influences coloration and durability; and whether reported functional properties hold after laundering and use. Therefore, a dedicated synthesis process focused on nylon is required as the results and guidelines from the studies on cotton, wool, silk or polyester synthesis cannot be directly applied to polyamide synthesis.

This review focuses on these gaps by critically analyzing the sources of natural dyes, extraction and preparation methods, mordanted and mordant-free dyeing systems, process-intensification measures, mechanisms of dye–fiber interaction, adsorption and kinetic evidence, color strength, fastness, functional performance, cleaner-production claims, and industrial readiness. Instead of discussing each of the numerous studies individually, it discusses points of agreement, points of disagreement, the reasons for these disagreements, and the systems that appear to be technically and environmentally feasible for nylon textile products. The aim of the review is to collate the many publications on this topic in a clear and orderly fashion, to help researchers identify classes of dyes that have potential for future use on nylon, guide textile chemists in creating better and safer dye–fiber interactions, guide sustainability researchers to differentiate between verified cleaner production and unsupported green claims and to understand which forms of natural coloration are viable for future use with polyamide, and help industry better evaluate which natural coloration systems are feasible for use in nylon.

## 2 Systematic literature search and review methodology

A systematic literature search was carried out to identify specific studies that directly dealt with the use of natural dyes for the production of cleaner coloration of nylon/polyamide textiles. The two academic databases selected were Scopus and Web of Science Core Collection, because they have a broad and well-established scope of peer-reviewed publications in textile science, materials chemistry, sustainable processing and functional materials. The search strategy was designed to identify studies involving natural dyes, natural pigments, bio-based colorants and the application of such colorants to a nylon or polyamide textile substrate.

The search in Scopus was conducted using the article title, abstract, and keywords fields with the keywords “Natural dye” AND “Nylon OR Polyamide”. This initial search resulted in 413 records. The final results were then narrowed by excluding non-relevant types of documents, such as notes, errata, books, conference reviews, book chapters, and retracted documents. Journal articles in English, published between 2000 and 2026, were retained. These filters resulted in 294 Scopus records, which were sent for further screening.

The same search was replicated in the Web of Science Core Collection using the Topic field with the Boolean search expression “Natural dye” AND “Nylon OR Polyamide”. The initial search produced 270 records. The results were further filtered by excluding book chapters and conference abstracts, and excluding publications in non-English languages, and restricting the publication year to 2000–2026. After refinement, 249 Web of Science records were exported.

Particular attention was given to the publication status of recent 2025–2026 records. Such studies were retained only when a formally published and indexed record was available in Scopus and/or Web of Science and the bibliographic information could be verified through a stable DOI or an official publisher record. Preprints, unpublished manuscripts, and other provisional records without a finalized published version were not included. For articles published online ahead of their assigned issue date, eligibility was based on the availability of the final published version rather than the later issue date. Open-access status was not used as an inclusion criterion.

The exported records from both databases were screened based on their titles and abstracts. A study was considered eligible when: (i) nylon/polyamide or a nylon-containing textile was used as an experimental substrate; (ii) a natural, bio-derived, plant-derived, animal-derived, microbial, waste-derived, or naturally derived colorant was applied to the substrate for dyeing, coloration, mordanting, biomordanting, or related functional coloration; and (iii) at least one outcome relevant to this review was reported, including dyeing optimization, color strength (*K*/*S*), CIELAB values, fastness properties, dye–fiber interactions, adsorption behavior, functional properties, or cleaner-processing performance. Studies involving multiple textile fibers were retained only when nylon/polyamide-specific experimental results were reported. Papers that dealt solely with synthetic dyes, natural dyeing of textile fibers other than nylon/polyamide, non-textile applications of natural pigments, general production methods of natural pigments, or natural pigment treatment of effluents unrelated to the coloration of nylon/polyamide were excluded. Reviews and other non-original publications, conference abstracts without sufficient experimental information, and records lacking primary experimental evidence relevant to the review scope were also excluded.

**Fig. 2:**
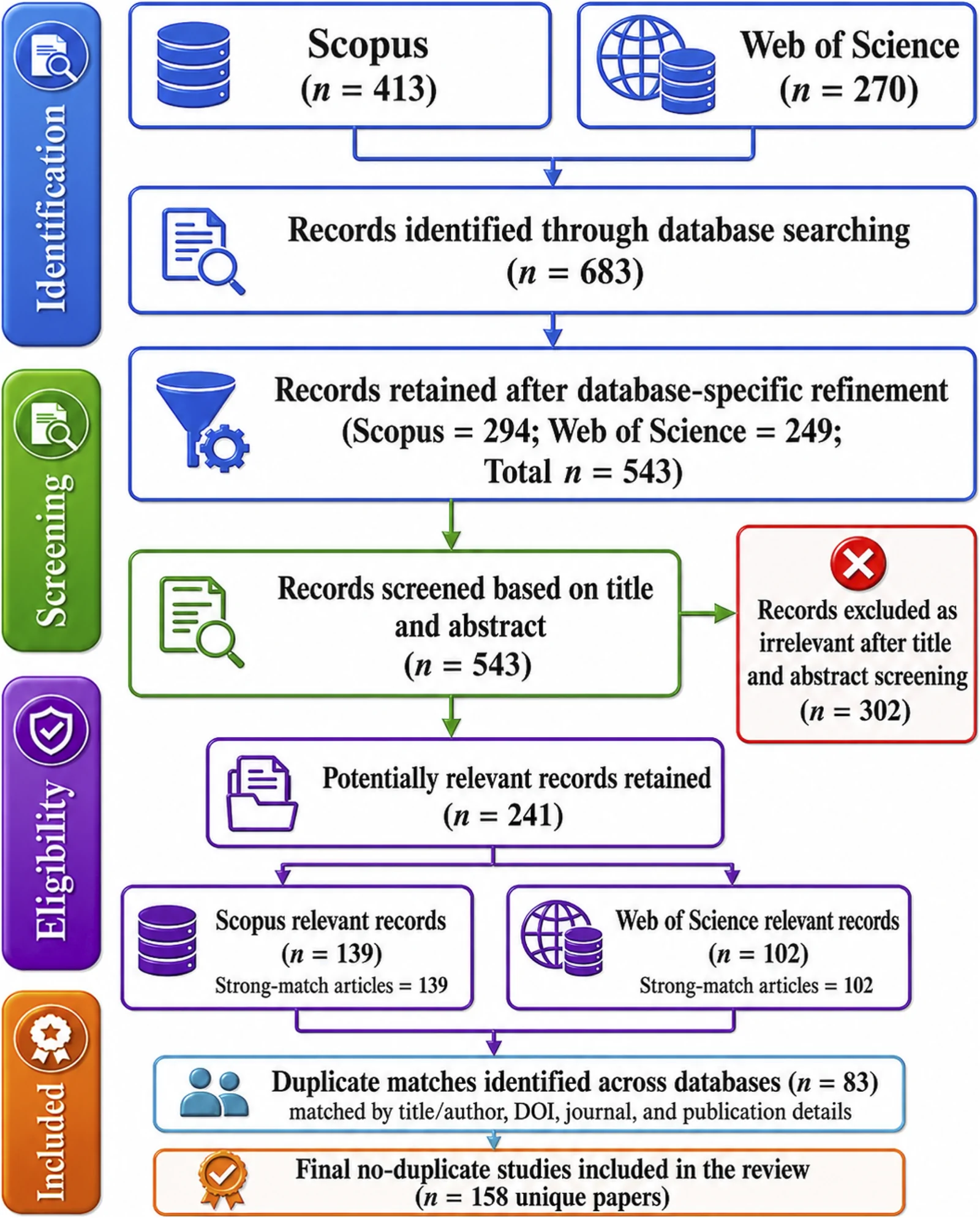
Systematic literature search, screening, duplicate removal, and final selection workflow for studies on the natural dyeing of nylon/polyamide textiles.

Following the title and abstract screening, 139 Scopus records and 102 Web of Science records were found to be good matches with the review scope, while 302 records were excluded as being outside the review scope. The relevant records from both databases were then merged, resulting in 241 strong-match records prior to duplicate removal. Duplicate articles were detected using comparisons of titles, authors, journal information, publication year, DOI, *etc.* A final set of 158 unique studies was identified as the core literature base for this review after the removal of 83 duplicate records. The overall search, screening, duplicate removal, and final article selection process is summarized in Fig. 2.

Selected articles were then compiled thematically for a critical rather than a purely descriptive review. The major topics covered were the characteristics of nylon/polyamide fibers, natural dye sources and their chemical classes, dye extraction and preparation, mordanting and biomordanting systems, dyeing optimization, dye–fiber interaction mechanisms, color strength, fastness, functional properties, cleaner processing strategies, sustainability, and future research gaps. Nominal dye concentration was not interpreted as directly comparable between studies, as both purified colourants as well as chemically heterogeneous crude colourants were used in the reviewed studies. Thus, the level of characterization of the colorants, as well as the dosage basis, nylon substrate, extraction methods, dyeing methods and reported performance parameters were taken into account in cross-study interpretation. Comparisons were made, where possible, with results provided by marker compounds, pigment purity or chromatographic/spectroscopic characterization. Poorly characterized extracts, typically reported as biomass mass, extract volume, % owf or nominal concentration, were interpreted as mostly within individual studies or as evidence of directionality, not as a common concentration of active-colorants.

There was no formal numerical evidence-quality or risk-of-bias tool used due to the heterogeneity of studies included. Rather, the evidence was judged critically on the basis of the completeness of reporting and the ability to reproduce the work, including experimental controls, replication and statistical analysis, process descriptions, and use of recognized textile testing methods. Some studies were not replicated or statistically supported but were retained for the analysis; however, their results were discussed with greater caution when comparing them across studies. This methodological approach allowed the review to focus only on the scientific and technological advances of the natural dyeing of nylon/polyamide textiles and maintained a clear focus on the correlation among the coloration of fabrics, their functional properties and sustainable textile processing.

## 3 Nylon/polyamide as a substrate for natural dyeing

Nylon needs a separate discussion because it is neither an inert hydrophobic synthetic fiber nor a protein fiber. Its amide backbone and chain-end groups can bind ionizable natural dyes, but its compact, semicrystalline and partly hydrophobic morphology limits dye accessibility. Thus, useful acidic dyeing is frequently reported, yet color depth, light fastness and functional durability may remain below those observed for protein-rich substrates.

### 3.1 Chemical structure and dye-interactive groups

The dye-interactive chemistry of nylon is based on repeating amide linkages, –CONH–. Carbonyl oxygen atoms accept hydrogen bonds and act as dipolar sites, while N–H groups add local polarity. These sites explain the affinity of nylon for phenolic acids, flavonoids, curcuminoids, anthraquinones and other oxygen-rich colorants.^19,20,22,57,58^ However, amide groups buried in crystalline regions are less available; therefore, dyeability depends on chemical compatibility and access to amorphous or near-surface regions.

Terminal amino and carboxyl groups are fewer than the backbone amide groups but are decisive dye sites because they control fiber charge. In acidic baths, –NH_2_ is protonated to –NH_3_^+^, creating cationic sites for anionic carboxylated, sulfonated or phenolate-type dyes, as reported for tannin-, flavonoid-, cochineal-, lac-, baicalin-, coffee- and bark-based systems.^59–64^ In alkaline media, –COOH can become –COO^−^, which usually repels anionic dyes but can favor cationic berberine.^65,66^ In silk/polyamide systems, isoelectric behavior around pH 3.5–4.5 marks the shift from positively charged amino sites to carboxylate-controlled surfaces, so dye adsorption can change sharply across this range.^36^

Hydrophobic methylene segments also contribute by associating with aromatic and conjugated dye skeletons, including curcumin, alizarin, indigoids and anthraquinones.^39,67–69^ Nylon natural dyeing is therefore best interpreted as a combined ionic, hydrogen-bonding, coordination and hydrophobic process rather than a single salt-link mechanism.

### 3.2 Differences among nylon grades and textile forms

The nylon grade influences dyeability. The two polymers, PA6 and PA66, possess amide linkages and terminal groups, but PA66 is in general more compact and more crystalline, while PA6 is more likely to provide easier diffusion and a lower glass-transition barrier.^21,41,58^ Many papers only report “nylon” or “polyamide”, so a comparison across studies is not always possible.

Comparative evidence indicates that structure can be more important than amino end-group content. PA56 showed the best dyeability because it had the lowest crystallinity, 32.46%, and the highest fractional free volume, 9.3%, compared with PA6 at 36.02% and 8.6% and PA66 at 39.03% and 8.4%, respectively.^70^ Therefore, amorphous accessibility becomes an important factor for moderate-sized natural dyes. Alternatively, nylon 5,10 contains longer methylene segments, which reduce the density of amides and hydrogen bonding, and has reduced stability at 90–100 °C, making it more appropriate for enzymatic coloration at 55–60 °C.^33^ PA4,10 is also an addition to the bio-based substrate group, as a fabric made from Ecopaxx Q130E PA4,10 fibers has been colored with 1 wt% alizarin or alizarin-ZnO colorants during melt spinning; therefore, coloration *via* a fiber formation route is different from the coloration process of exhaust-dyed fabrics.^71^ The form of textiles also affects their performance. The *K*/*S* value of recycled PA66 dyed with weld was higher than that of cotton, 1.8 *versus* 1.2, but its b* value was much lower, 13.3 *versus* 28.5, indicating that *K*/*S* is not a reliable indicator for shade quality.^35^ Polyamide/elastane blends cannot be considered pure nylon because elastane has an impact on swelling, geometry, and levelness.^57,72–75^ The pH-controlled microbial dyestuffs showed balanced pigment distribution of 51.0% and 49.0% in silk/PA66 at pH 3.^36^ The grade, the recycling ratio, the blend ratio, the construction process, and the pretreatment should therefore be reported.

Fabric construction should also be considered separately from polymer grade when comparing dyeing and functional performance. Areal density, thickness, weave or knit structure, yarn specification, and thread or stitch density can influence the comparability of color strength and functional results. In the PA56, PA6, and PA66 comparison,^70^ the fabrics were produced with identical construction, allowing differences in dyeability to be associated more confidently with polyamide structure. In contrast, the industrial study^76^ used a compact interlock polyamide fabric with an areal density of 300 g m^−2^, in which the undyed fabric already showed extremely high UV protection. Future studies should therefore report detailed fabric specifications and, where possible, use construction-matched substrates when comparing different nylon grades or coloration routes.

### 3.3 pH-dependent charge and natural-dye affinity

Most nylon natural-dye systems work best in acidic to mildly acidic baths, usually at pH 2–5, because protonated amino end groups attract anionic dyes. Babool bark showed this clearly: *K*/*S* was 7.3 at pH 2, 4.9 at pH 3, 5.7 at pH 4 and 6.0 at pH 5, then dropped to 1.8 at pH 11 and 0.62 at pH 13; pH 5 was preferred to avoid possible acid hydrolysis.^59^ Similar behavior was reported for coffee pomace, cochineal, sesame, baicalin, curcumin and olive vegetable water.^43,60,61,63,77^ Curcumin exhaustion at pH 4 and 60 °C rose only from about 98.1% after 1 h to 99.0% after 6 h.^39^

The optimum pH still depends on the dye class. Tannins and flavonoids benefit from –NH_3_^+^ sites through ionic attraction and hydrogen bonding. Anthraquinones such as cochineal, lac, alizarin, emodin and dermocybin also use carbonyl/hydroxyl groups and metal coordination in mordanted systems.^61,62,78,79^ Indigoids are an exception because alkaline leuco formation precedes oxidation.^41,70,80^ Cationic berberine is another exception because alkaline carboxylate sites can enhance electrostatic attraction.^65,66^ Thus, pH should be selected according to dye charge, dye stability, nylon grade and fiber-damage risk, not a universal acidic rule.

### 3.4 Crystallinity, swelling, and diffusion behavior

Nylon dyeing is typically characterized by fast surface adsorption followed by slower diffusion to the amorphous regions. The crystalline domains block penetration while the amorphous domains and free volume between the chains facilitate internal fixation. Poor dye diffusion can therefore result in high surface color but limited washing, rubbing, or light fastness. This is also confirmed by the PA56 comparison; its lower crystallinity and higher free volume facilitated dye uptake more readily than in PA6 and PA66.^70^ Compact nylon does not have as many accessible dye sites as wool, with one study on madder reporting 0.036 equiv. kg^−1^ of primary amine sites in nylon compared to 0.82 equiv. kg^−1^ in wool.^81^

Increasing the temperature generally enhances chain mobility and swelling, particularly above the nylon Tg near 50 °C. For example, mangrove bark dyeing improved from *K*/*S* of 1.66 at 30 °C to 5.38 at 90 °C; for olive vegetable water, dyeing improved from 50 °C to 100 °C.^40,43^ However, a higher temperature does not necessarily lead to a cleaner process, as dyes can be degraded, shades can become dull, and nylon 5,10 can be favored to handle mild enzymatic conditions.^33^ Pretreatments should expand the number of accessible sites without causing damage. Rice-straw and indigo dyeing were improved by plasma treatment without damaging the backbone;^20,41^ ultrasonic dyeing was effective at 30 °C for 30 min;^82^ and microwave and laser treatments improved surface accessibility.^45,83,84^ Moisture-assisted plasticization can further open amorphous microgaps, allowing nylon to interact more readily with natural dyes compared to more hydrophobic polyester when heated in a similar manner.^41^

### 3.5 Nylon-specific cleaner-coloration framework

Five connected dimensions were used in this review to compare conventional exhaust/mordanting, biomordanting, and advanced processing routes such as microwave, ultrasound, plasma, laser, and electron-beam treatments: coloration performance, durability and functionality, chemical safety, resource efficiency, and industrial readiness. The comparison considered reported *K*/*S*, CIELAB/Δ*E*, exhaustion and levelness; fastness and functional retention; temperature, time, liquor ratio and reported power or energy; mordant, auxiliary and solvent burdens and available effluent indicators; and fiber integrity, reproducibility and scale-up compatibility. Direct numerical comparisons were emphasized where conventional controls or sufficiently matched conditions were available; otherwise, the routes were interpreted qualitatively rather than ranked solely by *K*/*S* or treatment time.

Baicalin-dyed PA6 gave washing/rubbing grades of 3–4 to 4–5,^63^ and madder-dyed PA56 gave wash/rub grades of 4–5, although light fastness was only 2–3 and improved to 3–4 after aloe vera post-treatment.^70^ The light fastness of weld-dyed recycled nylon was the lowest, at just 5, after finishing with sodium alginate/TiO_2_,^35^ while red-sandalwood-dyed nylon was still at around 1–2.^85^ Functional values are promising, but they need durability tests. The UPF of rice straw was raised from 2.3 to 72.6 and 79.9 after plasma^20^ and the UPF of madder was raised from 5.3 to 17.4–18.4.^86^ The exhaustion of dyed nylon 6 was raised from 77.38% to 99.79%, while antibacterial reductions against *Escherichia coli* and *Staphylococcus aureus* were raised to 97.80% and 99.33%, respectively, by green AgNP-assisted dyeing.^87^

**Fig. 3:**
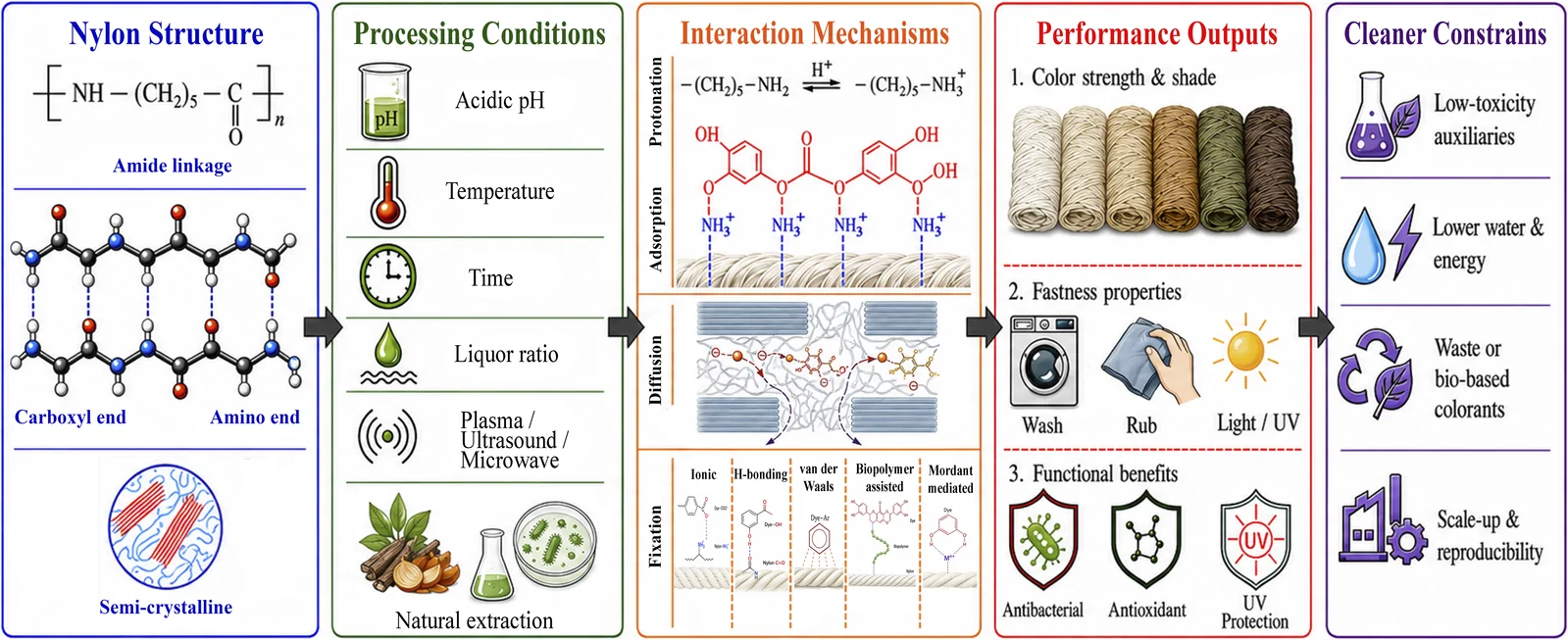
Nylon-specific structure-process-performance framework for natural coloration.

Chemical safety is essential because natural dyeing loses its cleaner advantage when chromium, copper, tin, zinc, or other high-concern mordants dominate.^22,37,78^ Bio-mordants such as tannic acid, chitosan, and aloe vera, as well as sodium alginate and justified low-dose inorganic finishes, are preferable if effluent load and durability are reported.^19,35,42,70^ Resource efficiency should include extraction yield, liquor ratio, temperature, time, bath reuse and wastewater quality. Waste-derived dyes such as rice straw, coffee grounds, peanut skin, pomegranate peel, olive vegetable water and faba bean husk strengthen sustainability only when extraction and dyeing are not chemically intensive.^20,37,43,45,74^ Industrial readiness requires exact substrate identification, reproducible dye composition, scalable pretreatment, as well as wash, rubbing, light and perspiration fastness, especially for recycled nylon and polyamide/elastane blends. These interdependent elements are integrated into the nylon-specific structure–process–performance framework shown in Fig. 3.

The strongest near-term route is substrate-matched coloration: mildly acidic dyeing, often pH 4–5, for anionic phenolic, flavonoid, tannin, and many anthraquinone dyes; sufficient temperature, often 80–90 °C for PA6/PA66, for amorphous-region diffusion; and low-toxicity stabilization when light fastness or functionality is needed. PA56 is promising because its low crystallinity and high free volume support diffusion, whereas bio-based nylon 5,10 needs mild processing.^33,70^ Exceptions need separate logic: indigoids require redox-controlled leuco dyeing, and cationic berberine can favor alkaline carboxylate sites.^65,66,80^ Strong systems identify the polyamide grade, match pH and temperature to the dye chemistry, avoid hazardous mordants and demonstrate shade, fastness and functional durability together.

## 4 Natural colorant landscape and preparation routes

**Fig. 4:**
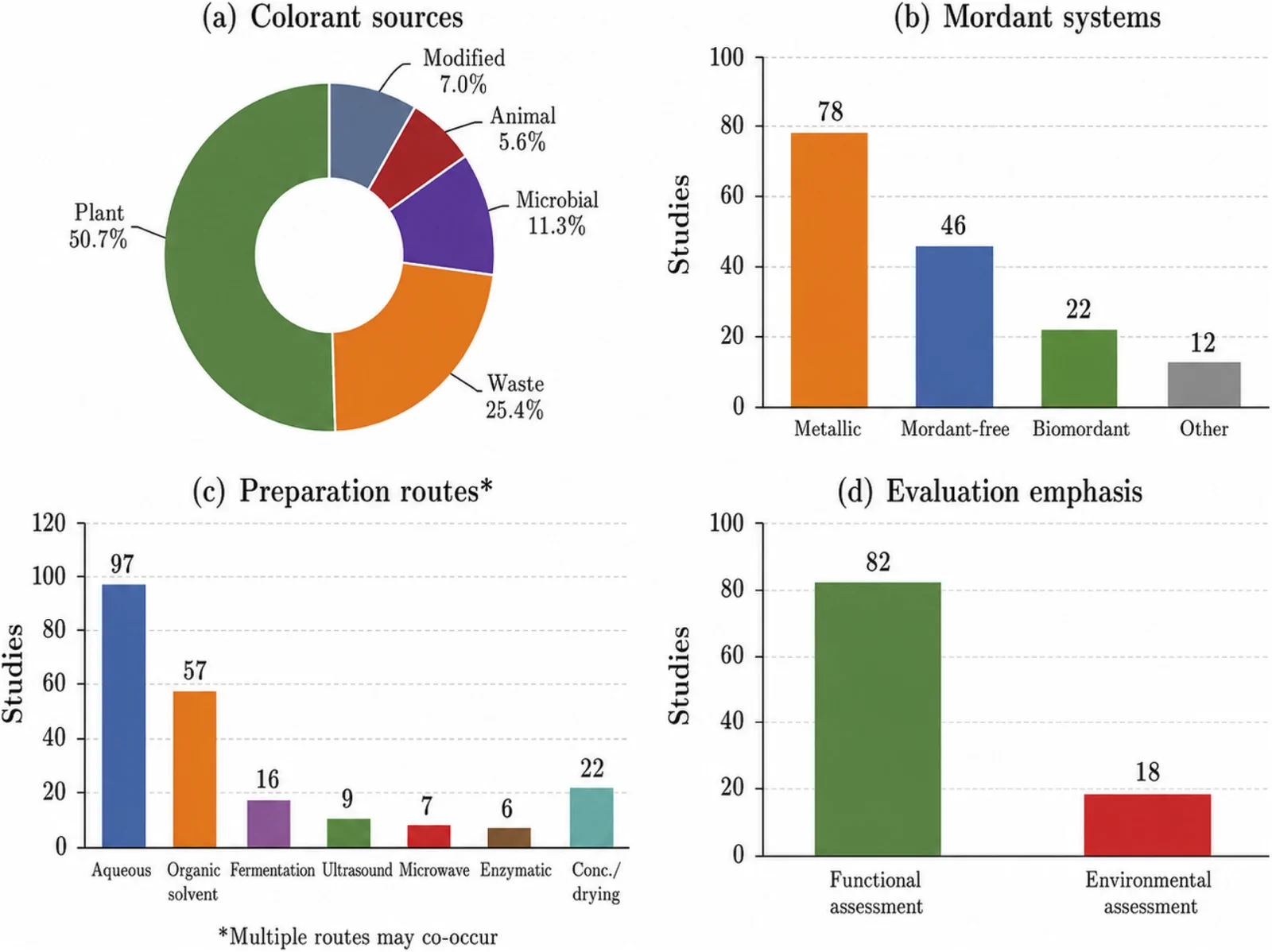
Distribution of (a) colorant sources, (b) mordanting systems, (c) preparation routes, and (d) evaluation emphasis in natural-colorant studies on nylon/polyamide.

The botanical colorants are the predominant evidence base, followed by agricultural and industrial residues, microbial pigments, animal-derived dyes and modified naturally derived formulations. The most common substrates studied were PA6 and PA66, while PA5,6, PA5,10, recycled nylon and nylon blends were studied to a lesser extent. Blended, interwoven, and multicomponent constructions were also significantly outnumbered by pure nylon constructions, which were therefore used as the basis for direct translation in many commercial textiles. The most important preparation–application sequence continued with aqueous extraction followed by exhaust dyeing. Organic solvents were commonly used for poorly water-soluble curcuminoids, anthraquinones, carotenoids, and microbial pigments. Microwave irradiation, ultrasound, enzymatic conversion, fermentation, ultrafiltration, concentration, and freeze-drying formed smaller but technically important process groups. However, the investigation of functional performance was far more common than that of extraction yield, energy consumption, solvent recovery, residual-biomass utilization, and effluent quality. Thus, the literature shows great diversity in sources and technologies but less evidence for standardized colorant production and industrial scalability. Fig. 4 shows the overall distribution of the sources of color, mordanting systems, preparation methods and evaluation emphasis.

### 4.1 Plant-derived colorants

Plant-derived systems were the largest source category, which included crude extracts, commercial powder, and purified compounds. These forms are not directly comparable, since they contain significantly different concentrations of active colorants, and their chemical definition, and preparation burdens differ substantially.

Leaf-derived sources included black tea (*Camellia sinensis*),^88^ neem (*Azadirachta indica*),^89,90^ sea buckthorn (*Hippophae rhamnoides*),^72^*Eucalyptus grandis*,^70,91^*Aegle marmelos*,^92^ mulberry,^76^*Cassia obovata/Senna italica*,^84^ teak (*Tectona grandis*),^93^ walnut (*Juglans regia*) leaves and stem–leaf mixtures,^94,95^ henna and *Pterocarya fraxinifolia* leaves,^22^ spinach,^70^ naturally fallen *Terminalia catappa* leaves,^96^ and purified luteolin.^97^ These sources are typically rich in tannins, flavonoids, phenolic acids or chlorophyll-related compounds, but most studies reported the biomass mass instead of the concentration of the marker compounds. The variation in nylon *K*/*S* could therefore be as much due to differences in chromophore loading as to differences in dye–fiber affinity.

Bark, wood, stem, and resin sources comprised Brazilwood (*Paubrasilia echinata*),^98^ babool from *Acacia nilotica* and *A. arabica*,^59,64^*Berlinia grandiflora*,^99^*Casuarina cunninghamiana*,^100^ red sandalwood (*Pterocarpus santalinus*),^69,85^ water opepe (*Nauclea diderrichii*) and water ekimi (*Vanilla planifolia*, as reported),^101^ catechu (*Acacia catechu*),^70,76^ walnut stems,^94,95^ and Dragon's blood resin.^102^ The materials yielded beige, brown, orange-red, violet, and dark-red colors; however, destructive harvesting of bark or heartwood, slow regeneration of these materials, and reliance on mordants diminish the of these sources sustainability unless they are sustainably managed or residual forestry streams are used.

Roots, rhizomes, and purified plant compounds included turmeric and curcumin,^39,42,46,67,70,103–109^ Japanese knotweed-derived emodin,^110^ madder and its anthraquinones alizarin, purpurin, and nordamncanthal,^68,70,73,76,81,86,111–113^ rhubarb,^70,76^*Berberis vulgaris* root,^65^ ratanjot (*Arnebia nobilis*),^114^ and *Scutellaria*-derived baicalin or *Radix Scutellariae*.^47,63,70^ Commercial berberine chloride was also tested, but there was no traceability to a specific plant batch used in the experiments.^66^ Purification enhanced the reproducibility of the dose and the mechanistic interpretation; however, it added additional burdens of medicinal competition, destructive root collection, and solvent- or chromatography-based isolation.

Flower-, petal-, and stigma-derived sources included weld (*Reseda luteola*),^35,115,116^*Papaver rhoeas*,^117^ mullein (*Verbascum thapsus*),^118^ saffron stigma or commercial saffron,^105,119^ saffron and safflower petals,^13^ rhubarb flower (*Rheum ribes*),^120^ marigold,^76^ and *Calendula officinalis* florets.^100^ Their varying responses to yellow, orange, pink, red, and brown indicate that this is not a consequence of the plant part alone, but rather of the dominant chemistry of the chromophore, its extraction selectivity, and the fixation.

Fruit-, seed-, pod-, nut-, and whole-plant sources included annatto/achiote (*Bixa orellana*) seeds or potassium norbixinate,^57,83,91,121^ hawthorn (*Crataegus elbursensis*) fruit,^122^*Terminalia arjuna* and *Thespesia populnea* fruits,^123^ pomegranate arils and seeds,^124,125^ black currant,^82^ myrobalan (*Terminalia chebula*),^76,126^ harmal (*Peganum harmala*) seeds,^127^ mahogany (*Swietenia mahagoni*) pods,^38^ sesame seed,^77^ areca/betel nut (*Areca catechu*),^128,129^ beetroot (*Beta vulgaris*),^130^ botanical indigo and indigo–turmeric combinations,^41,103^ and *Prangos ferulacea* powder.^131^ Multi-source screens were also performed on spinach, rhubarb, catechu, madder, turmeric, *Radix Scutellariae*, mulberry, myrobalan and marigold.^70,76^

Overall, botanical diversity is a sign of the technical dyeability of nylon but not the maturity of the supply chain. There is still a lack of answers to questions regarding source authentication, marker-compound analysis, extraction yield, seasonal variations, competing uses, and batch-to-batch shade reproducibility.

### 4.2 Waste- and by-product-derived colorants

The circularity rationale of waste-derived plants is more convincing than purpose-collected plants when they displace an existing disposal burden. The agricultural and pruning residues were rice-straw pulping liquor,^20^ olive leaves,^70,132^ waste bamboo leaves,^133^ discarded avocado leaves,^134^ kapok leaves,^135^*Dacryodes edulis* leaves,^136^ saffron stamens,^115^ date-palm fibrillium,^137^ sweet-potato leaves^138^ and teak leaves used for pruning or timber harvesting.^139^ Their utilization may help to diminish the demand for dedicated crops, but collection, cleaning, drying, transport and seasonal storage are still required.

Food and agro-industrial residues comprised spent coffee grounds,^37,140,141^ coffee pomace,^60^ roasted peanut skin,^37^ banana peel,^75,142^ faba-bean husk,^74,142^ almond shell and delignified almond stem,^142,143^ onion skin,^70,142^ chestnut shell,^70^ walnut shell, rind, or green bark,^58,82,142,144,145^ tomato skins, seeds, and peels,^146^ artichoke, rosemary, and olive-oil prina,^142^ pomegranate peel or rind,^22,45,70,76,116,126,142,144,147^ and naturally fallen *Cassia fistula* pods.^148^ These streams can be concentrated at processing facilities but can cause problems with filtration, stability, and shade consistency because of remaining oils, sugars, proteins, salts, pesticides, and microorganisms. There are also many that compete with food-ingredient, antioxidant, feed, composting or energy-recovery applications.

Forestry, textile, and liquid-process residues included sugarcane-bagasse lignin nanoparticles,^149^ mangrove bark from charcoal production,^40,150^*Nesogordonia papaverifera* sawmill bark,^151^ silkworm excrement,^152^ eucalyptus steaming liquor,^153^ Turkish red-pine bark,^154^ red-alder saw wood,^155^ Abura and Ghana Obeche bark residues,^156^ and olive vegetable water.^43^ These systems are particularly relevant when colorant recovery simultaneously lowers the pollutant load of an existing process stream.

The designation “waste-derived” does not, by itself, demonstrate circularity. A credible assessment requires information on feedstock availability and composition, competing or alternative uses, pretreatment requirements, extraction yield, post-extraction residue management, and effluent quality; however, these parameters were reported far less frequently than color strength and fastness.

### 4.3 Microbial and fermentation-derived pigments

Bacterial colorants included biosynthetic 6,6′-dibromoindigo from recombinant *Escherichia coli*,^34^ nano-prodigiosin from *Serratia marcescens*,^36^ bio-indigo from engineered *E. coli*,^80^ indigoidine from engineered *E. coli* cultivated with okara hydrolysate,^157^ crude-gel prodigiosin from non-pathogenic *Serratia plymuthica*,^158^ pigments from a rose-red *S. marcescens* isolate and isolates 1a, 1b, Rea, and Brf,^159^ blue/purple and red pigments from *Streptomyces* sp. NP2 and NP4,^160^ prodigiosin from mutant *S. marcescens* jx1-1,^161^ and violacein/deoxyviolacein from *Janthinobacterium lividum* S-9601.^162^

These systems generate blue, purple, red and pink colorants in a season-independent manner, with the environmental performance of these systems influenced by medium composition, sterilization, aeration, fermentation time, colorant location and downstream isolation. The amount of purification may be reduced when applying the broth or clarified-suspension broth, while the intracellular pigments may need centrifugation, washing, disruption and organic solvents. For example, the *Streptomyces* route involved six days of fermentation followed by acidified ethyl acetate extraction and methanol/water redissolution.^160^

Fungal sources included red and yellow *Monascus* pigments or *Monascus* rice powder,^163,164^ emodin, dermocybin, and dermorubin from *Cortinarius sanguineus*,^165^ pigments from *Chlorociboria aeruginosa*, *Scytalidium cuboideum*, and *S. ganodermophthorum*,^166^ purified emodin and dermocybin from *Dermocybe sanguinea*,^79,167^ and an extracellular yellow pigment from *Aspergillus turcosus* PX588553.^44^ Purified anthraquinones enhanced dosing and mechanistic interpretation, while crude broths hindered isolation but increased the uncertainty of the composition.

Microbial production is thus a response to botanical seasonality rather than necessarily a means of reducing resource use. Cleaner performance demands safe strains, high titer, low-impact media, short cultivation times, limited purification, and direct compatibility with nylon coloration.

### 4.4 Animal-derived and other natural colorants

Animal-derived colorants were dominated by cochineal/carmine, lac, and molluscan indigoids. Carmine Red from cochineal insects was applied directly or with a chitosan interlayer,^19,168^ while cochineal from *Dactylopius coccus* or the historically reported *Coccus cacti* source was examined in adsorption, kinetic, mordanting, and color-performance studies.^61,78,169^ Lac from *Kerria lacca* supplied laccaic acid colorants.^62,126,170^ Molluscan-derived purple colorants included indigo, 6-bromoindigo, and 6,6′-dibromoindigo associated with *Hexaplex trunculus*, *Bolinus brandaris*, and *Stramonita haemastoma*.^171^

These colorants provide high tinctorial strength and strong nylon affinity, but their scalability is restricted by cost, sensitization, ethical or cultural concerns, dependence on animal-derived resources, limited supply, and frequent metallic-mordant use.

### 4.5 Modified and hybrid natural-derived colorant systems

Several studies substantially transformed natural compounds before or during coloration. Horseradish peroxidase converted catechol and hydroquinone into polymeric quinonoid pigments,^32^ while catechol and gallic acid were polymerized with or without a chitosan template.^33^ Curcumin was formulated as PVP- or Poloxamer-stabilized nanoparticles;^172^*Scrophularia striata* flower/stem extract and *Verbascum thapsus* leaf extract generated silver nanoparticles;^173,174^ and alizarin was immobilized on ultrasmall ZnO particles.^71^ Berberine from *Coptis chinensis* was thermally modified,^175^ areca polyphenols were converted into three azo dyes,^176^ and beetroot betalain/polyphenol extract was coupled with diazotized *o*-aminotoluene or *o*-aminobenzoic acid.^177^

These approaches can improve solubility, fixation, color depth, photostability, or functionality, but their environmental profile also depends on polymers, metals, oxidants, surfactants, solvents, and coupling agents. They are therefore more accurately classified as natural-derived or bio-assisted technologies than as simple natural-dye applications.

White sugar served only as a reducing and capping agent for silver nanoparticles,^87^ whereas Acid Black 172 was the actual dye. This system should therefore be classified as a bio-assisted synthetic-dye process rather than natural coloration. Lignin nanoparticles, by contrast, retained a genuine waste-derived colorant origin and are consequently discussed with waste resources.^149^

### 4.6 Chemical classification of natural colorants

The chemical structure is a more reliable basis for interpreting the color of nylon than its biological origin alone. The most commonly mentioned constituents were tannins and polyphenols. Their hydroxyl, carbonyl, and carboxyl groups enable hydrogen bonding, pH-dependent electrostatic interactions and metal coordination properties, whereas their aromatic structure provides ultraviolet absorption and antioxidant activity.^22,37,43,59,60,64,96,143,147^ Nevertheless, their molecular masses are heterogeneous, and they are easily oxidized, thus making them difficult to standardize and often resulting in yellow–brown, gray, or dark coloration.

Flavonoids, such as luteolin, baicalin, and weld-related compounds, produced predominantly yellow-brown tones and provided UV protection.^13,35,47,63,91,97,122,131^ Their nylon affinity was a function of hydroxylation, glycosylation, ionization, and bath pH. The most intense red, orange, violet, and deep-brown shade families were created by anthraquinones and naphthoquinones. Their conjugated structures tend to form hydrophobic associations and molecular aggregation, while hydroxyl and carbonyl groups can form hydrogen bonds and metal complexation.^68,79,81,110–114,167^

Curcuminoids gave a brilliant yellow color and were found to be useful as antioxidants or antibacterial agents, but were poorly water-soluble, pH-sensitive, light-sensitive and oxidation-sensitive.^39,42,46,67,104,105,108,172^ Indigoids and related quinonoids yielded blue, green, purple, and brown hues, but they were largely found to be poorly soluble in water, requiring reduction, biosynthesis, or modification of the indigoid structure to make them soluble.^34,41,80,85,103,157,171^ Carotenoids produced yellow-to-orange coloration, but were prone to oxidation and photofading,^13,57,100,119,121,146^ while systems containing betalains and anthocyanins produced red–violet coloration and were pH-responsive, but suffered from relatively poor stability.^117,124,125,130,177^

The production of a wider range of color and functional properties was further extended by the addition of pyrrole pigments, lignin, melanoidins, alkaloids, chlorophyll-related compounds and chemically unresolved extracts. However, it is not appropriate to simply assign an extract to a chemical class based on its botanical source. Chromatographic or spectrometric analysis and subsequent normalization against marker molecules are essential before discrepancies in nylon affinity, fastness or bioactivity can be confidently assigned to source chemistry. The general term “plant dye” is therefore unsatisfactory for comparative analysis because extracts from related plants that are chemically distinct can exhibit different behavior in nylon, and structurally similar chromophores from various biological sources can exhibit a similar affinity for nylon.

### 4.7 Extraction and colorant-preparation routes

The reported ranges represent collective operating windows across chemically different sources and should not be interpreted as directly comparable optimal conditions.

#### 4.7.1 Aqueous and pH-Assisted extraction

Aqueous extraction predominated due to the fact that water is cheap, comparatively safe, and directly compatible with exhaust dyeing. The extraction temperatures were reported as ranging from room temperature to near 100 °C, with most extraction methods involving processing at 70–100 °C for 30–120 min, though processing times ranged from a few minutes to 3 h. Soaking or maceration generally lasted 12–48 h and, exceptionally, approximately one month.^22,38,60,64,70,72,77,81,90,96,99,100,116,124,125,147,155,177^

Solid-to-liquid ratios ranged from approximately 1 : 2.5 to 1 : 40, with 1 : 10–1 : 25 frequently used.^22,38,58,70,78,81,84,96,99,124,144,147,148,150,155,177^ The biomass concentrations also varied significantly, from approximately 5 g L^−1^ up to several hundred g L^−1^; for instance, the extraction of sesame was investigated within the range of 5 to 500 g L^−1^.^77^ This variation makes cross-study analysis difficult because the concentration of the active chromophore is not measured.

The extraction pH was seldom reported, but the explored pH range was from around pH 2 to 11.^60,96,139^ The alkaline teak-leaf extraction gave a gravimetric yield of 40%, whereas pH 5 and 7 gave 31% and 28%, respectively, but it did not yield the maximum unmordanted nylon color strength.^139^ Similarly, pH 11 generated the most effective *Terminalia catappa* extract for subsequent acidic dyeing.^96^ These results demonstrate that higher extraction yield does not necessarily correspond to a higher *K*/*S* value because the extracted solids may contain significant amounts of non-chromophoric material.

Acidic or alkaline media have the potential to increase the release of ionizable phenolics, tannins, betalains and related compounds but may also lead to hydrolysis of glycosides, modification of sensitive chromophores and the formation of neutralization salts. Thus, their performance should be evaluated by the amount of chemicals consumed, the stability of the chromophores, and the final coloration, instead of simply absorbance or gravimetric yield.

#### 4.7.2 Organic-solvent extraction

Ethanol, methanol, acetone, ethyl acetate, chloroform, dichloromethane, tetrahydrofuran, dimethylformamide, dimethyl sulfoxide, isopropanol, and toluene were used for the extraction of poorly water-soluble plant and microbial pigments.^20,44,57,68,71,79,80,104,110,112,113,119,122,129,133,134,140,157,159,160,166,167,170,172^ Extraction treatments generally ranged from room temperature to approximately 80 °C for 30 min–5 h, while Soxhlet extraction commonly continued for 1–3 h.^68,133,134^

Organic solvents improved pigment selectivity and recovery. For example, methanol extraction of madder yielded 2.45 g of dark-red extract from 10 g of root, compared with only 0.14 g obtained using ethanol before preparative HPLC purification.^68^ However, the higher-yielding solvent was also the less desirable option from a health and environmental perspective. Therefore, ethanol and hydroethanolic mixtures provide a more defensible route than methanol, chloroform, dichloromethane, or tetrahydrofuran, but still require solvent recovery, fire-safe equipment, and residual-solvent control. These factors were rarely quantified.

#### 4.7.3 Microwave-assisted preparation

Microwave irradiation was used for pigment extraction, simultaneous extraction-dyeing, extract or fabric treatment, and microbial strain development. These functions are technically distinct. True microwave-assisted extraction or combined extraction-application generally used exposure periods of 1–10 min; reported conditions included 2450 MHz and 650–750 W, although several studies provided only percentage-power settings.^13,67,83,84,127,170^

A direct turmeric comparison produced an extraction efficiency of 78.35% ± 2.62% after 10 min of microwave treatment, compared with 53.11–68.38% for continuous ultrasound and approximately 68.63–69.02% for intermittent ultrasound.^67^ Because the extraction medium also contained ethanol and kojic acid, the improvement cannot be attributed exclusively to microwave heating.

Combined treatment of annatto extract and nylon produced *K*/*S* = 29.52 after 4 min.^83^ Harmal, saffron/safflower, *Cassia obovata*, and lac systems similarly showed optimum exposures of approximately 3–6 min, followed by a lower color yield upon longer treatment.^13,84,127,170^ Therefore, excessive irradiation may degrade chromophores, promote aggregation, or extract competing non-colorant compounds.

Microwave irradiation applied during dyeing after conventional extract preparation enhanced the coloration stage rather than extraction,^92^ while microwave mutagenesis of *Serratia* increased microbial pigment productivity rather than pigment recovery.^161^ Although microwave processing considerably shortened some treatments, electricity consumption per unit of pigment or fabric, thermal uniformity, and scale-up efficiency were rarely reported. Therefore, shorter processing time alone does not establish lower energy demand.

#### 4.7.4 Ultrasound-assisted preparation

Ultrasound was employed for extraction, pigment purification, nanoparticle preparation, dispersion, and dyeing. Extraction treatments generally ranged from 5 to 45 min and from room temperature to approximately 60 °C.^37,67,115,122,123,173,174^*Scrophularia* was sonicated for 15 min at room temperature,^173^*Verbascum* extraction was optimized at 15 g L^−1^ for 5 min at 30 °C,^174^ and saffron-stamen extraction reached an effective plateau at 50 °C and 30 min.^115^ Hawthorn extraction was optimized at 8 g L^−1^, pH 5, 60 °C, and 40 min using a water-to-ethanol ratio of 1 : 4.^122^

The outcome was dependent on the source. Selected phenolic and tannin recoveries from coffee and peanut residues using ultrasound were found to be higher,^37^ but not for all phytochemical fractions in comparative botanical systems.^123^ Sonication also enhanced the purity of bio-indigo from about 60% to 90% at 30 kHz and 100 W for 30 min,^80^ facilitated the preparation of curcumin nanoparticles,^172^ and helped wash or disperse nanoparticles.^42,71^

The main effect of ultrasound, when applied directly during dyeing, was enhanced mass transfer and nylon uptake rather than extraction.^37,43,92^ The nominal equipment powers reported in one study were 150 W for ultrasonic treatment and 3000 W for a conventional water bath,^37^ but these values do not represent net energy savings without taking into account treatment volume, treatment time, conversion efficiency, and delivered energy.

Cavitation can enhance solvent penetration, cell disruption, and mass transfer, but performance is dependent on the frequency, power density, solvent, vessel geometry, biomass loading, and exposure time. The lack of complete reporting of these variables hinders the ability to evaluate scale-up and reproducibility.

#### 4.7.5 Enzymatic and fermentation-based preparation

Enzymatic routes comprised the oxidation of phenolic precursors with horseradish peroxidase, enzymatic biosynthesis of indigoids, amylase modification of compounds related to weld, and enzymatic isolation or transformation of fungal anthraquinones.^32–34,79,165,167^ These methods can lead to the generation or modification of pigments under mild conditions; however, the environmental performance is determined by the enzyme production, oxidant consumption, catalytic yield, enzyme stability, and recovery.

Bacterial and fungal pigments were obtained by various fermentation methods, including direct application of broth or nanosuspensions, as well as solvent extraction.^34,36,44,80,157–164,166^ The cultivation period typically lasted for several days, and downstream processing comprised biomass separation, solvent extraction, evaporation, and redissolution. Therefore, the process yielding the highest pigment titer is not always the most promising microbial route, as it must not only be productive, but also employ safe microorganisms, simple media, short cultivation times, and minimal purification.

#### 4.7.6 Concentration, drying, and direct application

Filtration followed by direct use of crude extract avoided concentration and drying, but left the active-chromophore concentration and non-colorant load uncertain. Direct addition of plant powder to the dyebath eliminated a separate extraction stage in some studies,^122,144^ although suspended solids may impair levelness, filtration, machine cleanliness, and reproducibility.

Rotary evaporation, reduced-pressure concentration, water-bath evaporation, oven drying, vacuum drying, hot-air drying, freeze-drying, ultrafiltration, crystallization, and spray drying were used to improve storage, dosing, or purity.^33,37,44,64,70,72,82,100,119,128–130,138–140,145,146,148,150,157,177^ Sea-buckthorn extract was concentrated at 80 °C and freeze-dried at −40 °C for three days, yielding 14.72% powder,^72^ while beetroot liquor was concentrated at 40–50 °C to approximately 50–60% of its initial volume.^177^

These operations improve standardization and shelf stability but introduce additional thermal, vacuum, membrane, or refrigeration demands. Therefore, extraction and preparation routes should be selected according to marker-compound recovery, extract stability, solvent and energy consumption, residual-biomass management, and compatibility with textile equipment rather than extraction yield alone. The relationships among natural-colorant source, chemical class, preparation route, operating conditions, and principal limitations are summarized in Table 1.

**Table 1:** Relationships among natural colorant source, chemical class, extraction route, and main limitations

Colorant family	Main sources	Principal compounds	Preparation routes	Reported operating window	Typical shades	Expected nylon interaction	Critical advantage	Critical limitation	Ref.
Plant polyphenols	Leaves, bark, fruits, peels, seeds, and agricultural residues	Tannins, flavonoids, and phenolic acids	Water extraction, pH-assisted extraction, microwave, ultrasound, concentration	Room temperature – 100 °C; 5 min – 48 h; pH 2–11; approximately 1 : 4–1 : 40 solid-to-liquid ratio	Yellow, beige, brown, gray, and red-brown	Hydrogen bonding, pH-dependent attraction, and metal coordination	Broad availability and UV/antioxidant potential	Seasonal composition, high water demand, and weak marker-compound standardization	22, 35, 37, 43, 59, 60, 64, 96, 97, 115, 122, 139, 147, 155, 173 and 174
Plant quinones	Roots, bark, heartwood, and purified fungal or plant compounds	Anthraquinones and naphthoquinones	Water or alcohol extraction, solvent partitioning, chromatography, microwave treatment	Approximately 20–100 °C; 30 min – 3 h; route-specific purification	Orange, red, violet, and deep brown	Hydrogen bonding, hydrophobic association, aggregation, and metal complexation	Strong color depth and broad red–violet palette	Solvent burden, destructive harvesting, and variable photostability	22, 68, 79, 81, 110–114, 127 and 167
Curcuminoids	Turmeric and purified curcumin formulations	Curcumin, demethoxycurcumin, and bis-demethoxycurcumin	Alcoholic or aqueous extraction, microwave, ultrasound, nanoparticle preparation	Microwave 1–10 min; ultrasound commonly 5–45 min	Bright yellow to yellow–orange	Hydrogen bonding, hydrophobic partitioning, and dipole interaction	High brightness and biological activity	Poor water solubility, pH sensitivity, oxidation, and light fading	39, 42, 46, 67, 104, 105, 108 and 172
Carotenoids	Annatto, saffron, safflower, marigold, and tomato residues	Bixin, crocin, crocetin, carthamin, lycopene, and related pigments	Water, alcohol, or alkaline extraction; microwave and ultrasound	Microwave commonly 2–10 min; ultrasound approximately 30–60 °C for 5–45 min	Yellow, orange, pink, and red	Hydrophobic diffusion, van der Waals interaction, and limited polar association	Bright shades and functional potential	Oxidation, photofading, and poor aqueous solubility	13, 57, 83, 100, 115, 119, 121 and 146
Indigoids	Botanical indigo, biosynthetic indigo, and molluscan analogues	Indigo, indirubin, dibromoindigo, and related structures	Reduction, fermentation, enzymatic formation, purification	Multi-stage, route-dependent preparation	Blue, green, and purple	Hydrogen bonding, hydrophobic diffusion, and interaction of reduced ionic forms	Distinctive blue–purple shades	Low water solubility and dependence on reduction or biosynthesis	34, 41, 80, 85, 103, 157 and 171
Bacterial pigments	Bacterial cultures and engineered strains	Prodigiosin, violacein-type pigments, and indigoidine	Fermentation, clarification, solvent recovery, sonication-assisted purification	Multi-day cultivation; downstream recovery varied widely	Red, pink, purple, and blue	Hydrophobic diffusion, hydrogen bonding, and pH-dependent adsorption	Season-independent and tunable production	Nutrient, sterilization, aeration, biosafety, and purification demands	34, 36, 80 and 157–162
Fungal pigments	*Monascus*, wood-spalting fungi, *Dermocybe*, and *Aspergillus*	Anthraquinones and chemically mixed fungal pigments	Fermentation or biomass extraction, solvent partitioning, chromatography	Multi-day cultivation; pigment-dependent isolation	Yellow, orange, red, violet, and blue-green	Hydrophobic partitioning, hydrogen bonding, and aggregation	Distinct shades and multifunctionality	Long cultivation, solvent intensity, and crude-mixture variability	44, 79 and 163–167
Animal anthraquinones	Cochineal and lac insects	Carminic and laccaic acids	Aqueous, acidic, or alcohol-assisted extraction; selected microwave treatment	Boiling commonly around 60 min; selected microwave treatments approximately 3–7 min	Crimson, red, and purple	Ionic attraction, hydrogen bonding, and metal coordination	High tinctorial value and established shade chemistry	Cost, ethics, sensitization, scarcity, and mordant dependence	19, 61, 62, 78, 126 and 170
Modified natural-derived systems	Phenolic precursors, curcumin, plant extracts, and natural compounds combined with inorganic phases	Oxidized phenolic polymers, nano-curcumin, Ag/ZnO hybrids, and azo derivatives	Enzymatic oxidation, sonication, nanoparticle synthesis, diazotization, freeze-drying	Route-specific; sonication commonly 5–30 min	Brown, yellow, orange, red, and purple	Surface deposition, entrapment, hydrogen bonding, and possible coordination or covalent interaction	Improved dispersibility, fixation, and multifunctionality	Additional chemicals, nanoparticle release, solvent burden, and end-of-life uncertainty	32, 33, 71, 87, 172–174, 176 and 177

Overall, no preparation route can be regarded as universally cleaner. Water avoids hazardous organic solvents but may require prolonged heating and high liquor volumes; organic solvents improve selectivity but create safety and recovery burdens; microwave and ultrasound shorten selected processes but remain poorly characterized in terms of net energy consumption; and fermentation improves supply control while introducing cultivation and downstream-processing demands. Therefore, the most appropriate route should be selected through a multi-criteria assessment of pigment yield and quality, resource consumption, chemical safety, waste generation, process scalability, and compatibility with nylon coloration.

## 5 Dyeing, fixation, and process-intensification strategies

### 5.1 Conventional exhaust dyeing

Conventional exhaust dyeing remains the dominant route for applying natural colorants to nylon. The typical sequence includes scouring, dye-liquor preparation and filtration, pH adjustment, controlled heating, dyeing, cooling, rinsing, soaping, and drying. Most studies were conducted at pH 3–6, 80–100 °C for 45–60 min, and a material-to-liquor ratio of 1 : 20–1 : 50.^20,40,59,77,89,96,115,121,132,140^ Ratios of 1 : 10 were demonstrated in selected production-oriented processes,^73,76,121,136^ including jet dyeing at 98 °C for 45 min,^76^ whereas ratios of 1 : 100 or higher were used mainly for laboratory adsorption studies.^61,93,100,109,152,176^ After washing was inconsistent, with some studies using hot soaping at 60–90 °C,^35,36,40,59,97,148,150^ while others reported only rinsing and drying.^20,89,98,117,175^ Because residual surface colorant can inflate *K*/*S* but impair wet fastness, the omission of information on extract concentration, liquor ratio, agitation, replication, or cleaning conditions substantially weaken comparison. The major process variables should therefore be considered as an interacting system rather than in isolation.

### 5.2 Effect of dye-bath pH

Dye-bath pH was generally the most influential chemical variable. Most phenolic, flavonoid, tannin-rich and anionic colorants performed best at pH 3–5.8, where protonated nylon amino end groups form –NH_3_^+^ sites for electrostatic and hydrogen-bonding interactions. Rice-straw extract gave *K*/*S* values of 6.6 on untreated and 10.7 on plasma-treated nylon 6 at pH 4.^20^ Olive-leaf *K*/*S* changed from 17.62 at pH 4.0 to 20.09 at pH 5.8, 13.77 at pH 7.0 and 8.17 at pH 9.0,^132^ while *Terminalia catappa* gave a *K*/*S* of 21.53 at pH 3 but only 0.49 at pH 11.^96^*A. nobilis* similarly decreased from *K*/*S* of 2.10 at pH 4.5 to 0.81 at pH 10.^114^ Sea-buckthorn showed a non-linear optimum of *K*/*S* of 17.999 at pH 4.16; both stronger acidity and alkalinity reduced uptake, with *K*/*S* falling to 4.979 at pH 11.34.^72^ Thus, lower pH does not invariably produce greater color depth.

Strong acidity may maximize *K*/*S* but weaken the overall process balance. Babool bark reached *K*/*S* of 7.3 at pH 2, although pH 5 was recommended to limit polyamide hydrolysis.^59^ Baicalin likewise peaked near pH 2 but was practically applied at pH 4.^63^ Annatto at pH 2.5 gave reported *K*/*S* values of 167.68 without soy milk and 191.40 with soy treatment, although soy treatment showed poor levelness, with Δ*E* = 7.82.^57^ Monascus pigment achieved the best depth-strength balance near pH 3.5.^164^ Fastness also remained pigment-dependent, as lemon-assisted neem dyeing at pH 4.5 gave *K*/*S* of 16.5 and washing fastness 4,^90^ whereas spent-coffee-ground dyeing at pH 4 showed washing fastness of 4/5 but light fastness of only 1/2.^140^ Sea-buckthorn similarly retained light fastness of only 1–2 to 2 despite its high *K*/*S*.^72^

Alkaline conditions were justified for specific colorant chemistries. Henna was applied at pH 9, whereas pomegranate rind and *Pterocarya fraxinifolia* were applied at pH 3.^22^ Reduced indigo required approximately pH 11–12.^41,80^ Cationic berberine exhaustion increased from 0.0291% at pH 3 to 76.56% at pH 12, and *K*/*S* increased from 0.20 to 2.95 because alkaline nylon carboxylate sites attracted the dye;^66^ modified betalains also developed greater depth near pH 9.^177^ Accordingly, pH should be selected based on nylon charge, colorant pK_a_, solubility and stability, while recognizing that both highly acidic and alkaline conditions increase neutralization demand and may damage the fiber or pigment.

### 5.3 Effect of dyeing temperature

Most systems achieved the best balance at 80–100 °C. Above the hydrated nylon glass-transition region, reported near 50–57 °C, segmental mobility, free volume and swelling accelerate diffusion.^40,66^ Mangrove-bark *K*/*S* increased from approximately 1.66 at 30 °C to 5.38 at 90 °C;^40^ another polyamide system increased from *K*/*S* of 1.32 at 40 °C to 8.12 at 100 °C,^137^ while pomegranate-peel *K*/*S* increased from 1.08 to 3.99 over the same temperature range.^147^ Nevertheless, gains often diminished above 90–100 °C. *T. catappa* increased from *K*/*S* of 11.67 at 80 °C to 21.53 at 100 °C but only to 21.94 at 120 °C, meaning that the additional 20 °C yielded approximately 1.9% greater color depth.^96^ Plasma-assisted indigo likewise showed little additional benefit above 50 °C.^41^

High temperatures occasionally increased *K*/*S* at the expense of durability. Babool-bark dyeing yielded a *K*/*S* value of 18.0 at 140 °C, whereas at 100 °C the dyeing yield was *K*/*S* of 6.8, but SEM showed surface roughness and microcracking.^59^*A. nobilis* colorant loss at 130 °C approached 90–100%, depending on pH,^114^ while purpurin absorbance loss increased from approximately 23% at 100 °C to 82% at 130 °C.^112^ Prodigiosin peaked near 80 °C and declined at 90 °C,^36^ and curcumin nanoparticles performed better at 70 °C than at 100 °C.^172^ Excessive heat may also cause nylon hardening, shrinkage, handle loss and uneven migration even without visible cracking. Therefore, temperature optimization should include *K*/*S*, levelness, fastness and mechanical retention. PA56 may permit diffusion at lower severity because of its lower crystallinity and higher free volume,^70^ whereas nylon 5,10 was colored enzymatically near 55 °C because of its lower thermal stability.^33^

### 5.4 Dyeing time, dye concentration, and liquor ratio

Dyeing times of 45–60 min were most common, although slower systems required 90–120 min. Babool-bark *K*/*S* increased from 4.4 at 30 min to 6.0 at 60 min and only to 6.6 at 120 min,^59^ whereas extending *T. catappa* dyeing from 60 to 90 min reduced *K*/*S* from 21.53 to 20.94.^96^ Cochineal required approximately 240 min to reach equilibrium at 60 °C but only 120 and 90 min at 80 °C and 100 °C, respectively;^61^ another system increased from *K*/*S* of 2.28 at 45 min to 8.23 at 90 min.^137^ Therefore, time must be optimized jointly with temperature.

Concentration increased depth until site saturation, aggregation or solubility limitations dominated. Brazilwood showed color build-up to approximately 60% owf;^98^ potassium norbixinate maintained 93.4–98.4% exhaustion from 0.25% to 4% owf while *K*/*S* increased from 0.4 to 6.9.^121^ Papaver extract improved up to 100% owf but declined at 200% owf,^117^ modified betalains showed little gain above 2.0%,^177^ and another series gained only 0.33 *K*/*S* units between 7% and 9% owf.^137^ Typical liquor ratios ranged from 1 : 20 to 1 : 50. Berberine exhaustion increased from 65.59% to 72.14% when the ratio decreased from 50 : 1 to 20 : 1,^66^ but mullein dyeing peaked near 1 : 30 rather than 1 : 10 or 1 : 50.^118^ Productivity decreases as conditions mover further away from near-equilibrium and with further additions of dye or water, or with prolonged treatment time, when these changes do not produce a corresponding increase in *K*/*S* or fastness.

### 5.5 Mordant-free dyeing

Mordant-free coloration relies on protonated amino groups, hydrogen bonding, hydrophobic association, internal diffusion or *in situ* pigment formation. Olive-leaf extract gave *K*/*S* of 18.60 with excellent washing, perspiration and crocking performance.^132^ Optimized *T. catappa* dyeing produced a *K*/*S* of 21.94, washing fastness of 4–5, light fastness of 5 and UPF of 1995;^96^ hawthorn gave approximately a *K*/*S* of 6.5 with washing fastness of 4–5 and light fastness of 7,^122^ while bio-indigo reached a *K*/*S* of 12.3 and fastness values generally between 4–5 and 5.^80^

However, high color depth did not ensure photostability. Spent-coffee-ground dyeing gave a *K*/*S* of 3.18 and washing fastness of 4/5 but light fastness of only 1/2;^140^ sea-buckthorn reached a *K*/*S* of 17.999, yet its light fastness remained at 1–2 to 2,^72^ and unmordanted mangrove bark showed a washing color change of 1–2 and light fastness of 2.^40^ Enzymatic pigments achieved moderate *K*/*S* values of approximately 3.0–4.5,^32,33^ while microbial or vat systems could require alkali, reducing agents or long reaction times.^34,80^ Therefore, mordant-free means the absence of an external mordant, not inherently a low environmental impact.

### 5.6 Metallic mordanting

The principal metallic mordants were aluminium sulfate or alum, ferrous and ferric sulfates, copper sulfate and stannous chloride. Potassium dichromate or other chromium salts, zinc sulfate and cobalt sulfate were also evaluated, while magnesium sulfate and calcium carbonate occurred mainly in broader nylon mordant screens rather than in the strongest natural-dye evidence.^21,22,40,74,77–79,102,115,117,120,130,131,133,135,137,139,145,148,150,151^ Binary combinations included alum–iron, alum–tin and iron–tin.^74^ Typical additions were 0.2–10% owf or g L^−1^ using pre-, simultaneous/meta- or post-mordanting.

Iron most consistently darkened shades and increased *K*/*S*. Simultaneous mordanting increased the *K*/*S* of nylon 6 from 10.6 without mordant to 14.7 with CuSO_4_, 12.6 with SnCl_2_, and 15.8 with FeSO_4_; light fastness increased from 4 to 6–7 or 7.^133^ FeSO_4_ increased *K*/*S* from 4.05 to 6.11 in another system, whereas alum reduced it to 3.72.^137^ Teak-leaf dyeing gave a *K*/*S* of 3.275 with iron, 2.837 with copper and 1.242 with alum.^139^ However, iron–tannin complex formation can darken the shade without a proportionate increase in fiber uptake.

Copper often improved depth, light fastness, or bioactivity but shifted shades towards darker or greener tones. Olive-leaf post-mordanting gave a *K*/*S* of 20.82,^132^ and copper improved saffron-stamen light fastness from 6–7 to 7 and washing color change from 4 to 4–5.^115^ Alum generally retained brighter shades but exhibited inconsistent performance, as the *K*/*S* of saffron-stamen and weld decreased with aluminium and tin.^115^ Potassium alum increased the color intensity of PA6.6 by only 0.55% without improving washing fastness,^152^ whereas aluminium post-mordanting increased the *K*/*S* of mangrove bark to 6.75, approximately 32% above the unmordanted sample.^150^

Chromium has sometimes yielded the highest *K*/*S*, for example around 12.29 compared with 11.65 for copper in one study^102^ and *K*/*S* of 8.46 with light fastness of 7 in another,^131^ but the toxicity and regulatory issues are too substantial to compensate for the color-depth advantage. Zinc was generally less effective, while cobalt modified the anthraquinone shade and fastness without establishing a generally superior process.^21,79,131^ Ferric sulfate produced a 100% *S. aureus* reduction in walnut-dyed nylon, although no corresponding *K*/*S* advantage was demonstrated.^145^ Sequence was also decisive, with post-mordanting performing best for mangrove bark,^150^ cochineal performance varying by metal,^78^ and 0.8 g L^−1^ SnCl_2_ producing a *K*/*S* of 2.89 and Δ*E* of 11.37 by simultaneous mordanting but a *K*/*S* of 3.32 and Δ*E* of 4.27 by premordanting.^74^ Therefore, metallic mordants should be justified through combined *K*/*S*, fastness, shade, and residual-metal data rather than depth alone.

### 5.7 Biomordants and bio-based fixation agents

Polymeric and proteinaceous systems included chitosan, chitin-derived cationisers, sodium alginate, soy milk and silk-fibroin residues.^19,33,35,57,76,88,168,178^ Phenolic or tannin-rich agents included tannic acid, cassava-leaf tannin, mimosa, pomegranate peel or rind, myrobalan/*Terminalia chebula*, harda, walnut husk, pinecone, pistachio hull, marble or oak gall, eucalyptus, acacia, peppermint, *Artemisia*, *Dorema ammoniacum* gum, turmeric, rosemary, nettle, supari, tamarind and amla.^22,42,82,83,102,117,118,120,123,125,127,130,131,137,139,140,142,143,153,170^ Lemon juice, aloe-vera gel, rhubarb extract, l-cysteine, and citric, tartaric or oxalic acids were also used;^70,74,76,82,90,124,158^ ammonium sulfate and sodium acetate are better classified as non-metal auxiliary salts.^74^

Chitosan was effective only when the treatment sequence was controlled. *Ex situ* chitosan increased gallic-acid *K*/*S* from 4.0 to 4.5, whereas *in situ* catechol/chitosan treatment gave a *K*/*S* of 1.5 because of uneven polymer deposition.^33^ Chitosan premordanting of a tea-dyed nylon/cotton blend gave a *K*/*S* of 4.090, washing fastness of 4–5 and light fastness of 5–6,^88^ and a chitosan coating enabled Carmine Red coloration that was negligible on untreated PA6.6.^168^ However, overnight impregnation and curing at 95 °C and 150 °C shifted rather than eliminated energy demand.

Proteinaceous systems were dye-specific. Soy milk increased annatto depth, but the sample with a *K*/*S* of 191.40 showed poor levelness and rubbing transfer;^57^ another study reported a *K*/*S* of 108.55 for soy-assisted eucalyptus and 251.24 for lemon-assisted annatto.^91^ Silk-fibroin residues improved levelness but reduced uptake,^178^ while sodium alginate acted mainly as a film-forming layer, giving *K*/*S* values of 17.96–20.02 compared with 18.84–19.87 for tannic acid.^132^

Tannic acid was the best evidence for metal replacement. At 20%, it produced a henna *K*/*S* of 16.50 and light fastness of 6; a pomegranate-rind of *K*/*S* 10.74, almost identical to 10.75 with tin; and a *P. fraxinifolia K*/*S* of 17.02 with a light fastness of 6.^22^ It also improved almond-stem *K*/*S* from 12.92 to 14.84 without affecting the washing fastness of 4–5.^143^ However, higher tannin deposition did not lead to better washing fastness for another PA6.6 system, and it caused a decrease in the acid perspiration color change from 2 to 3.^152^

Plant biomordants were frequently competitive. The walnut husk and pinecone yielded *K*/*S* of 18.77 and 18.62 with washing fastness of 5 and light fastness of 8,^120^ while the walnut husk and *T. chebula* gave *K*/*S* of 12.5 and 12.2, respectively, with beetroot.^130^ Pomegranate peel increased *K*/*S* from 8.91 at 10% to 14.59 at 50% owf, whereas pistachio hull reduced depth despite selected fastness gains.^118^*Artemisia* and *Dorema* gum gave a *K*/*S* of approximately 13.18, exceeding chromium and copper in the same study, and *Artemisia* provided light fastness of 7.^102^ Acacia reached a *K*/*S* of approximately 34.7 by pre-biomordanting, while turmeric performed better as a post-biomordant, reaching a *K*/*S* of approximately 33.2.^170^

Durability gains were not always linked to maximum depth. Mimosa reduced *K*/*S* from 4.05 to 3.19 but raised washing and light fastness to 5;^137^ cassava tannin increased *K*/*S* only from 3.18 to 3.32 and light fastness from 1/2 to 2;^140^ and aloe vera improved light fastness by 1–1.5 grades.^70^l-cysteine also provided better wash retention than the higher-depth deep-eutectic-solvent route.^158^ True metal-free biomordanting should be distinguished from hybrid bio/metal systems, which reduce but do not replace metals. High dosage, extraction, curing, variability, COD and washing must remain part of the assessment.

Despite their lower metal burden, biomordants are not necessarily low-cost or operationally simple. Direct cost evidence is scarce, but adding 20% tannic acid increased henna dyeing cost from 0.42–0.44 to 1.08–1.14 US$ per kg nylon.^22^ Plant-derived biomordants may also require relatively high dosages and additional extraction or pretreatment, while variation in extract composition can complicate batch-to-batch shade reproducibility. Chitosan, soy-protein and alginate systems can form surface layers or need long impregnation and curing times, so the effects on the durability of the fabric handle and coatings must be evaluated rather than assumed to be negligible.^33,35,57^ Protein- and polyphenol-rich auxiliaries may also increase the organic load of spent baths, although COD/BOD and biodegradability are rarely quantified. Therefore, biomordant selection should balance fixation performance with dosage, cost, reproducibility, processing requirements, fabric quality, and effluent burden.

Because conventional optimization cannot fully overcome nylon diffusion limitations or pigment instability, physical, biological and low-water intensification approaches have been explored. Its value depends on whether performance gains occur without transferring burdens to electricity, solvents or specialized recovery systems.

### 5.8 Microwave-assisted pretreatment and dyeing

Microwave treatment was used for nylon pretreatment, extract irradiation and simultaneous extraction–dyeing, generally for 1–10 min with optima at 4–6 min.^13,83,84,127,170^ Microwave-treated annatto reached a *K*/*S* of approximately 8.4 after 4 min,^127^ and *Cassia obovata* gave a *K*/*S* of 5.1138 after 6 min.^84^ Exhaustion increased from 63% conventionally to 93% by microwave, while dyeing time fell from 120 to 40 min at the reported 90% power;^92^ simultaneous curcumin extraction–dyeing required 10 min and achieved an extraction efficiency of 78.35% ± 2.62%.^67^

Microwaves accelerate swelling and diffusion without necessarily altering the principal nylon FTIR bands.^83,84,127^ However, the reported percentage power, missing load data, and inadequate reporting of temperature uniformity prevent reliable kWh kg^−1^ comparisons. Local overheating, shade variation and fiber damage remain scale-up risks; shorter processing time alone is insufficient evidence of cleaner production.

### 5.9 Ultrasound-assisted dyeing

Ultrasonic cavitation breaks down the boundary layer and enhances mass transfer. Nylon exhaustion increased from 63% conventionally to 81% with ultrasound.^92^ An ultrasonic simultaneous dyeing–mordanting technique yielded Δ*E* values up to 23.6 and fastness grades of up to 5 at a liquor ratio of 1 : 50 and a dyeing temperature of 30 °C for 30 min.^82^ Olive-wastewater dyeing showed that *K*/*S* values increased from ∼6.35 to 7.21 when the power was raised from 100 W to 500 W, the selected fastness grades improved by about half a grade, and the polyphenols and COD were reduced.^43^

The potential for savings is reported for ultrasound operated at 150 W and for a water bath at 3000 W,^37,123^ but the rating of the equipment does not equate to energy delivered. Frequency, intensity, bath geometry, and loading were routinely neglected, and a single bath system had a bath volume of only 0.8 L.^82^ Too much cavitation can cause deterioration of pigments; industrial use will need uniform flow-through sonication and measured specific energy.

### 5.10 Plasma, laser, electron-beam, and radiation-assisted surface activation

Atmospheric plasma treatment, dielectric-barrier discharge, and corona treatment improved the wettability, surface roughness and polar surface groups. Air plasma at 45 W for 15 min increased rice-straw *K*/*S* from 6.6 to 10.7,^20^ while DBD was optimized at 375 W for 2 min 41. Five semi-industrial passes at 4 m min^−1^ resulted in an increase in the PA66 washing fastness from 4 to 5, with conflicting reported power values ranging from 1 W to 1 kW, making reproducibility difficult.^93^ Corona treatment at 50 W for 30 s on each side resulted in less than 1% loss of breaking load.^65^

Laser treatment increased the *K*/*S* value from 9.7 to 15.1 at 12 W, but the value decreased at 16–20 W due to melting and fiber attachment.^45^ Electron-beam irradiation gave a *K*/*S* of 18.1 at 300 kGy with an 8% strength loss.^105^ These dry routes are effective at reducing wet chemicals, but overexposure can result in chain scission or embrittlement, and plasma ageing and continuous process consistency have yet to be fully evaluated.

### 5.11 Enzymatic and biologically mediated coloration

Horseradish peroxidase/H_2_O_2_ converted catechol, hydroquinone or gallic acid into polymeric pigments near 55 °C, producing *K*/*S* values up to 3.4 without and up to 4.5 with *ex situ* chitosan.^32,33^ Laccase and tyrosinase are mechanistically relevant, but nylon-specific numerical evidence remained limited. Pepsin or trypsin pretreatment at 1–5% for 60–120 min did not consistently improve dyeability.^144^

Biological *in situ* coloration produced leuco indigoids at 30 °C over 24 h and avoided a separate reduction bath, whereas the chemical comparator required 15 min at 60 °C.^34^ Crude bacterial extracts or fermentation broth can reduce purification requirements,^157,160^ but DMSO or sodium hydrosulfite remained necessary in some routes. Enzyme cost, oxidant use, stability, long reaction times, sterility and batch control constrain scale-up.

### 5.12 Nanoformulation, deep eutectic solvents, supercritical CO_2_, and low water application

Nanoformulation improved the dispersion of curcumin and microbial pigments.^163,172^ Green-synthesized Ag nanoparticles increased the *K*/*S* of nylon from 1.12 to 3.36, gave light fastness of 5 and achieved complete antibacterial reduction, but nanoparticle release during washing was not quantified.^174^ Choline chloride/lactic acid at a 1 : 2 ratio as a deep eutectic solvent gave a *K*/*S* of 3.33 at 60 °C for 60 min, yet the washing Δ*E* was 10.77 compared with 3.21 for lower-*K*/*S*l-cysteine,^158^ showing that water-free application did not ensure fixation.

Supercritical CO_2_ at 110–120 °C and 3500–5000 psi eliminated the aqueous bath,^68,165^ but pressure, cost and colorant solubility limit scale-up. D5-assisted Monascus dyeing increased *K*/*S* from 1.66 to 2.64,^164^ whereas linseed oil reduced process water but depended on dichloromethane and showed poor long-term fixation.^166^ Dope coloration avoided wet dyeing but required solvent-intensive nanoparticle preparation.^71^ Digital printing enabled localized curcumin deposition,^42^ while lowering the liquor ratio from 50 : 1 to 20 : 1 increased berberine exhaustion from 65.59% to 72.14%.^66^ Therefore, total solvent recovery, washing, release and energy must be assessed. A comparative technical and cleaner-production assessment of the principal dyeing and process-intensification strategies is presented in Table 2.

**Table 2:** Comparative technical and cleaner-production assessment of dyeing and process-intensification strategies

Strategy	Representative operating conditions	Main mechanism	Technical outcome	Water or energy benefit	New burden or limitation	Scale-up status	Ref.
Conventional exhaust dyeing	Commonly pH 3–6, 80–100 °C, 45–60 min and LR 1 : 20–1 : 50; selected systems at 1 : 10	Protonation, adsorption and internal diffusion	Broad applicability; *K*/*S* usually increases with acidity and temperature until equilibrium or degradation	Established equipment and process control	High water, heating, rinsing and soaping demand	Industrially established	20, 40, 59, 76, 77, 96, 115 and 132
Mordant-free dyeing	Usually pH 3–5 and 70–100 °C; alkaline for indigo and cationic dyes	Ionic attraction, hydrogen bonding, hydrophobic association or *in situ* pigment formation	*K*/*S* = 18.60 for olive leaf and 21.94 for *T. catappa*; wet fastness up to 4–5, but poor light fastness in some systems	Avoids metal salts and metal-bearing effluent	May require strong pH control, reduction, heat and repeated washing	Laboratory to pilot	40, 72, 80, 96, 122, 132 and 140
Metallic mordanting	Commonly 0.2–10% owf or g L^−1^; pre-, simultaneous/meta- or post-mordanting	Dye–metal–fiber coordination and metal–tannin complexation	FeSO_4_ increased *K*/*S* from 10.6 to 15.8 and light fastness from 4 to 7; alum, tin and zinc effects were dye-dependent	Can improve fixation and reduce unfixed dye	Toxicity, residual-metal release, sludge and shade alteration	Technically mature but increasingly restricted	22, 74, 78, 79, 102, 115, 120, 131, 133, 135, 137, 139, 145 and 150
Biomordanting	Approximately 1–50% owf; pre-, simultaneous or post-application	Additional phenolic, cationic, proteinaceous or film-forming sites	Walnut husk and pinecone gave *K*/*S* = 18.77 and 18.62 with washing fastness = 5 and light fastness = 8	Renewable or waste-derived alternatives to metals	High dosage, extraction, variability, COD, curing and washing	Mainly laboratory or pilot	22, 33, 42, 57, 70, 88, 90, 102, 117, 118, 120, 130, 137, 140, 143, 158 and 170
Microwave-assisted dyeing	Generally 1–10 min irradiation; optima frequently 4–6 min	Volumetric heating and accelerated diffusion	Exhaustion increased from 63% to 93%; *K*/*S* was approximately 8.4 after 4 min in one system	Major reduction in treatment time	Non-uniform heating and insufficient specific-energy reporting	Laboratory	13, 67, 83, 84, 92, 127 and 170
Ultrasound-assisted dyeing	Approximately 30–100 °C, 30–120 min and 100–500 W	Cavitation and boundary-layer disruption	*K*/*S* increased to approximately 6.35–7.21; selected fastness grades improved by about 0.5 grade	Potential reduction in temperature or duration	Scale-dependent acoustic distribution and pigment degradation	Bench or laboratory	37, 43, 82, 92 and 123
Plasma activation	Air plasma, DBD or corona; seconds to 15 min	Surface oxidation, roughening and increased wettability	Rice-straw *K*/*S* increased from 6.6 to 10.7; washing fastness increased from 4 to 5 in a separate study	Dry and rapid surface activation	Surface ageing, high voltage and possible over-etching	Laboratory to semi-industrial	20, 41, 65 and 93
Laser activation	Approximately 4–20 W with controlled scanning	Local thermal or photochemical morphology modification	*K*/*S* increased from 9.7 to 15.1 at 12 W but declined at higher powers	Dry, localized and patternable	Fiber melting, adhesion and limited penetration	Laboratory or pilot patterning	45
Electron-beam activation	Up to approximately 300 kGy	Radical generation and surface functionalization	Maximum *K*/*S* = 18.1 with an 8% breaking-force loss	Rapid treatment without wet initiators	Shielding, capital cost and strength loss	Laboratory	105
Enzymatic coloration	HRP/H_2_O_2_ at approximately 55 °C for up to 3 h	Oxidative polymerization of phenolic precursors	*K*/*S* up to 4.5 with *ex situ* chitosan	Mild temperature and no metallic mordant	Enzyme cost, oxidant use and long reaction time	Proof-of-concept	32, 33 and 144
Microbial *in situ* coloration	Approximately 30 °C for up to 24 h	Biosynthesis and simultaneous pigment formation or fixation	Avoids separate pigment synthesis or reduction in selected systems	Lower processing temperature and fewer isolated stages	Long reaction, sterility and biological control	Proof-of-concept	34, 157 and 160
Nanoformulation	Aqueous nano-dispersions or biosynthesized nanoparticles	Improved dispersion, surface area and entrapment	AgNP-assisted *K*/*S* = 3.36, light fastness = 5 and 100% antibacterial reduction	Can combine coloration and functional finishing	Nanoparticle release and toxicological uncertainty	Laboratory	163, 172 and 174
Deep eutectic solvent	ChCl/LA 1 : 2, 60 °C and 60 min	Nonaqueous pigment solubilization and transport	*K*/*S* = 3.33, but washing Δ*E* = 10.77	Eliminates the aqueous dye bath	High viscosity, solvent recovery and after washing	Early laboratory	158
Supercritical CO_2_	Approximately 110–120 °C and 3500–5000 psi	CO_2_ plasticization and nonaqueous diffusion	Water- and mordant-free PA66 coloration	No conventional dye-bath wastewater	High pressure, capital cost and restricted natural-dye solubility	Laboratory or pilot	68 and 165
Low-liquor or solvent-assisted dyeing	LR down to 1 : 10–1 : 20; D5 or oil-based carriers	Increased bath concentration or phase transfer	LR 20 : 1 gave 72.14% berberine exhaustion; D5 gave *K*/*S* = 2.64	Reduced water and heated-liquor volume	Solvent recovery, toxicity and washing requirements	Laboratory to technically scalable	66, 76, 164 and 166
Digital inkjet printing	Approximately 80 pL droplets and 300 dpi in the reported system	Localized drop-on-demand deposition	High-resolution curcumin coloration and functionalization	Low application liquor and suitability for short runs	Ink stability, nozzle performance, pretreatment and after washing	Industrial platform; natural-dye inks mainly laboratory-scale	42

### 5.13 Experimental optimization and predictive modeling

Many studies used one-factor-at-a-time optimization,^44,95,96,115,117,118,120,178^ which identifies trends but cannot quantify factor interactions or distinguish the maximum *K*/*S* from a resource-efficient optimum. More rigorous studies used fractional factorial designs,^172^ central composite designs,^83,84,127^ Box–Behnken designs,^60,102^ desirability analysis, artificial neural networks, genetic algorithms and fuzzy logic. A reduced Box–Behnken model gave *R*^2^ = 0.9890, adjusted *R*^2^ = 0.9829 and predicted *R*^2^ = 0.9710.^102^ An ANN–GA model achieved a testing *R*^2^ of 0.9638 and predicted 68.6 wt% dye, 102.5 min, pH 5 and 95 °C; the predicted and validated *K*/*S* values were 11.89 and 11.5, respectively.^131^ Fuzzy modeling reduced prediction error by 9.1–16.7% relative to RSM.^94^

**Fig. 5:**
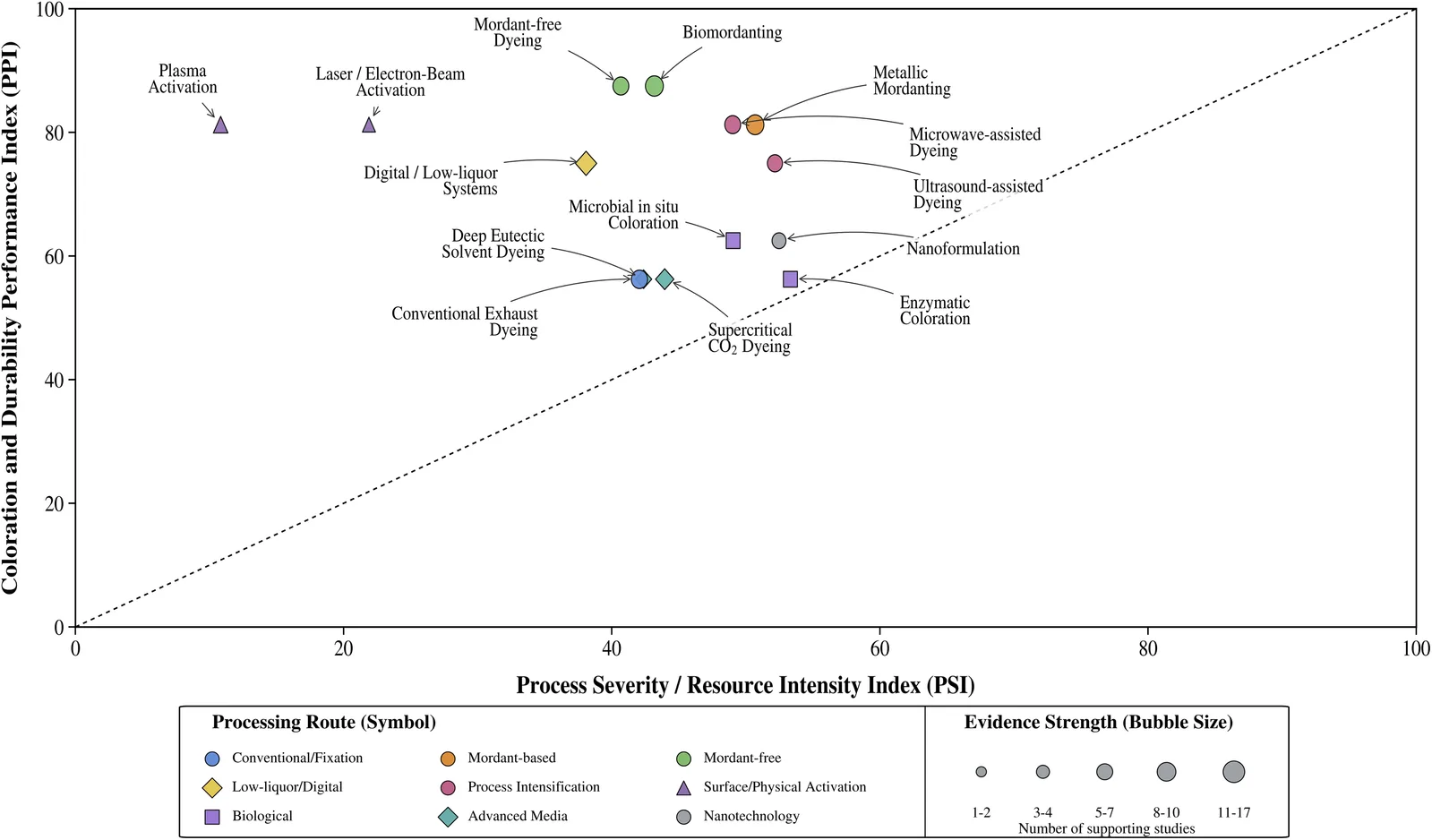
Process-performance-sustainability trade-off map for the natural dyeing of nylon.

Model fit did not always ensure practical consistency. One study reported *R*^2^ = 97.60% and desirability of 0.981 809, predicting 86.57 °C, pH 2 and 95 min, but discussed a different practical region near 65 °C, pH 5 and 60 min.^60^ A further CCD study was reported in which a practical recipe was separated from the model-predicted *K*/*S* maximum.^84^ Thus, it is crucial to have independent validation. Future multi-response models should include *K*/*S*, levelness, washing, rubbing and light fastness, mechanical retention, water, specific energy, auxiliary demand and effluent load. These process-performance-sustainability trade-offs are illustrated in Fig. 5.

Process severity or resource intensity is plotted along the *x*-axis; the *y*-axis represents coloration and durability performance, the bubble size represents the strength of evidence, and the type of symbol represents the fixation or the intensified-processing route. The map shows that the highest color strength does not necessarily reflect the cleanest process.

## 6 Dye–fiber interactions, adsorption, kinetics, and thermodynamics

The natural dye uptake by nylon is determined by a coupled process of bath speciation, pH-dependent surface charge, adsorption, diffusion into the amorphous regions of polyamide, and final retention by physical or chemical interactions. The reviewed evidence suggests that nylon does not have a single universal bonding mechanism, since the association of dyes with nylon is dependent on the charge on the dye, pH, molecular size, aggregation state of the dye, mordant chemistry, and fiber morphology. Hence, adsorption constants, kinetic fits and thermodynamic values should be regarded as indicative of the textile dyeing process rather than as evidence for a particular molecular bond.

### 6.1 Ionic and electrostatic interactions

Ionic attraction is the most recurrent explanation for nylon dyeability with phenolic, flavonoid, anthraquinone, lac, cochineal, pomegranate, sesame, baicalin, and other anionic or weakly acidic colorants. Terminal amino groups protonate to –NH_3_^+^ under acidic conditions, binding to carboxylate, phenolate, sulfonate, or lactone-derived anionic groups in dyes.^21,22,39,40,61,63,77,147^ The practical acidic range is usually pH 2–5.5 and is typically associated with acidic peaks at pH 3–4.5 rather than neutral pH. This is shown by pomegranate peel at pH 3,^147^ curcumin at pH 4 with 98.1–99.0% exhaustion over 1–6 h,^39^ sesame at pH 4,^77^ cochineal at pH 3 rather than pH 6,^61^ and baicalin, where the *K*/*S* of nylon decreased from about 4.4 at pH 2 to 1.3 at pH 12.^63^ A similar acid-favored behavior was observed with olive vegetable water and *Aspergillus* pigments.^43,44^

The acidic behavior is not a universal phenomenon, as the cationic berberine was found to act in the reverse way in the presence of nylon carboxylate sites formed in alkaline media, increasing exhaustion from 0.0291% at pH 3 to 76.56% at pH 12.^66^ Indigo also needs alkaline reduction prior to oxidative fixation.^70,171^ Bath ions cause additional complexity as chloride competes with dye anions for protonated nylon sites and decreases *K*/*S*, while sodium chloride had minimal effect on the *K*/*S* of baicalin, which stayed around 3.2–3.3 from 0 to 40 g L^−1^ dosing.^63^ Therefore, ionic interaction is significant, but its direction and intensity will vary with the charge states of the dye and nylon at the operating pH.

### 6.2 Hydrogen bonding and dipole interactions

Hydrogen bonding is widely proposed because nylon contains amide carbonyl oxygen and N–H groups, while natural colorants commonly contain phenolic hydroxyl, carboxyl, carbonyl, methoxy, pyrrole, quinone, or glycosidic groups.^20,44,59,63,98,104,106,110,147^ Plausible contacts include dye O–H⋯O=C (polyamide), dye C=O⋯H–N (polyamide), and dipole-assisted interactions between β-diketone, quinonoid, or methoxy-substituted chromophores and amide groups. However, chemical plausibility is not direct proof. Broadening of FTIR bands near 3300 cm^−1^ and amide I/II shifts after Babool bark dyeing indirectly support nylon–tannin interactions,^59^ and similar interpretations were made for Cassia, rice straw, emodin, and pomegranate systems.^20,84,110,147^ Nevertheless, many studies characterized only the dye powder or extract rather than the dyed nylon itself.^99,119,129,136,151^ Therefore, hydrogen bonding should be described as an indirectly supported co-mechanism unless before/after fiber FTIR, XPS, desorption, or competitive-binding data are available. In most nylon natural-dye systems, hydrogen bonding likely reinforces ionic attraction or hydrophobic partitioning instead of acting as the sole fixation mechanism.

### 6.3 Hydrophobic, van der Waals, and π-related interactions

Hydrophobic and van der Waals interactions are significant for colorants with long aromatic, conjugated or low-solubility structures. Nylon is polar compared with polyester, but its methylene segments can provide less polar domains that can associate with brazilein, curcumin, indigoids, crocin/crocetin, santalins, anthraquinones, lignin nanoparticles, prodigiosin-type pigments and fungal colorants.^34,36,39,69,79,98,112,113,119,149^ These dyes can sometimes act as disperse dyes and partition into the amorphous nylon in addition to interacting with the terminal amino sites. Nernst-type behavior for red sandalwood and nordamncanthal supports partitioning,^69,113^ and purpurin and nordamncanthal exhibited temperature dependence in diffusion into nylon.^112,113^ This advantage can be diminished by aggregation, as large oxidized catechol/chitosan products, lignin nanoparticles, oil-carried fungal pigments and large aggregates of dermocybin may deposit on the surface, or penetrate only partially.^33,149,166,167^ Therefore, aromaticity and hydrophobicity will promote dye uptake only when dye size and dispersion stability are suitable and the free volume within nylon is sufficient to permit internal diffusion.

### 6.4 Metal-mediated coordination and mordant bridges

Metal mordants can add depth to shades, shift color, and enhance fastness by creating dye-metal complexes and dye-metal-fiber bridges *via* dye hydroxyl/carbonyl and nylon amide, amino, or carboxyl sites.^22,40,75,78,79,97,129,135,139,151^ Fe salts frequently resulted in the highest darkening, such as *K*/*S* = 14.690 for kapok leaf extract with 10% owf FeSO_4_,^135^*f*_k_ = 21.61802 for *Areca catechu* with FeSO_4_ compared with 10.56554 for CuSO_4_ and 6.116 173 for alum,^129^ and higher *K*/*S* or light-fastness values in teak, mangrove, and cochineal systems.^40,78,139^ Mordanting can be understood as an enhancement of apparent dye uptake only if complexes are formed at or are strongly associated with the fiber. Abnormal cases hamper generalization; alum decreased the *K*/*S* of date palm fibrillium compared to unmordanted polyamide (3.72 compared to 4.05)^137^ and reduced the *K*/*S* of *Aspergillus pigment* from 2.5 to 2.0;^44^ nettle-pomegranate mordanted ratios increased the *K*/*S* of polyamide slightly from 11.30 to 12.27 at an optimized ratio of 90/30.^125^ Metal ions can be bound to nylon sites, can form precipitates in the dye bath, or can change the hue without giving rise to durable uptake. Mordant effectiveness should be judged on the basis of *K*/*S* in conjunction with washing/light fastness, metal-residue analysis and effluent burden rather than on the basis of *K*/*S* alone.

### 6.5 Covalent, enzymatic, and oxidative coupling

Covalent or oxidative coupling is most credible in enzymatic phenol systems. HRP/H_2_O_2_ oxidation of catechol, hydroquinone, and gallic acid can generate quinones and polymeric pigments, and the catechol-cadaverine model supported C–N-linked products by FTIR and ESI-MS.^32^ Chitosan-assisted gallic-acid coloration of nylon 5,10 gave the highest reported *K*/*S* in that system, at 4.5, showing that a bio-based amine-rich template can strengthen color build-up and antibacterial function.^33^ This does not prove universal covalent dye-nylon bonding. In the actual textile, oxidative products may form surface polymers, hydrogen-bonded networks, chitosan-mediated deposits, or covalent adducts simultaneously.^32,33^ Plasma activation, ozone treatment, and plant-mediated nanoparticle synthesis can also introduce reactive or polar groups,^20,41,58,173^ but without XPS/NMR/desorption evidence, they should be classified as reactive fixation candidates. Only systems with molecular-level identification of new fiber-bound bonds should be described as covalent dye-fiber fixation; most current nylon natural-dye reports support oxidative coating or coupling more strongly than confirmed covalent attachment.

### 6.6 Surface adsorption, coating, and internal diffusion

Nylon is often naturally dyed through a combination of surface adsorption, interfilament deposition, physical entrapment, and internal diffusion. Surface attachment of lignin nanoparticles, AgNP-extract complexes, alginate/TiO_2_ layers, fungal oil pigments, and other particulate or coating-type systems is supported by SEM/EDS and fluorescence microscopy and TGA.^35,149,166,173,174^ These systems can provide high *K*/*S* and functionality without necessarily showing molecular diffusion into nylon. Alternatively, temperature- and free-volume responsive uptake facilitates diffusion into amorphous polyamide regions. The crystallinity of PA56 was lower, and the fractional free volume was higher than those of PA6 and PA66, which is why PA56 is an easier to diffuse natural dye. Mangrove bark extract gave *K*/*S* values of around 1.66 at 30 °C and 5.38 at 90 °C, which was in line with the process of diffusion above the *T*_g_ of nylon.^40^ Coating may be more important for providing UV protection or antibacterial activity, whereas internal diffusion and strong fiber–site interaction are more important for washing durability. The abnormal decreases in *K*/*S* due to prolonged dyeing or high agitation, together with evidence of desorption after 75 min in ultrasonic olive-water dyeing,^43^ indicate that surface deposition and equilibrium retention can be unstable if there is no stronger fixation of the dye by the fiber.

### 6.7 Adsorption equilibrium and isotherm interpretation

The reported isotherms validate that the adsorption of natural dyestuffs on nylon is heterogeneous and system-specific. Langmuir behavior was reported for curcumin on polyamide, with *R*^2^ = 0.993, 0.981, and 0.991 at 60 °C, 80 °C, and 100 °C, respectively, and *q*_e,exp_ = 310 mg g^−1^, implying finite high-affinity sites.^104^ Lac dye exhibited Langmuir behavior on nylon with *Q* = 26.60 mg g^−1^ and *b* = 20.19 mL mg^−1^, which is higher than the affinity constant of wool or silk, but lower than the saturation capacity.^62^ Freundlich-type behavior was observed for saffron between 0.5% and 12% owf, showing heterogeneous adsorption instead of ideal monolayer coverage.^119^ Nernst partitioning for cochineal, madder, red sandalwood, purpurin, and nordamncanthal was found to follow a disperse-like solid-solution uptake mechanism.^61,69,81,112,113^ Turmeric yellow was more appropriately described by the Redlich–Peterson model, and the value of beta increased from 0.72 to 0.88 over the temperature range of 80 °C to 90 °C, indicating the presence of a mixed Langmuir–Freundlich character rather than a purely ideal surface.^109^ These models are meaningful only if the parameters are physically plausible and the equilibrium is verified. Reliance on *R*^2^ without residual plots, confidence intervals, dimensionless constants, and independent fiber analysis is insufficient, particularly for nylon, which is semicrystalline, contains finite end groups, amorphous diffusion domains, and has coating-prone surfaces.

### 6.8 Dyeing kinetics and rate-controlling steps

The kinetic data show that the process of nylon dyeing involves bath transport, boundary-layer diffusion, surface adsorption, internal diffusion, interaction between sites, and equilibrium saturation. Pseudo-second-order fits are commonly reported, such as for curcumin on polyamide (*R*^2^ = 0.995),^104^ lac on nylon (*R*^2^ = 0.9987–0.9998),^62^ and cochineal systems with high average fit quality.^61^ These fits should not be immediately assumed to be evidence of chemisorption because they may be caused by the approach to equilibrium, which is also concentration-dependent in finite-site, diffusion-resistant textile systems. This is evident in the case of lac dyeing. Increasing the temperature from 70 °C to 90 °C increased *k*_2_ from 1.265 to 3.036 × 10^−2^ g (mg^−1^ min^−1^), increased the initial adsorption rate from 7.20 to 14.22 mg (g^−1^ min^−1^), and reduced *t*_1/2_ from 3.313 to 1.522 min, yet equilibrium adsorption decreased from 23.87 to 21.65 mg g^−1^.^62^ Similarly, cochineal had a higher Chrastil rate constant at 90 °C than at 70 °C (0.3687 *versus* 0.2144 min^−1^), while *C*_∞_ decreased from 27.39 to 25.34 mg g^−1^.^169^ Red sandalwood showed faster apparent diffusion but lower equilibrium loading at higher temperature,^69^ whereas nordamncanthal showed both faster diffusion and higher standard affinity at 90 °C.^113^

Therefore, kinetic interpretation should be used to distinguish rate acceleration from equilibrium affinity rather than as a single model, as higher temperature may increase the transport while decreasing the final uptake in the case of exothermic or thermally sensitive dyes.

### 6.9 Thermodynamic interpretation

Thermodynamic parameters are informative only when calculated from true equilibrium data and dimensionally valid equilibrium constants. Negative Δ*G* or a positive standard affinity indicates favorable bath-to-fiber transfer, but it does not prove industrial feasibility, durability, or covalent bonding. Exothermic nylon systems include cochineal, with Δ*H* = −3771 cal mol^−1^ at pH 3 and −4768 cal mol^−1^ at pH 6,^61^ madder, where the partition coefficient decreased from 1453 at 40 °C to 876.79 at 80 °C,^81^ and red sandalwood, with Δ*H* = −24.689 kJ mol^−1^.^69^ These systems may dye faster at higher temperature while showing lower equilibrium affinity. Endothermic uptake was also reported for turmeric yellow, with Δ*H* = 71.34 kJ mol^−1^ and Δ*S* = 274 J mol^−1^ K^−1^ and an increase in affinity from 25.38 to 28.12 kJ mol^−1^ between 80 °C and 90 °C,^109^ as well as for purpurin and nordamncanthal, with positive Δ*H* values of 5.19 and 13.36 kJ mol^−1^.^112,113^ Endothermic uptake can support diffusion and partitioning but may be less attractive for cleaner processing if it requires higher thermal input. Curcumin illustrates a reporting problem because exothermic adsorption was reported with Δ*H* = −1.376 kJ mol^−1^ and Δ*S* = −29.05 J mol^−1^ K^−1^, while the stated positive Δ*G* values conflict with simple spontaneity claims unless the equilibrium constant is carefully defined.^104^ Future studies should report dimensionless equilibrium constants, uncertainty, residual-bath concentrations, and equilibrium verification before assigning spontaneity.

### 6.10 Strength and limitations of mechanistic evidence

Mechanistic evidence from the studies on nylon natural dyes can be classified into three levels. Level 1 comprises proposed mechanisms based on functional-group reasoning, schematic ionic and mordant–bridge diagrams, and *K*/*S* trends, but these mechanisms are not directly validated. Many studies are still only qualitative and estimate the level of interaction based on acidic pH response, mordant-induced shade change, or improvement of fastness.^60,92,129,134,153^ To distinguish different forms of indirect evidence more clearly, Level 2 is divided into two subcategories. Level 2A includes fiber-associated spectroscopic or morphological support, such as FTIR shifts, deposition observed by SEM/EDS, fluorescence microscopy, and changes in zeta potential. These techniques can support dye association, surface deposition, or changes in the local chemical environment, but do not by themselves identify a specific molecular bond. Level 2B includes quantitative evidence from adsorption isotherms, kinetic modeling, exhaustion data, partitioning, diffusion, or thermodynamic analysis, which can provide stronger support for uptake and transport mechanisms but likewise does not independently prove a particular dye–fiber bond.^22,41,42,59,62,104,119,147,149,163,173^ Level 3 is molecular-level validation using XPS, NMR/MS of bound species, desorption and re-dyeing studies, competitive binding, metal-location analysis, isotopic or chromatographic tracking, and experimentally validated molecular simulation. Selected studies provide Level 3 evidence for specific interactions, such as HRP model-compound evidence for C–N coupling,^32^ XPS-supported *Monascus*-polyamide analysis,^163^ and alizarin–ZnO binding supported by UV-vis, FTIR, NMR, XPS, TGA, TEM and DFT.^71^ In contrast, the PA56 study based on crystallinity/free-volume analysis and molecular simulation^70^ provides strong Level 2B evidence for diffusion and transport behavior rather than direct proof of a specific dye-fiber bond. These studies reinforce the mechanistic interpretation; however, only the nylon fiber–bound bond in the dyed nylon can be claimed when directly supported by evidence. Accordingly, pH-dependent *K*/*S*, FTIR shifts, SEM/EDS observations, or adsorption-model fits should be interpreted as supporting evidence rather than proof of ionic, hydrogen, coordination, or covalent bonding unless the proposed interaction is directly validated at the dyed-fiber or relevant interface.

Overall, nylon natural dyeing can be termed as a mixed-fixation process rather than a definite single bonding pattern. The mechanism of uptake involves the acid-assisted ionic attraction of the colorant to the –NH_3_^+^ groups; phenolic-, carbonyl-, and methoxy-containing colorants are stabilized by hydrogen bonding and dipole interactions; uptake of aromatic and anthraquinone colorants is promoted by hydrophobic and π-related partitioning into the amorphous nylon; and when metals are fiber-associated, the resulting complexes are stabilized by the coordination bridges provided by mordants. Therefore, these interactions should be regarded as proposed or supported mechanisms unless they have been directly validated at the dyed-fiber/interface level. Ionic attraction is generally strong and process-controllable, but it is pH- and dye-charge-dependent. Hydrogen bonding is widespread but provides only moderate evidence rather than hard proof. Hydrophobic partitioning may be high in the case of small planar dyes and low in the case of aggregated or particulate dyes. Metal coordination can be very useful for shade deepening and light/wash fastness, particularly in the case of metal salts of Fe and Cu, but it can be environmentally and analytically problematic when no data on metal leaching are available. Biomordants and tannin-rich auxiliaries, which enhance *K*/*S* and fastness, are cleaner alternatives but do not necessarily outperform metals. Their usefulness is not with respect to a removable surface layer but in their ability to concentrate colorants, facilitate extraction, and form a durable interfacial network. The best future design approach is an evidence-based design of a low-metal or metal-free system that incorporates controlled pH, optimized diffusion, verified fiber-level bonding, and fastness after repeated laundering. The reported adsorption, kinetic, thermodynamic, and mechanistic evidence for natural-dye uptake by nylon is summarized in Table 3.

**Table 3:** Adsorption, kinetic, thermodynamic, and mechanistic evidence reported for natural-dye uptake by nylon

Dye system/class	Nylon type	Key adsorption, kinetic, or thermodynamic evidence	Techniques used to support the proposed mechanism	Proposed mechanism	Evidence level	Main limitation	Ref.
HRP-oxidized catechol, hydroquinone, and gallic acid; chitosan-assisted phenolic pigments	Nylon 5,10	No isotherm, kinetic, or equilibrium constants reported; mild enzymatic coloration	FTIR- and MS-based characterization of oxidative products/model reactions, SEM, *K*/*S*, colorimetric and fastness evidence	Oxidative quinone/polyphenol formation, hydrogen bonding, and possible C–N coupling through amine sites	Ref. 32: level 3 for model-reaction C–N evidence, but level 2A for actual fiber attachment:^33^ level 2A	Model reaction supports possible coupling but does not prove universal covalent fiber bonding	32 and 33
Rice-straw phenolics/flavonoids	Nylon 6	No formal adsorption or thermodynamic analysis; plasma improved uptake and function	LC-MS/MS and UV-vis for extract characterization; FTIR, SEM, XRD, TGA/DSC and colorimetric analysis of the treated system	pH-assisted ionic attraction, hydrogen bonding, plasma-created polar sites, and surface activation	Level 2A	Surface activation was not separated from true molecular fixation	20
Annatto/norbixinate carotenoids	Polyamide-rich and PA6.6 fabrics	High exhaustion under acidic conditions; no formal thermodynamic analysis	UV-vis/exhaustion, FTIR, SEM and colorimetry; *K*/*S*, CIELAB and fastness comparisons; concentration-dependent exhaustion and *K*/*S*	Carboxylate/phenolic dye groups interacting with protonated nylon sites; protein/soy layer may entrap dye	Ref. 57: level 2A:^91^ level 1:^121^ level 2B	Biopretreatment, coating, and fiber diffusion were not separated	57, 91 and 121
Luteolin/weld flavonoids	PA56 and recycled PA6.6	Mordanting/finishing improved thermal stability and light fastness; no adsorption constants	FTIR, TGA, colorimetric, fastness and functional analyses; FTIR/chemical characterization and surface/morphological evidence associated with finishing; mainly *K*/*S*, CIELAB and process-response evidence	Hydrogen bonding before mordanting; Al/Fe-mediated dye-metal-fiber bridges after mordanting	Ref. 97: level 2A:^35^ level 2A:^116^ level 1	Metal location, leaching, and true fiber-bound fraction were not quantified	35, 97 and 116
Curcumin/turmeric	Knitted polyamide	Langmuir fit, *R*^2^ = 0.981–0.993; *q*_e_,_exp_ = 310 mg g^−1^; pseudo-second-order, *R*^2^ = 0.995; exothermic Δ*H* = −1.376 kJ mol^−1^	Adsorption-isotherm fitting, kinetic modeling, thermodynamic analysis and color-strength measurements	Ionic/polar affinity, hydrogen bonding, and hydrophobic curcumin association	Level 2B	Positive Δ*G* values complicate spontaneity claims; poor light/wash durability remains limiting	104
Saffron/crocin-rich dye	Nylon 6.6	Freundlich-type adsorption over 0.5–12% owf; no Δ*G*/Δ*H*/Δ*S* reported	Dye characterization, exhaustion measurements, *K*/*S* and Freundlich-type adsorption analysis	Heterogeneous adsorption and acid-assisted dye affinity	Level 2B	Based mainly on uptake curves without direct dyed-fiber spectroscopy	119
Cochineal/carminic acid	Polyamide 66	Nernst behavior at pH 3 and 6; pseudo-second-order, average *R*^2^ ≈ 0.990; exothermic Δ*H* = −3771 to −4768 cal mol^−1^	Equilibrium uptake, nernst partition analysis, kinetic modeling and thermodynamic calculations	Carboxylated dye attraction to protonated amino sites, with non-electrostatic contributions at higher pH	Level 2B	Kinetic fit does not identify the exact bond; fiber-level XPS/metal mapping was absent	61
Madder anthraquinones	Nylon yarn/fabric	Nernst partitioning over 0.6–3.0 g L^−1^; equilibrium time shortened from 50–55 to 40–45 min between 40 and 80 °C; partition coefficient decreased from 1453 to 876.79	Equilibrium uptake, concentration-dependent partitioning, dyeing-rate analysis and thermodynamic calculations	Electrostatic attraction, hydrogen bonding, and hydrophobic anthraquinone partitioning	Level 2B	Individual anthraquinone contributions and aggregation state remain unresolved	81
Lac dye/laccaic acids	Nylon	Langmuir *Q* = 26.60 mg g^−1^ and *b* = 20.19 mL mg^−1^; pseudo-second-order, *R*^2^ = 0.9987–0.9998; *t*_1/2_ decreased from 3.313 to 1.522 min between 70 and 90 °C; *E*_a_ = 45.31 kJ mol^−1^	Adsorption-isotherm analysis, kinetic modeling, equilibrium uptake and activation-energy calculation	Ion–ion interaction between dye carboxyl groups and protonated nylon sites	Level 2B	Chemisorption was inferred mainly from model fitting; equilibrium uptake decreased at higher temperature	62
Turmeric yellow	Nylon	Redlich–Peterson best fit; *β* increased from 0.72 to 0.88 between 80 °C and 90 °C; endothermic Δ*H* = 71.34 kJ mol^−1^; Δ*S* = 274 J mol^−1^ K^−1^	Adsorption-model comparison and thermodynamic analysis	Mixed finite-site and heterogeneous adsorption with diffusion contribution	Level 2B	Model selection depends on fit statistics; fiber-level proof is limited	109
Red sandalwood/santalin	Nylon fibers	Nernst slope decreased from 1127.883 to 589.951 between 70 °C and 90 °C; etters diffusion increased from 5.412 to 8.123; exothermic Δ*H* = −24.689 kJ mol^−1^	Nernst partition analysis, diffusion measurements and thermodynamic calculations	Hydrophobic partitioning and internal diffusion	Level 2B	Higher temperature accelerated diffusion but lowered equilibrium loading	69
Purpurin and nordamncanthal anthraquinones	Nylon	Nernst/linear partitioning; purpurin *t*_1/2_ decreased from 60.1 to 9.0 min; nordamncanthal *D* ≈ 1.4–1.6 × 10^−8^ cm^2^ s^−1^; Δ*H* = 5.19–13.36 kJ mol^−1^	Uptake/partition and diffusion analysis; for chemically isolated anthraquinones, molecular identification was supported by chromatographic/spectroscopic methods together with equilibrium analysis	Disperse-like hydrophobic partitioning into nylon	Level 2B	Thermal stability limits processing; high uptake does not necessarily indicate durable fastness	112 and 113
Fungal anthraquinones: emodin and dermocybin	Polyamide knitted fabric	Uptake measured but not modeled; acidic baths gave 97–100% emodin uptake	Pigment purity/identity analysis, residual-bath uptake measurements, CIELAB and standardized fastness testing	Hydrogen bonding, π-related aggregation, and possible metal-anthraquinone coordination with amide sites	Level 2B	High uptake may include aggregation or surface adsorption; depth and fastness data are needed	79
*Aspergillus turcosus* yellow pigment	PA6	No formal adsorption, kinetic, or thermodynamic constants reported	Pigment FTIR, NMR and UV-vis; dyed-fiber FTIR/XRD, *K*/*S*, CIELAB and functional testing	Acidic ionic attraction, hydrogen bonding, and hydrophobic/aromatic interactions	Level 2A	Alum reduced *K*/*S*; adsorption constants and penetration depth were not reported; proposed covalent-type assignment remains unconfirmed	44
Monascus pigments	Silk/polyamide two-component fabric	No formal adsorption, kinetic, or thermodynamic constants reported; pigment distribution between fiber components was experimentally examined	Optical/cross-sectional microscopy, SEM, separated-fiber analysis, UV-vis and XPS	Hydrophobic interaction, hydrogen bonding and van der Waals association at the polyamide/pigment interface	Level 3 for the investigated fiber/interface evidence	The individual contributions of the proposed interactions were not quantitatively separated	163
Alizarin-ZnO hybrid colorant	PA4,10	No nylon adsorption isotherm or kinetic analysis; alizarin–ZnO binding was characterized before incorporation into the polyamide melt	UV-vis, FTIR, ^1^H NMR, ^13^C CP-MAS NMR, XPS, TGA, TEM and DFT	Bidentate catechol-type binding of alizarin to ZnO, followed by physical incorporation of the stabilized colorant into polyamide	Level 3 for the alizarin-ZnO interface	Strong molecular validation of alizarin–ZnO binding does not prove a covalent alizarin-polyamide bond	71
Natural-dye diffusion comparison among polyamides	PA56, PA6 and PA66	Comparative dye uptake associated with differences in crystallinity and fractional free volume; PA56 showed easier dye diffusion than PA6/PA66	Crystallinity/free-volume characterization, comparative dye uptake and molecular simulation	Free-volume-controlled diffusion into amorphous polyamide regions	Level 2B	Strong evidence for transport/diffusion, but not direct identification of a specific dye-polyamide bond	70

## 7 Coloration, fastness, functionality, and durability

### 7.1 Color strength, exhaustion, and fixation

Color strength in naturally dyed nylon is best interpreted as a system response rather than a direct property of a dye source. Across exhaust dyeing studies, the most reliable single-wavelength *K*/*S* values fall within the low-to-moderate range of about 2–10, including HRP-polymerized catechol/hydroquinone on nylon 5,10 and nylon 6 (*K*/*S* of 2.4–3.4),^32^ gallic acid/chitosan on nylon 5,10 (*K*/*S* of up to 4.5),^33^ rice-straw extract on nylon 6 (*K*/*S* of 6.6, increasing to 10.7 after plasma),^20^ lignin nanoparticles on PA6.6 (*K*/*S* of up to 9.1),^149^ and microbial prodigiosin on silk/polyamide (3.9).^36^ Higher but still comparable *K*/*S* values were reported for pomegranate peel after laser pretreatment (15.2),^45^ saffron stamen before saturation (about 16.5),^115^ electron-beam-assisted curcumin on nylon 6 (about 18.1),^105^ olive-leaf extract on PA6 (18.60–20.09),^132^ and *Terminalia catappa* (21.94).^96^ Abnormal or metric-specific values should be considered separately, because annatto studies reported very high *K*/*S* values of 167.68–251.24,^57,91^ and supercritical CO_2_ alizarin gave summed *K*/*S* values of 52.9–102.3 rather than a conventional single-wavelength value.^68^ These results are valid within their own methods but should not be used as a normalized ranking.

The strongest chromophores were not necessarily the best-performing classes. Anthraquinones, indigoids, tannins/polyphenols, flavonoids, curcuminoids, and modified betalain/azo structures were found to be effective when their molecular size, ionization, diffusion, and complexation were similar to those of polyamide substrate. Nylon grade was an important factor, as nylon 6 readily accepted HRP pigments more than nylon 5,10 (ref. 32) and PA56 exhibited greater natural dye uptake than PA6 and PA66 due to its lower crystallinity and higher free volume.^70^ Mordants generally enhanced *K*/*S* and fixation, particularly with Fe, Cu, Sn, Al, tannic acid, myrobalan, pomegranate peel and chitosan,^22,83,131,133–135^ but not always, as alum reduced the *K*/*S* of fungal pigments on PA6 (ref. 44) and some biomordants increased fastness but decreased the shade depth.^137^ Concentration build-up usually reached a saturation point, as saffron stamen uptake approached 10 g L^−1^.^115^ Most importantly, high exhaustion did not ensure durability in service, since exhaustion of the compound by polyamide reached around 98–99%, whereas photostability was still limited.^39^

### 7.2 CIELAB behavior and shade diversity

The results of CIELAB analysis indicate that natural dyes can offer a wide but uneven shade diversity on nylon. Most treatments reduce L*, producing darker shades as concentration, mordanting, plasma, laser, microwave, or surface activation increases. Rice-straw plasma treatment decreased the L* of nylon 6 from 60.92 to 43.02 and increased a*/b* values from 5.27/18.87 to 10.45/24.49.^20^ Similar results were seen for pomegranate-peel laser pre-treatment, which decreased L* and boosted *K*/*S*.^45^ Positive a* and b* values are dominant in red-orange and yellow-brown systems, as Brazilwood produced reddish-orange nylon with L* of 45.60, a* of 40.33, and b* of 51.70, whereas Fe and Cu changed the coloration to darker brown or reddish-brown shades.^98^ Annatto produced orange hues with favorable positive a* and b* values, which were enhanced by acidic dyeing, while alkaline soy milk resulted in better levelness but lower color intensity.^57^

The yellow systems, including luteolin, curcumin, saffron, weld, rice straw, pomegranate peel, and *Aspergillus* pigment, typically had positive b* values, whereas the indigoids had negative b* values and blue or greenish-blue hues.^80^ Prodigiosin and emodin derivatives broadened the color spectrum to red–purple, violet, and pH-dependent colors.^36,79,110^ A serious drawback is the concentration of shades within families of yellow, brown, orange, red-brown, olive, blue, and purple. Bright green, stable black, and vivid color shades are rare unless blended, chemically modified natural chromophores, metal coordinated chromophores, or microbial pigments are used. The use of pH is beneficial for shade control but has been found to introduce reproducibility risks, as ionization of the same extract could change the shade from brilliant to dull, or from red to blue.^44,57,107,108,114^

### 7.3 Levelness, reproducibility, and extract variability

Levelness is still poorly documented. Good examples include *Monascus* dyeings with *K*/*S* standard deviations of 0.08 and 0.07 (ref. 163) and *Aspergillus* pigment dyeing with RUI = 0.04 at 3% shade.^44^ In contrast, *in situ* chitosan-assisted phenolic polymerization resulted in uneven, surface-rich coloring^33^ and acidic annatto dyeing resulted in a very high *K*/*S* value but a high Δ*E* value, whereas alkaline soy milk reduced color depth and improved levelness.^57^ Reproducibility is also affected by plant age, bark origin, harvest season, waste composition, extraction pH, solvent, and storage. Many papers still report only a single *K*/*S* or fastness value, without standard deviations, replicate runs, or extract fingerprints. Because fabric construction also varies from woven PA6 and PA6.6 to PA56, nylon/PU, and silk/polyamide blends, cross-study ranking is unsafe unless future work reports extract yield, UV-vis/HPLC fingerprints, replicate *K*/*S*, RUI or color-mapping data, and post-wash Δ*E*.

Accordingly, absolute *K*/*S*, fastness, antimicrobial, and UPF values were interpreted with caution when substrate characteristics, processing conditions, or test methods differed across studies. Cross-study comparisons were therefore used primarily to identify general trends and relative improvements within individual studies, while more direct numerical comparisons were made only where the nylon substrate, processing conditions, measurement methods, and test endpoints were sufficiently comparable. Antimicrobial inhibition-zone data were considered separately from percentage-reduction results, and UPF values were interpreted in relation to the corresponding undyed control and fabric construction.

### 7.4 Washing and perspiration fastness

Washing fastness is generally better than light fastness, but color change must be separated from staining. Many naturally dyed nylons show staining grades of 4–5 or 5 even when shade fading is only 2–3 or 3. Mordant-free dyeing can be robust when affinity is high, as curcumin on polyamide showed a washing color-change grade and staining grade of 5,^39^ hawthorn-dyed PA6 showed a color-change grade of 4–5 and staining of 5,^122^ halochromic dyes on PA6.6 reached grades of 4–5 to 5,^107^ and eucalyptus-residue dyed nylon gave a color-change of 4.5–5 with excellent staining.^153^ However, direct dyeing may fail because mangrove bark showed a color change of only 1–2 without mordant despite staining of 4–5,^40^ and Danta bark on nylon 6,6 improved from grade 2 to 4 after copper sulfate mordanting.^151^

Metal salts, especially Cu, Fe, Sn, and Al, are frequently used to enhance washing durability,^22,133^ but this conflicts with the objective of cleaner processing. Biomordants are attractive but not necessarily superior, as soy milk improved annatto washing fastness but wet-rubbing staining remained weak under alkaline conditions;^57^ pomegranate and myrobalan improved annatto fastness;^83^ and tannic acid improved curcumin print washing fastness rather than light stability.^42^ Intensified processing can also enhance penetration and fixation, as olive-water dyeing using ultrasonic treatment increased wash fastness from 3–4 to 4 (ref. 43) and microwave treatment increased exhaustion and wash fastness.^92^ There is less perspiration data, but it plays an important role in apparel. Acidic and alkaline perspiration fastness was often acceptable at 4–5,^114,122^ while PA6.6 dyed with silkworm excrement was rated at 2–3 to 3–4 (ref. 152) and luteolin-dyed PA56 showed a decrease to grade 1 in alkaline perspiration staining before metal coordination.^97^

### 7.5 Rubbing and light fastness

Dry rubbing is usually better than wet rubbing because water mobilizes surface deposits, swelling agents, protein binders, or pigment particles. FeSO_4_-mordanted nylon 6 reached dry rubbing up to 5, but wet rubbing ranged from 1 to 3–4 depending on concentration.^134^*Terminalia catappa* showed dry rubbing of 4–5 but wet rubbing of 3–4,^96^ sesame-dyed nylon retained acceptable washing fastness but weak wet-rubbing and light fastness,^77^ and soy-milk-assisted annatto at alkaline pH gave weak wet-rubbing staining of 2/3.^57^ Thus, high *K*/*S* may indicate surface-rich coloration rather than fully internalized dye, especially for pigment coatings, *in situ*-polymerized colorants, and biomordant films.

Light fastness remains the major durability barrier. Weak values of 1–3 were reported for sesame,^77^ mangrove bark,^40^ catechu and mulberry at an industrial scale,^76^ spent coffee grounds,^140^ sea buckthorn leaf,^72^ tomato-waste carotenoids,^146^ and curcumin or curcumin prints (3 to 3–4).^39,42^ Better photostability required stabilizing chemistry, with bamboo leaf treated by SnCl_2_ or FeSO_4_ reaching a grade of 7,^133^*Prangos* with pomegranate or *Terminalia* reaching 8 or 7–8,^131^ walnut husk biomordant reaching 8,^117^*Scrophularia*/AgNP-treated PA6 reaching 8,^173^ and weld with sodium alginate/TiO_2_ improving to 5.^35^ Therefore, indigoid, anthraquinone, tannin-rich, and metal/biomordant-stabilized systems are more suitable for light-exposed nylon than carotenoid and curcuminoid systems unless photostabilization is added.

Chemically induced shade change should be differentiated from dye loss, as well as from photofading. Some natural colorants are sensitive to changes in their chemical environment; for example, luteolin showed weak alkaline-perspiration performance before metal coordination,^97^ selected bacterial pigments showed pigment-dependent instability under alkaline and detergent exposure,^159^ and emodin exhibited a large washing-induced color difference (Δ*E* 9.80) despite high polyamide uptake.^167^ These observations show that colour stability can change with laundering or perspiration even if the colour affinity is initially high. However, atmospheric-pollutant exposure and oxidative-detergent stability have rarely been evaluated in the nylon-specific literature. Therefore, future studies should quantify CIELAB/Δ*E* changes after alkaline perspiration, oxidative or detergent exposure, and controlled atmospheric ageing, and distinguish chemical hue change from dye desorption or staining. This represents an important gap in consumer-relevant durability assessment.

### 7.6 Antibacterial and antimicrobial properties

Antimicrobial studies mainly used *Escherichia coli* and *Staphylococcus aureus*, with some adding *Bacillus subtilis*, *Pseudomonas* spp., *Klebsiella pneumoniae*, *Candida albicans*, or fungi. Qualitative inhibition zones and quantitative reduction values should not be treated as equivalent. Gallic acid/chitosan produced 15.1 mm and 15.03 mm zones against *E. coli* and *S. aureus*, decreasing to 11.4 and 10.9 mm, respectively, after 10 washes.^33^*Aspergillus* pigment on PA6 gave 27/30 mm zones against *B. subtilis*/*E. coli*, and alum-premordanted fabric retained zones of 24/25 mm after 20 washes.^44^ Quantitative reductions were often very high, including more than 99.9% *S. aureus* and 99.2% *E. coli* reductions in spent-coffee-ground-dyed nylon,^140^ 99.8–99.9% reductions for sea-buckthorn dyed nylon/PU,^72^ 99.7% *E. coli* and 99.9% *S. aureus* inhibition for *Ipomoea batatas* leaf-dyed nylon,^138^ 99.9% reduction against *S. aureus* and *K. pneumoniae* for berberine-treated nylon 66,^66^ and an initial reduction of 100% for *Scrophularia*/AgNP-treated PA6.^173^ The main limitation is attribution. Bioactivity may arise from chitosan, Ag ions/AgNPs, copper salts, berberine cations, or residual phenolics rather than from the chromophore alone. *Scrophularia*/AgNP samples retained 79–88% microbial reduction after 10 washes, but Ag release was not quantified.^173^ Therefore, durable antimicrobial claims require post-wash microbial-reduction data and leaching data.

### 7.7 UV protection and antioxidant performance

UV protection is one of the best functional outcomes, as phenolic, flavonoid, tannin, anthraquinone, curcuminoid, lignin, chlorophyll, and microbial pigment structures absorb UVA/UVB radiation. Dyeing often could turn poor-to-moderate nylon protection into excellent UPF performance. Rice-straw extract increased UPF from 2.3 to 72.6 for nylon 6, and plasma-dyed fabric reached 79.9 while retaining 69.0 after 20 washes.^20^ Babool bark raised UPF from 12.1 to 87.622;^59^ spent coffee grounds raised the UPF of nylon 6,6 from 15.77 to 177.53;^140^ the UPF of sea buckthorn leaf on nylon/PU was higher than 400 and reached up to 764.1 under acidic conditions;^72^ and luteolin on PA56 increased UPF from 34 to 205 and remained at about 155 after 10 washes with Al^3+^.^97^ The highest absolute UPF values were obtained for industrial polyamide interlock fabrics, with catechu, pomegranate peel, and mulberry leaf reaching UPFs of 2000, and madder reaching 1818, while the undyed fabric already had an UPF of 1324.51, which shows that the dense fabric construction made a significant contribution to these extreme values.^76^ Accordingly, these very high absolute UPF values should not be attributed to the natural dyes alone. The contribution of coloration is more appropriately evaluated by comparing each dyed sample with its corresponding undyed fabric of the same construction. Therefore, areal density, thickness, weave or knit structure, yarn specification, and thread or stitch density should be reported alongside UPF data, because differences in fabric cover can substantially influence UV transmission independently of dye chemistry.

The most effective dye-induced increase was detected for *Terminalia catappa*, which increased the UPF of nylon from 26.39 to 1995 and decreased UVA/UVB transmittance to 0.03%.^96^ This paired dyed-versus-undyed comparison provides clearer evidence of the contribution of coloration than the absolute UPF of the dyed fabric alone. The strongest laundering evidence was reported for the baicalin, which maintained a UPF of above 30 after 5–30 cycles and 26.12 after 50 cycles.^47^ Antioxidant data are less commonly reported but remain consistent with aromatic phenolic chemistry, as rice-straw nylon retained DPPH activity of 34.9% after 20 washes,^20^ while luteolin-dyed PA56 retained more than 60% ABTS activity after 10 washing cycles,^97^ and the antioxidant activity of the FeSO_4_-mordanted kapok leaf nylon was 94.42%.^135^ Shade depth can aid in UV protection, but it is more attributed to dye fixation than to *K*/*S* alone.

### 7.8 Smart and additional functions

Smart functionality in naturally dyed nylon remains limited, and the clearest evidence comes from pH-responsive systems. Curcumin, with bromothymol blue used as a comparator, on a cotton/polyamide 6 medical dressing showed visible halochromism across pH 5.5–8.5, matching the shift from healthy skin to alkaline wound conditions, with the clearest curcumin response reaching ΔE* ≈ 29 at pH 8.5.^106^ This evidence is comparatively strong because HaCaT viability remained comparable with untreated controls, hemolysis was below 2%, and no dye was detected in the Franz-cell receptor phase after 26 h.^106^ Curcumin stayed superficial, decreasing from 0.64 ± 0.12 µg cm^−1^ in the stratum corneum at pH 5.5 to 0.46 ± 0.05 µg cm^−1^ at pH 8, with no dermal detection.^106^ However, the blended substrate limits direct generalization to pure nylon.

Other responsive systems are promising but less validated. Japanese knotweed emodin showed visible pH 1–14 color transitions linked to a 436-to-523 nm spectral shift, but reversibility and post-wash response were not quantified.^110^ A polyamide pH sensor retained its spectral absorbance response after one anionic wash, one non-ionic wash, and one dry-cleaning cycle, although full reversibility occurred at pH 4.0 rather than pH 6.5.^108^ Prodigiosin pigments and *Aspergillus turcosus* pigment also showed pH-responsive behavior, the latter with antibacterial, antioxidant, and UV-protective effects.^44,160^ Still, repeated sensing, perspiration interference, storage stability, and leaching remain insufficiently tested.

Additional functions include fluorescence, thermal stability, char formation, wettability, and release behavior. Pomegranate-peel polyphenols gave fluorescence and UPF of up to 192.73 after 12 W laser pretreatment,^45^ while rice straw extract improved wettability from 30 s to 7 s and moisture regain from 5.1% to 10.2%, with thermal improvement inferred from TGA/DSC rather than confirmed flame testing.^20^ Lignin nanoparticles increased PA6.6 char residue from 3.55% to 15.93%, although flame resistance was inferred rather than confirmed by standard flame testing,^149^ and alizarin-ZnO nanoparticles improved thermal stability by more than 100 °C while reducing UV-induced ΔE* from 15.74 to 6.06.^71^ Anti-odor performance remains indirectly supported because most studies report antibacterial activity rather than odor-molecule or wearer trials.^19,33,36,42,87^ Gall oak/madder dyeing improved polyamide/elastane wettability, reducing the contact angle from 90.97° ± 3.85° to 30.80°.^73^ Curcumin release reached 2.79 mg g^−1^ in 100% ethanol after 12 h, but its body-fluid relevance remains unverified.^104^

### 7.9 Functional durability and service-life performance

The most reliable evidence of durability comes from repeated use tests rather than from a single fastness test. Rice-straw plasma-dyed nylon 6 exhibited a UPF of 69.0 with antioxidant activity of 34.9% after 20 washings.^20^ Baicalin-dyed nylon retained significant UV protection after 50 cycles, while the *K*/*S* value decreased from 4.1 to 2.0 at 3% owf.^47^ Luteolin-dyed PA56 exhibited a good balance of durability with Al^3+^ coordination, having a UPF of around 155, ABTS scavenging activity above 60%, and antibacterial activity against *S. aureus* of approximately 75% after 10 washes.^97^ The inhibition zones of the chitosan-templated gallic acid pigments were still detected but were reduced after 10 cycles.^33^*Scrophularia*/AgNP-treated PA6 retained 79–88% antibacterial reduction after 10 cycles,^173^ and walnut-dyed PA6.6 retained stronger antimicrobial activity after 20 cycles when mordanted, especially with Fe, Cu, or alum.^145^

Durability depends on the functional mechanism. Absorbed aromatic chromophores provide UV protection that can withstand washing, while antibacterial activity is more susceptible to leaching, loss of surface-active groups, or depletion of metal ions. Rubbing, perspiration, flexing, storage and light ageing are not generally linked to functional retention, but are intrinsic to the service life of real nylon apparel. This is a serious gap, as many systems exhibit good washing fastness but poor light, wet-rubbing, perspiration, or functional durability under repeated use. Smart textiles need to demonstrate cyclic pH responsiveness after laundering, antimicrobial textiles need to retain their log or percentage reduction after washing, abrasion and perspiration, and UV textiles need to retain their UPF after washing and accelerated light exposure. Leaching and hand-feel assessment are also required for surface-rich systems containing chitosan, soy protein, alginate, oil-carried fungal pigments or AgNPs.

Therefore, future durability studies should move beyond testing individual stresses separately and adopt application-specific, sequential, combined-cycle protocols that more closely represent service conditions. For example, performance may be assessed initially, after repeated laundering, such as 10 washing cycles, and again after subsequent accelerated light ageing or abrasion; for sportswear or skin-contact textiles, laundering may be followed by perspiration and rubbing cycles. *K*/*S*, Δ*E*, and the relevant functional property, such as UPF, antibacterial or antioxidant activity, or pH response, should be measured at predefined stages to determine cumulative performance loss rather than resistance to a single stress alone.

Overall, the most balanced candidates are not necessarily those with the highest *K*/*S*. A practical benchmark for manuscript-level comparison should combine moderate-to-high *K*/*S* with washing fastness of at least 4, wet rubbing of at least 4, light fastness of above 4–5, and verified functional retention after laundering. Based on current evidence, rice-straw extract with plasma activation, luteolin with controlled Al^3+^ coordination, baicalin for durable UV protection, *Scrophularia*/AgNP and walnut/mordant systems for antimicrobial durability, and selected tannin/biomordant systems are more convincing than high-*K*/*S* but light-unstable carotenoid, curcumin-only, coffee-waste, or pigment-coating systems. Future studies should report *K*/*S* retention, Δ*E* after repeated washing, wet rubbing, acidic and alkaline perspiration, light ageing, retained UPF or antimicrobial reduction, and leached colorants or metal/nanoparticle release in one test matrix. Representative coloration, fastness, functional-performance, and durability benchmarks for naturally dyed nylon are presented in Table 4.

**Table 4:** Representative performance benchmarks for naturally dyed nylon

Dye/colorant class	Nylon type	Processing route	*K*/*S* or color outcome	Washing fastness	Light fastness	Functional property	Durability evidence	Key trade-off	Ref.
Phenolic/quinonoid HRP pigments	Nylon 5,10; nylon 6	HRP/H_2_O_2_, mordant-free	*K*/*S* = 2.4–3.4	Acceptable in selected cases	Poor-limited	Zones 8.6/7.7 mm *vs. E. coli*/*S. aureus*	No bioactivity wash test	Mild route but low depth and weak photostability	32
Gallic acid/catechol + chitosan	Nylon 5,10	*Ex situ* or *in situ* templating	GA/CS *K*/*S* = 4.5; CA/CS = 3.0	Acceptable	Poor	15.1/15.03 mm zones	After 10 washes, 11.4/10.9 mm	Chitosan drives high activity; *in situ* unevenness	33
Rice-straw phenolics/flavonoids	Nylon 6	Mordant-free; plasma activation	*K*/*S* = 6.6 to 10.7	Good	Good reported	UPF = 72.6–79.9; DPPH = 39.6%	After 20 washes, UPF 69.0 and DPPH = 34.9%	Strong durable function; outdoor ageing absent	20
Annatto carotenoid	PA/elastane 95/5	pH control; soy milk	*K*/*S* = 30.41–191.40; uniform = 40.30	Grade = 5 in soy systems	Not reported	Orange shade	Alkaline soy gave grade 4 after 5 washes	Very high acidic depth but poorer levelness	57
Weld flavonoids + TiO_2_/alginate	Recycled nylon 6,6	Post-finishing	Yellow; b* up to 17.7	Not fully reported	Up to 5	UPF up to 311	No laundering durability	UV/light gain; nanoparticle release untested	35
Microbial prodigiosin	Silk/PA66	pH-controlled nano pigment	*K*/*S* = 3.9; purple–red	Good	Limited	77.8% antibacterial; UPF = 15.1	No repeated laundering	Functional color but weak durability evidence	36
Luteolin flavonoid	PA56	Direct dyeing; Al/Fe coordination	Uptake = about 98%; yellow–brown	4 or above after coordination	Improved	UPF = 205; ABTS = 99%; 70%/95% antibacterial activities	After 10 washes, UPF ∼155 and ABTS above 60%	Excellent function but uses metal coordination	97
Olive-leaf phenolics	PA6	Exhaust dyeing; optional Cu	*K*/*S* = 18.60–20.09	Excellent in many samples	Weaker than wash	UPF = 45; Cu gave 98.10% *C. albicans* reduction	Functional wash durability absent	High *K*/*S*; Cu adds metal burden	132
Babool bark tannins	Nylon fabric	Mordant-free exhaust	*K*/*S* = 7.3 at pH 2	Good-excellent	4	UPF = 87.622; extract zones = 22/18 mm	No functional wash durability	Good UV and wet fastness; moderate light fastness	59
Spent coffee melanoidins	Nylon 6,6	Mordant-free; cassava tannin option	*K*/*S* = 3.18–3.32	Good-excellent	1/2–2	More than 99.9% *S. aureus* and 99.2–99.6% *E. coli* reductions; UPF = 177.53	No functional wash test	Excellent initial function but poor light fastness	140
Sea buckthorn leaf phenolics	Nylon/PU tricot	pH and concentration control	Δ*E* up to 47.12	Good-excellent	1–2 to 2	99.8–99.9% microbial reductions; UPF above 400 to 764.1	No retention after washes	Outstanding UPF but very weak light fastness	72
Bio-indigo/indigoid	Nylon	Bio or reduction-oxidation dyeing	*K*/*S* = 6.8–13.5; blue–green	4–5 to 5	4–5 to 5	Color-focused	No repeated wash cycles	Strong fastness; limited functional data	80
Alizarin anthraquinone	PA66	Supercritical CO_2_ dyeing	Σ*K*/*S* = 52.9–102.3	Shade changed after washing	1–2 after 40 h	Deep shade	Severe light fading	High depth did not equal durability	68
*Bambusa vulgaris* flavonoids	Nylon 6	Cu/Sn/Fe mordants	*K*/*S* = 4.2 directly; 8.6 Fe	Improved	6–7 or 7	Coloration	No repeated washing	Excellent photostability requires metal salts	133
*Scrophularia* + AgNPs	PA6 knit	Extract with Ag ions/NPs	*K*/*S* = 4.1–5.6	Good	8	100% *E. coli*/*S. aureus* reductions	After 10 washes, 79–88% retained	Durable antimicrobial but Ag release unknown	173
Turmeric/madder/catechu/indigo	PA56	Natural dyes; aloe gel post-mordant	*K*/*S* = 14.51–19.76 selected	Good	3–5 after aloe	Color-focused PA56 comparison	No long-term function	PA56 dyes well; light remains dye-dependent	70
*Prangos* + natural mordants	100% nylon	*Carthamus*, *Terminalia*, pomegranate	*K*/*S* = 9.40 *Carthamus*; 9.02 *Terminalia*	5	7–8 or 8	Color and light stabilization	Single-cycle fastness	Natural mordants can outperform metals; hue shifts	131
Kapok leaf extract	Nylon 6	Al/Cu/Fe pre-mordanting	*K*/*S* = 14.690 with Fe	Good	5 with Cu/Fe	UPF = 82.98; 92.63%/83.54% antibacterial; 94.42% antioxidant	No functional durability	Strong initial multifunctionality; metal/durability gap	135
Industrial plant dyes	PA interlock knit	Jet dyeing with catechu, madder, pomegranate, and mulberry	Commercial brown/red/pink/green shades	Good	1/2–3/4	Excellent UV protection	Repeated wash did not change L* significantly	Industrial relevance but weak light for some dyes	76
Curcumin smart dressing	PA6 fraction	1.0–1.5 g L^−1^ curcumin	Visible pH response; 1.0 g L^−1^ selected	4 at 1.0 g L^−1^	Not central	pH sensing; HaCaT compatible; hemolysis <2%	Washing/perspiration grade 4	Higher depth reduced fastness	106
Baicalin flavone	Nylon 6	Mordant-free exhaust	*K*/*S* = 7.8 at 8% owf; 4.1 at 3% owf	3–4 to 4–5	Not main limitation	UPF = 40+ before washing	UPF = 30 + after 5–30 washes; 26.12 after 50	Best UV durability; *K*/*S* decreased	47
*Aspergillus turcosus* pigment	PA6	Direct or alum; pH 4	*K*/*S* = 2.5; RUI 0.04	Alteration = 4; staining = 4–5	3–4	Antibacterial, antioxidant 45%, UPF = 15, pH response	After 20 washes, zones retained with ∼32% loss	Broad function but modest UPF/light fastness	44
*Beta vulgaris*-derived azo dye	Plain PA	Mordant-free derivatives	*K*/*S* = 18.85 and 13.07 at 1%	3–4 to 4	6	82.59–91.84% microbial reduction; UPF = 25.175–27.0225	No functional wash durability	High depth/function; wash/wet rubbing moderate	177

To complement the system-level comparison in Table 4, the highest reported quantitative performance values identified for the major natural-colorant classes are consolidated in Table 5. These values are presented as reported maxima rather than normalized rankings because nylon grade and fabric construction, processing conditions, *K*/*S* calculation methods, mordanting or auxiliary systems, and functional test protocols differed among studies.

**Table 5:** Highest reported quantitative performance metrics identified for major natural-colorant classes on nylon/polyamide textiles^a^

Natural-colorant class	Highest reported *K*/*S*	Best reported wash fastness	Highest light fastness	Highest reported UPF	Highest reported antibacterial activity	Ref.
Tannins/polyphenols and tannin-rich extracts	21.94, *T. catappa*	5 in selected phenolic/tannin-rich systems	5, *T. catappa*	2000 in industrial tannin-rich systems; *T. catappa* reached 1995 from an undyed value of 26.39	>99.9% *S. aureus* reduction in spent-coffee-ground dyed nylon	92, 96, 132 and 140
Flavonoids and flavonoid-rich extracts	17.999, sea-buckthorn extract	4–5 color change and 5 staining, hawthorn	7, hawthorn	764.1, sea buckthorn; purified luteolin reached 205	99.8–99.9%, sea-buckthorn dyed nylon/PU	72, 97 and 122
Anthraquinones	18.77, rhubarb anthraquinones with walnut-husk biomordant; Σ*K*/*S* = 102.3 for scCO_2_ alizarin^b^	5	8	1818, industrial madder system^c^	NR in a directly comparable percentage format	68, 92 and 120
Curcuminoids	∼18.1, electron-beam-assisted curcumin	5	3–4	105.6 ± 7.7, poloxamer-stabilized curcumin nanoparticles	>99.9% *S. aureus* reduction	39, 42, 105 and 172
Indigoids	13.5, bio-indigo/indigoid system	Up to 5	Up to 5	NR	NR	79
Microbial pigments^d^	8.43, red *Monascus* pigment	4 for color change; staining up to 5	3–4, *Aspergillus* pigment	15.1, prodigiosin system	100% against *S. aureus* and *P. aeruginosa* in a prodigiosin-DES system	36, 44, 158 and 163

a Values represent the highest values identified within the reviewed literature and may originate from different studies within the same colorant class; therefore, the metrics within a single row should not be interpreted as performance achieved simultaneously by a single dyeing system.

b Σ*K*/*S* reported for supercritical-CO_2_ alizarin is a summed spectral value and is not directly comparable with conventional single-wavelength *K*/*S*.

c The industrial fabric showing UPF values of 1818–2000 had an unusually high undyed UPF of 1324.51; therefore, these values are strongly influenced by fabric construction. Antibacterial percentages are reported separately from inhibition-zone measurements because the two methods are not directly equivalent.

d Microbial pigments in this row exclude microbial indigoids and fungal anthraquinones, which are represented under their respective chemical classes. NR = not reported in a directly comparable quantitative form.

## 8 Cleaner-production performance and industrial readiness

### 8.1 Natural origin *versus* verified cleaner production

Natural origin is only the first condition for cleaner nylon coloration; it is not proof of cleaner production. A comprehensive assessment must include biomass production or recovery, collection, drying, grinding, extraction, concentration, dyeing, fixation, washing, effluent treatment, product lifetime, and end-of-life behavior. In the reviewed nylon/polyamide studies, most cleaner claims were based on replacing synthetic dyes with plant, microbial, animal, or waste-derived colorants, while fewer papers quantified wastewater, energy, toxicity, or life-cycle outcomes.

Therefore, the use of the terms ‘greener’ and ‘cleaner’ in this review is relative and conditional and does not represent overall environmental excellence. Reported advantages, such as the use of renewable feedstocks, metal avoidance, shorter processing and reduced water and energy consumption, were considered in conjunction with disadvantages, such as residual metal content, COD/BOD and organic burden, solvent recovery, oxidant/reductant residues, and product durability. In the absence of quantification, the environmental advantages were regarded as provisional instead of proven.

Enzyme-mediated catechol/hydroquinone and catechol/gallic-acid systems avoided metallic mordants and operated at 55–60 °C, but they still used HRP, H_2_O_2_, and aqueous buffers, and residual phenolics or oxidant residues were not measured.^32,33^ Rice-straw coloration valorized agricultural waste and avoided metallic mordants, yet its extraction route used alkali, acidification, n-butanol, and methanol; therefore, the cleaner claim depends on solvent recovery and effluent control rather than feedstock origin alone.^20^ Similarly, indigoid bio-*in situ* dyeing reduced dependence on sodium dithionite and separate dyeing baths, but characterization of the textile wastewater composition and evidence of fastness were still incomplete.^34^ Therefore, cleaner-production performance should be judged as a balance between renewable input, auxiliary burden, dye utilization, washing durability, effluent quality, and useful product lifetime. A natural dye that fades rapidly requires repeated dyeing, or relies on high metal salt doses may be less sustainable than a more durable low-auxiliary route.

### 8.2 Feedstock sustainability and circularity

The selection of the feedstock determines the potential for circular coloration using natural colorants, but the use of natural sources may consume more resources. Cultivated dye plants, including madder, weld, turmeric, saffron, safflower and plants containing baicalin, provide relatively well-defined shade families, yet may be limited in supply due to land use, seasonality, crop quality, transport, and competing food, medicinal and heritage uses.^13,35,39,47^ Conservation-sensitive Brazilwood can be used in reference colorimetry, but its general use in industrial sourcing is not suitable.^98^ Waste biomass and by-products are better candidates as they utilize existing streams, such as rice straw,^20^ spent coffee/roasted peanut skin 37, pomegranate peel,^45^ teak leaves,^139^ tomato residues,^146^ eucalyptus residue,^153^ almond black liquor,^143^ olive vegetable water,^43^ and timber bark.^151,154,156^ These sources minimize the pressure on crops, but changes in pigment/tannin ratios may occur with various plant parts, different maturity, storage conditions, extraction pH, solid-to-liquid ratio, and regional availability. Liquid industrial by-products, such as eucalyptus residue and olive vegetable water, are attractive due to reduction in extraction steps, but they are localized streams with a high organic load.^43,153^ Microbial pigments overcome seasonality and land dependency, and fermentation can be controlled by strain, medium, pH, temperature, and time. Additionally, microbial production can offer a more continuous and geographically independent pigment supply compared to cultivated plant sources, and waste-derived fermentation substrates can further enhance the circularity of the process. Indigoidine production illustrates this potential because soybean okara hydrolysate increased the 5 L bioreactor titer from 1.81 to 5.25 g L^−1^ after 78 h.^157^ However, fermentation transfers part of the resource burden to culture media, sterilization, aeration, agitation, and downstream recovery. Production time can also be substantial; for example, the *Streptomyces* route required six days of fermentation followed by acidified ethyl acetate extraction and methanol/water redissolution.^160^ Bio-indigo additionally required purification of bacterial residues and indirubin, whereas crude-gel prodigiosin demonstrated that direct or minimally purified pigment application can reduce downstream processing.^79,158^ Thus, microbial pigments can reduce land and seasonal constraints but are not inherently lower-impact unless medium composition, fermentation productivity, purification, and solvent requirements are considered.

Scale-up remains to be accomplished, as culture stability, pigment production, downstream recovery, and regulatory acceptance of these materials remain to be addressed.^44,157–159^ Recycled nylon 6,6 and bio-based PA56 can also promote the circularity of the substrate, but the dyeing phase should also be independently verified for environmental performance.^35,70,97^ The indigoidine example remains both promising and challenging because, despite the improved fermentation productivity obtained with SOH, crude pigment purity was only about 66.3%, and nylon dyeing still used DMSO-assisted solubilization or sodium hydrosulfite reduction; therefore, feedstock circularity has not yet been translated into a fully circular, dyehouse-ready process.^157^

### 8.3 Chemical safety and auxiliary burden

Mordanting is the primary chemical-safety consideration. Mordant-free systems reduce the risk of metal-containing effluent and have yielded useful nylon coloration using phenolic, tannin-rich, curcuminoid, indigoid, microbial, and anthraquinone dyes.^39,47,68,80,89,140,153,176^ However, their performance is not always adequate, particularly in the case of light fastness. Metal salts can be used to enhance shade depth, light fastness, and washing durability, but their cleaner value is related to the associated hazard and the control of metal residues. Chromium(vi), copper sulfate, stannous chloride, zinc salts, and zinc chloride are of high concern because of their carcinogenic, toxic, cytotoxic, aquatoxic, corrosive, or marine-pollution risks.^37^ Therefore, potassium dichromate is not suitable, even with good performance. Chromium, in a *Prangos* dyeing system, provided a *K*/*S* of 8.46 and light a fastness of 7, but the authors preferred natural mordants due to the environmental risk.^131^ Some systems were improved with copper or tin, but the amount of copper or tin present as spent-bath Cu(ii) or Sn(ii) was not often quantified.^151^ Alum and iron are lower-concern options, but must still undergo residual metal analysis. Biomordants, including chitosan, soy milk, tannic acid, aloe vera, pomegranate, myrobalan, rhubarb extract and lemon juice, would help reduce dependency on heavy metals, although they are not impact-free due to their organic load, pH demand, viscosity, odor and microbial-stability concerns and batch variability.^19,42,57,76,83^ There is also an important role played by auxiliary chemicals. Acids and alkalis are regularly employed in nylon protonation and extraction, sodium hydrosulfite is a reductant burden in some indigoid routes, H_2_O_2_ is employed in enzymatic oxidation, and organic solvents, such as ethanol, methanol, n-butanol, acetone, DMSO, hexane, and dichloromethane, are used in several extraction or pigment-recovery routes.^20,114,115,157,166^ Nanoparticle-assisted routes may increase fastness and antibacterial activity; however, their release during laundering should be carefully measured prior to accepting a cleaner claim.^35,87,173^ Deep eutectic solvents are promising as water-free media, but there are challenges with their acidity, recyclability, toxicity, and end-of-life recovery that can only be demonstrated directly.^158^

### 8.4 Water, energy, and process efficiency

Most nylon natural-dyeing systems still use conventional aqueous exhaustion. The common practical conditions are around 80–100 °C, 30–90 min, and liquor ratios of around 1/20–1/50, although lower-liquor industrial or sportswear-oriented studies used 1/10, while some laboratory affinity studies used unrealistic liquor ratios, *i.e.*, 1/100, 1/500, *etc.*^62,73,76,100,109,112^ This is particularly important because a high-liquid-volume study may have proved affinity but not industrial water efficiency. Process intensification has yielded measurable improvements to date, but with some mixed evidence. Microwave-assisted systems shortened extraction or dyeing times to 1–10 min in some cases, improved *K*/*S*, and in *Aegle marmelos* dyeing reduced the optimized Nylon 6 dyeing time from 120 to 40 min while increasing exhaustion to 93%.^13,67,83,92,127^ Mass transfer through cavitation was enhanced by ultrasound. One study reported an input of150 W for 1 h compared with 3000 W for water-bath dyeing, representing a 95% reduction in power input, but the shade performance of *Thespesia populnea* was lower under ultrasound than under conventional heating; thus, energy saving alone is not enough when the shade performance decreases.^37,123^ Plasma and laser are dry pretreatments, which can also improve dyeability without wet chemical scouring, but they have narrow processing windows. A 12 W laser improved pomegranate-peel *K*/*S* by 55.7%, while 16–20 W caused melting and fiber adhesion.^45^ Plasma improved indigo dyeing kinetics by 2.8-fold, and dry plasma treatment improved berberine uptake with less than 1% breaking-load loss.^41,65^ Low-water routes remain less mature. scCO_2_ dyeing of PA66 with alizarin eliminated the aqueous dyebath at 120 °C and 34 MPa, but fastness limitations and energy/pressure costs remain unresolved.^68^ Choline chloride/lactic acid DES improved the *K*/*S* of prodigiosin from 2.30 to 3.33 and equalization from Δ*E* 0.05 to 0.01, but DES recovery and toxicity were not addressed.^158^ Cold pad-batch minimized steam consumption but required 24 h batching. Pad-dry-steam used 8 kg steam per kg fabric, while the open bath used 1.5 kg kg^−1^, and HTHP used 5 kg kg^−1^.^64^ Bio-based nanoparticle routes show a similar trade-off because most sugarcane-bagasse lignin nanoparticle dispersions required only 0.5–1.0 kWh kg during refining, whereas the highest-polyamide-color-strength LNP-185-5C condition required 12 kWh kg, so the best *K*/*S* response was also the most energy-intensive option.^149^

### 8.5 Effluent quality, toxicity, and environmental evidence

The weakest part of the current literature is effluent evidence. Most articles report *K*/*S*, CIELAB, fastness, and functionality, but omit COD, BOD, residual color, dissolved solids, residual phenolics, salts, pH, metal content, toxicity, biodegradability, and dyebath reuse quality. Where effluent data exist, they challenge the assumption that natural dyeing is automatically cleaner. Studies on spent coffee and roasted peanut skin dyeing included reused effluents with high COD values of 11 000 and 4875 mg L^−1^ and BOD values of 3068 and 1614 mg L^−1^, respectively.^37^*Terminalia arjuna* and *Thespesia populnea* dye-bath reuse also produced high organic loads, with COD/BOD values of 3290/1268 mg L^−1^ and 14 937/4954 mg L^−1^, respectively.^123^ These values suggest that plant-derived phenolics and melanoidins may be biodegradable but still impose substantial oxygen demand, so wastewater treatment remains necessary.

Solvent recovery, post-extraction residues and dyebath reuse are also important parameters to consider in the context of effluent quality for credible circularity assessment. Routes that use solvents should report on the efficiency of solvent recovery, the possibility of reusing the solvent, the energy recovered during the extraction, and the residues to be managed or valorised, while pathways based on plant feedstocks should include the amount of biomass residues to be managed or valorised, not just because they are waste but also because they are a valuable resource. Dyebath reuse can help to reduce the amount of fresh water used and the amount of colorant required, but multiple reuses can change the colour shade and bath composition; for instance, almond liquor was reused for 5 dyeings of polyamide, with the *K*/*S* values decreasing from 15.82 in the first reuse to 10.81 in the fifth.^143^ Therefore, future studies should report the number of reuse cycles, shade/*K*/*S* retention, chemical replenishment, and final bath quality.

Olive vegetable water is a special case because the dye liquor itself was an agro-industrial wastewater; ultrasonic dyeing reduced total polyphenols from 10.43 to 9.58 g L^−1^ and COD from 70.02 to 61.04 g L^−1^, showing partial pollutant removal during coloration rather than merely producing a new effluent.^43^ Where toxicity evidence exists, it is useful but rare. Curcumin nanoparticle dyeing reported nonphytotoxic, noncytotoxic, and nongenotoxic wastewater, with germination generally above 90% and *Allium cepa* mitotic-index values above 90% for selected wastewater systems.^172^ In contrast, many metal-mordanted, solvent-extracted, nanoparticle, DES, and reductive indigoid systems did not quantify residual metals, residual solvents, nanoparticle shedding, hydrosulfite by-products, or aquatic toxicity. Therefore, future cleaner claims should require at least COD, BOD, residual color, pH, conductivity/TDS, total phenolics, residual metal or nanoparticle content, acute toxicity, and biodegradability, together with solvent recovery, post-extraction residue management, and dyebath-reuse performance where applicable, before industrial recommendation.

### 8.6 Available life-cycle and techno-economic evidence and the prevailing data deficit

Life-cycle and techno-economic evidence is scarce. Most studies infer sustainability from natural origin, waste valorization, or shorter processing times rather than calculating impacts per kilogram of dyed nylon. The strongest evidence was reported for microbial indigoidine production, where SuperPro Designer and SimaPro/Ecoinvent modeling compared four production scenarios. The best scenario, fermentation with SOH plus sodium hydrosulfite extraction, reduced global warming potential from 9 883 760.5 to 1 516 445.4 kg CO_2_ eq and water consumption from 72 975.37 to 20 672.92 m^3^ compared with the least favorable scenario, although terrestrial acidification was higher than that of one alternative.^157^ This microbial example also illustrates that production economics depend on fermentation productivity, medium selection, and downstream recovery rather than season-independent production alone. The SOH-assisted indigoidine process reached 5.25 g L^−1^ after 78 h, and the most favorable modeled scenario reported a unit production cost of 52.50 US$ per kg of pigment.^157^ For nano-prodigiosin, laboratory chemical and biological reagent costs were approximately 0.135 yuan per gram of silk/polyamide fabric compared with approximately 0.091 yuan for conventional weak-acid dyeing, although the microbial route shortened the dyeing time and additionally provided antibacterial and UV-protective functions.^36^ Downstream processing is also an important factor in cost; bacterial residues had to be removed from bio-indigo,^80^ while the crude-gel prodigiosin did not have to undergo extensive purification before use as a pigment.^158^ The bath-reuse potential of the *Monascus* nano-suspension dyeing was also shown, but details of the production cost, energy balance, and life-cycle data were not reported.^163^ These examples illustrate that microbial pigments offer promising scalability and supply benefits, but are economically competitive if the media are inexpensive, the pigment concentration is high, the fermentation time is short, the pigment recovery is simple, and the pigment is directly compatible with textile dyeing.

Cost evidence is similarly limited. One nylon 6 study gave direct dyeing-cost comparisons. Unmordanted henna cost 0.42–0.44 US$ per kg nylon compared with 0.95–0.97 US$ per kg for shade-matched acid yellow dye and 0.81–0.83 US$ per kg for disperse yellow dye; however, adding 20% tannic acid increased henna dying cost to 1.08–1.14 US$ per kg, showing that biomordant dosage can erase the cost advantage.^22^ Some waste streams were described as free or low-cost, such as olive vegetable water and eucalyptus residue, but practical costs still include collection, filtration, storage, extraction/concentration, heating, equipment, effluent treatment, quality control, and transport.^43,153^ Pigment yield and downstream processing remain major gaps because low plant pigment concentration, solvent recovery, membrane fouling, fermentation time, lyophilization, column purification, and high-pressure scCO_2_ equipment can increase cost and energy requirements.^71,119,154,157^ Direct benchmarking against commercial dyes is also rare. In the fungal anthraquinone comparison with CI Disperse Red 60, emodin and dermocybin showed high polyamide uptake and good rubbing fastness, but their light fastness on polyamide was only 3 and 1 *versus* 5 for the commercial reference, and emodin had poor washing color change (Δ*E* of 9.80; grade 1–2), indicating that laboratory natural-dye affinity is not enough for commercial substitution.^167^

### 8.7 Standardization and quality control

Standardization is a prerequisite for industrial readiness, in addition to achieving laboratory-scale color strength. Botanical or microbial identity, plant part, harvest season, moisture content, particle size, storage conditions, extraction pH, solid-to-liquid ratio, marker-compound concentration, pigment purity, microbial contamination limit, and extract stability are some of the required specifications. Several studies show why this is necessary. Teak-leaf extraction gave the best gravimetric dye yield at pH 9, but the best unmordanted nylon *K*/*S* was obtained at pH 7, suggesting that yield alone does not ensure dyeing performance.^139^*Terminalia arjuna* and *Thespesia populnea* extracts varied significantly in total phenolic content, with 62.19 ± 0.03 *versus* 10.08 ± 0.04 mg/100 g under water-bath extraction, which explains why nominally similar plant dyes cannot be replaced without profiling.^123^ Sesame extract was found to be useful in absorbance standardization *via* Beer–Lambert behavior (*R*^2^ = 0.9979), and the extraction of carotenoid compounds from tomato residue was able to yield close predicted and experimental yields of 80.7 µg g^−1^ and 79.8 µg g^−1^, respectively, but there is still a need for compound-level fingerprinting and shade validation.^77,146^ Commercial dyehouses also need shade libraries, Δ*E* tolerances, storage-stability windows, filtration specifications, reproducible auxiliaries, and clear pass/fail criteria for washing, rubbing, light, perspiration, and functional durability.

### 8.8 Industrial-readiness classification

The reviewed routes can be grouped into five readiness levels. Level 1, proof of concept, covers visible nylon coloration without standard fastness, effluent, reproducibility, or scale-up data, including early bio-*in situ* indigoid embroidery, oil-carried fungal pigments, and solvent-extracted plant systems.^34,166^ Level 2, laboratory validation, includes *K*/*S*, CIELAB, fastness, and functional data under beaker or IR-dyeing conditions; many mordant-free plant, microbial, baicalin, curcumin, tannin, and enzymatic systems remain here.^32,33,39,44,47,97^ Level 3 covers statistically optimized RSM/CCD, ANN-GA, factorial, kinetic, or adsorption-modelled studies such as microwave annatto, *Aegle marmelos*, coffee pomace, and lac systems.^60,62,92,127,170^ Level 4 includes pilot-relevant machinery, low liquor ratios, semi-industrial plasma, pad-batch, scCO_2_, melt-spinning, fermentation scale-up, or repeated-dyeing trials without full commercial validation.^64,68,71,73,93,157^ Level 5, industrial demonstration, is rare. The best example is the use of catechu, madder, pomegranate peel and mulberry leaf dyes in a Thies jet machine at 3% owf, 98 °C, 45 min and 1/10 liquor ratio, in which there is no significant washing-cycle effect on the L* value, as determined through repeated washing and ANOVA.^76^ Product-wise, brown and yellow–brown systems with tannin/polyphenol pigments are most realistic, as they tend to be produced using waste feedstocks, maintain wet/perspiration fastness and provide good UV protection. Red/pink anthraquinones, which are more likely to be pigmented with mordants and can have a stronger shade identity than flavonoid or carotenoid dyes, are often more photostable. Green shades remain less reliable because chlorophyll sources are unstable; two-step indigo/turmeric or selected mulberry-leaf routes are more plausible.^70,76^ The most advanced added functionality is UV protection, while the antibacterial benefits still require demonstration in terms of washing durability and nanoparticle/metal release.^20,47,72,96,173^ Rice straw gives the strongest waste-derived functional-durability evidence, with plasma-treated nylon retaining UPF from 79.9 to 69.0 and antioxidant activity from 39.6% to 34.9% after 20 washes; this level of value retention is more industrially meaningful than single-wash or unwashed functionality.^20^ A comparative cleaner-production and industrial-readiness assessment of the major natural-dyeing route families is presented in Table 6.

**Table 6:** Cleaner-production and industrial-readiness assessment of major natural-dyeing routes

Process family	Renewable/waste feedstock	Hazardous inputs	Water demand	Energy demand	Durability	Effluent evidence	Cost and scalability challenges	Scale	Readiness level	Ref.
Mordant-free aqueous plant dyeing	Cultivated or collected plant colorants; strongest when abundant or waste-derived	Usually low; acids/alkalis for nylon pH control may remain	Typically 1/20–1/50; some 1/10 industrial and many 1/100 laboratory studies	Mostly 80–100 °C for 30–90 min	Wash/rub fastness is often 3–5; light fastness is commonly weak at 1–4 unless dye is indigoid, anthraquinone, or stabilized	Mostly missing COD/BOD/toxicity; high exhaustion sometimes used as indirect evidence	Direct cost data are scarce; extraction, high liquor ratios, heating, extract standardization, and quality control can affect commercial economics	Lab to industrial for selected dyes	2–5	39, 47, 76, 89 and 153
Waste-biomass and by-product dyeing	Rice straw, coffee/peanut waste, pomegranate peel, teak leaves, tomato residue, eucalyptus residue, almond black liquor, olive wastewater	Usually low to moderate; solvents or metals appear in some routes	Aqueous extraction/dyeing common; liquid residues can reduce extraction water	Boiling/exhaust dyeing common; ultrasound/plasma may reduce selected steps	Good wet fastness for tannin-rich sources; light often source-dependent	Best evidence shows COD/BOD can remain high; olive wastewater shows partial COD/polyphenol reduction	Low-cost or waste feedstocks may still require collection, storage, transport, filtration, extraction, standardization, and effluent treatment, limiting scale-up when supply is local or variable	Lab to pilot-relevant; local supply chains	2–4	20, 37, 43, 123, 139, 143, 146 and 153
Metallic mordant-assisted dyeing	Natural dyes with alum, Fe, Cu, Sn, Zn, or Cr salts	Moderate to high; Cr, Cu, Sn, and Zn raise toxicity and metal-residue concerns	Often conventional aqueous multi-step or meta-mordanting	Extra mordanting adds time/energy; some 1/10 meta-mordant routes reported	Improves *K*/*S* and light fastness; some Cr/natural mordants reach light fastness of 7–8	Residual metal data often absent; Cu/Sn spent bath not quantified in key studies	Additional mordanting stages, chemical handling, residual-metal monitoring, wastewater treatment, and regulatory requirements can increase operational complexity; comparative cost data remain limited	Lab; selected industrial and sportswear-relevant trials	2–4, but cleaner score depends on metal choice	37, 73, 76, 78, 131 and 151
Biomordant and bio-based fixation	Chitosan, soy milk, tannic acid, aloe vera, myrobalan, pomegranate, rhubarb, lemon juice	Lower metal hazard but adds organic load, pH demand, variability, and possible microbial stability issues	Mostly aqueous; pretreatment may add baths or long contact times	May require extra pretreatment/drying; some one-bath options reduce steps	Can improve wash/light/functional durability; effect is dye-specific	Organic COD and biodegradability rarely measured	High dosage, extract preparation, batch variability, and additional impregnation/drying/curing can increase cost and complicate scale-up. At 20% tannic acid, henna dyeing cost increased to 1.08–1.14 US$ per kg nylon.^22^ Film-forming biomordants also require evaluation of fabric handle and coating durability	Mostly laboratory; some industrial-scale auxiliary use	2–5	19, 22, 42, 57, 76 and 83
Microwave/ultrasound intensified dyeing	Compatible with plant/waste dyes and some biomordants	Depends on extraction medium; acidified methanol, acetone, or salts may remain	Usually aqueous; reduced time more proven than reduced water	Microwave for 1–10 min in some cases; ultrasound at 150 W *vs.* 3000 W in one comparison; not always higher *K*/*S*	Can improve exhaustion, *K*/*S*, and fastness; over-treatment may reduce performance	Energy and effluent data rarely paired with fabric results	Equipment cost, treatment uniformity, bath geometry, and poorly standardized energy reporting limit reliable scale-up and economic comparison	Laboratory to statistically optimized	2–3	13, 37, 82, 83, 92, 123, 127 and 170
Dry surface activation and physical pretreatments	Used with natural plant dyes; no dye feedstock change	Low wet-chemical burden, but electricity/equipment required	Pretreatment is dry; dyeing often still 1/40–1/100 aqueous	Plasma/laser/ozone/EB can shorten or intensify dye uptake; high dose can damage fibers	Improves *K*/*S*/light/UPF in selected cases; dose control critical	No full wastewater or energy balance in most cases	Specialized equipment, dose uniformity, treatment ageing, fiber-damage control, and continuous-line integration remain practical scale-up constraints; economic evidence is limited	Lab to semi-industrial plasma	2–4	20, 41, 45, 58, 65, 93 and 105
Waterless/low-water media and solid coloration	scCO_2_ alizarin, ChCl/LA DES prodigiosin, D5 *Monascus*, melt-spun DNP polyamide; oil-carried fungal pigments	Avoids aqueous bath, but pressure media, solvent/DES recovery, or wet pigment synthesis remains; DCM/oil-carrier recovery may remain	Very low dye-bath water; not always zero water across full route	scCO_2_ uses 120 °C and 34 MPa; DES uses 80 °C; melt compounding uses 260 °C; LNP refining uses 0.5–12 kWh kg^−1^	High shade possible but fastness or processability may limit translation; slow drying/fading can limit coatings	Effluent low at dyeing step, but solvent/DES/CO_2_ recovery and LCA often missing; DCM and carrier stability also need evidence	High-pressure or specialized equipment, media recovery, energy demand, and carrier/solvent management can dominate capital and operating requirements	Proof to pilot-relevant	1–4	68, 71, 158, 164 and 66
Microbial and bio-*in situ* pigments	Fermentation-derived prodigiosin, indigoidine, *Monascus*, *Aspergillus* pigment; SOH-supported indigoidine reached 5.25 g L^−1^	Potentially avoids cultivated land; extraction may use methanol, DMSO, hydrosulfite, or DES; long fermentation may remain	Bio-*in situ* reduces separated baths; many routes remain aqueous	Fermentation time can be 5–11 days; downstream recovery can dominate energy/cost	Good antimicrobial/UV potential; light fastness often limiting for prodigiosin/violacein	LCA/TEA strong in one indigoidine production study; dyehouse effluent usually absent; crude purity and hydrosulfite/DMSO residues remain gaps	Fermentation time, culture consistency, sterility, pigment productivity, and downstream separation/purification can dominate production cost and hinder reproducible scale-up	Lab to production-simulated fermentation	1–4	34, 44, 157–159 and 161
Circular substrate systems	Recycled nylon 6,6 or bio-based PA56 combined with natural dyes	Depends on dye/fixation route; can still require alum, metal ions, or post-finishes	Usually conventional aqueous dyeing	Often 80–90 °C, 60 min; finishing may add stages	Good wet fastness possible; light fastness remains a recurring limitation	Substrate circularity reported more often than dyehouse effluent	Economic and scale-up performance depends on consistent recycled/bio-based substrate supply and on whether the selected coloration route adds costly mordanting, finishing, or quality-control stages	Lab to industrial for recycled PA66 route	2–5	35, 70, 76 and 97

The most defensible cleaner-production routes for nylon are not the most visually novel, but those combining waste or abundant feedstocks, low-hazard fixation, measurable durability, and compatibility with existing equipment. Tannin- and polyphenol-rich brown/yellow–brown systems from pomegranate peel, catechu, eucalyptus residue, almond black liquor, spent coffee, and *Terminalia*-type leaves are more industrially plausible than solvent-dependent or rare-source dyes, especially for innerwear, sportswear, low-light apparel, and UV-protective products. Red/pink anthraquinones such as madder and cochineal have greater shade identity and can sometimes be more photostable, but there are still many methods that require alum or other mordants. Yellow curcumin, weld, luteolin and baicalin systems are suitable for multifunctional UV, antioxidant, antimicrobial or pH-responsive fabrics, with the exception of light fastness and washing durability, which depend on the product. Blue/indigoid routes are promising with regard to color identity and photostability; however, cleaner production would involve hydrosulfite/alkali replacement and validation of bio-based scale-up. The field has moved beyond proving that nylon can be naturally dyed; the task is proving cleaner performance per kilogram of durable dyed fabric. Therefore, the most urgent evidence gaps are paired LCA-effluent studies, commercial-dye benchmarking, release testing for nanoparticles or metals, and durability data after repeated washing, abrasion, perspiration, and light exposure.

## 9 Research gaps and future roadmap

The reviewed literature confirms that nylon can be colored with plant, animal, microbial, waste-derived, enzymatically generated, and nano-formulated natural colorants. However, the field is still dominated by lab demonstrations where the shade depth, fastness, utility, and environmental benefit are not assessed equally. Many papers report promising *K*/*S*, UV protection, antibacterial activity, or waste valorization, but few integrate chemical standardization, nylon-specific mechanism validation, repeated-use durability, wastewater data, and process economics. Thus, future research should focus on verified cleaner coloration rather than proving color only, with a focus on both performance and environmental claims.

### 9.1 Minimum reporting requirements

A mandatory reporting framework is required because there are variations in studies according to nylon grade, fabric structure, dye strength, mordanting route, and test method. The authors are expected to report the grade of nylon used, construction, areal density/yarn count, pretreatment, extract yield, active colorant, dye concentration, pH, temperature, time, liquor ratio, mordant type and dosage, replication, statistics, and fastness, UPF, antibacterial, antioxidant, and cytotoxicity assay standards. These data are required to make reliable comparisons of *K*/*S* and fastness differences.^20,33,35,57,76,158^ In addition to reporting the test method, future studies should preferentially adopt a common reference standard within each performance category so that differences among studies are less influenced by the testing procedure itself. The framework consolidates the recurring reporting gaps identified throughout this review into six domains: substrate identification, colorant characterization, processing conditions, performance testing, experimental reliability, and environmental assessment. The minimum information recommended for each domain is summarized in Table 7.

**Table 7:** Proposed minimum-reporting framework for the natural coloration of nylon/polyamide textiles

SI	Reporting domain	Minimum information to be reported
1	Nylon/polyamide substrate	Nylon grade (*e.g.*, PA6, PA66, and PA56), pure or blended composition, fabric/yarn form, construction, areal density or yarn specification, and pretreatment
2	Natural colorant/extract	Biological source and source part, extraction method and conditions, extract yield, dosage basis, and, where feasible, marker-compound concentration, pigment purity, or UV-vis/HPLC/LC-MS fingerprint
3	Dyeing and fixation process	Dye concentration, pH, temperature, time, liquor ratio, mordant/biomordant type and dosage, auxiliary chemicals, and relevant pretreatment or process-intensification conditions
4	Coloration, fastness, and functionality	*K*/*S* measurement conditions and CIELAB data should be reported together with the exact test standard and its edition/year. For greater harmonization, ISO 105-C06 is recommended as the reference method for washing fastness, ISO 105-X12 for rubbing fastness, ISO 105-B02 for light fastness, and ISO 105-E04 for perspiration fastness. AATCC TM100 is recommended for quantitative antibacterial assessment and AATCC TM183 for UPF measurement. Antioxidant and other functional assays for which a single textile-specific reference method is not consistently established should include the complete assay procedure, endpoint, and calculation method
5	Experimental reliability	Number of replicates, variability or error measures, statistical analysis where appropriate, controls/comparators, and sufficient raw process conditions to permit reproducibility
6	Environmental performance	For studies making cleaner or sustainability claims, relevant water and energy information, effluent pH/color, COD/BOD, residual metals, solvents, oxidants/reductants or other process-specific residues, toxicity, and dyebath reuse or recovery information should be reported where applicable

Where the recommended reference method cannot be used, the alternative recognized standard should be identified by its complete designation and year, together with the specimen preparation, test conditions, endpoint, and calculation procedure. Results obtained using substantially different test principles should not be considered directly interchangeable. For example, antibacterial inhibition-zone measurements should be distinguished from quantitative percentage reduction or log-reduction methods. Consistent adoption of reference methods would substantially improve inter-study comparison without requiring previously published results obtained by different standards to be retrospectively converted or normalized.

### 9.2 Molecular characterization and mechanism validation

Most of the mechanisms are still hypothesized from the pH-dependent *K*/*S*, FTIR shifts, SEM morphology and fastness data, which support plausible interactions between protonated amino end groups, amide carbonyls, hydrogen bonding, ionic attraction, hydrophobic association, metal bridging and π-related interactions.^20,35,36,40,44,57,79^ The abnormal cases need to be further validated, since chromium or iron mordants can have a positive effect on *K*/*S* and light fastness, but increase environmental concerns,^131^ as evidenced by the introduction of carcinogenicity, cytotoxicity/genotoxicity, aquatoxicity, corrosiveness, and marine-pollution concerns with the use of chromium(vi), copper sulfate, stannous chloride, and zinc salts.^37^ Some near-neutral systems additionally suggest that hydrophobic partitioning, not ionic bonding, is the mechanism involved.^61^ Further research is recommended to be done in combination with HPLC/LC-MS/NMR profiling, XPS, Raman/ATR-FTIR depth analysis, zeta potential, adsorption/desorption tests, exhaustion/fixation mass balance, molecular modeling and purified-*versus*-crude comparisons.

### 9.3 Multi-objective process optimization

The optimization goals should not be limited to *K*/*S* anymore. Most reported processing windows for nylon natural dyeing are in the range of acidic pH 2–5, 60–100 °C, 45–90 min, and liquor ratios of 1 : 20–1 : 100, with the exception of mild enzymatic coloration at 55–60 °C,^33^ water-free DES dyeing,^158^ supercritical CO_2_ dyeing,^68^ and microwave-/ultrasound-assisted route.^92,127^ These ranges are indicative of operating tendencies rather than absolute conditions, as acidic baths may lead to increased exhaustion due to protonated amino end groups, but may also increase the effluent acidity, cause shade shifts or increase the fiber damage risk, while near-neutral, solvent-assisted or surface-deposition systems may use hydrophobic partitioning, pigment aggregation or coordination.^40,61,158^ The important thing is that the deeper the color, the higher the energy consumption, liquor ratio, solvent usage, and metal residue, or lower levelness. Future RSM, desirability and AI-assisted designs should work together to optimize digital shade prediction, batch correction, *K*/*S*, levelness, fastness, function, energy, water, chemical hazard, fiber strength, handle, effluent quality and cost. The preferred process should not only produce the darkest shade, but also be balanced.

### 9.4 Durability and lifetime assessment

Durability remains the strongest technical barrier. Washing and rubbing fastness are often acceptable, commonly around grade 4 or higher after mordanting, biomordanting, nano-fixation, or surface treatment, whereas light fastness repeatedly remains weak to moderate, commonly ranging from grades 1–2 to 3 for several coffee-, sea-buckthorn-, weld-, alizarin-, curcumin-, and waste-derived systems.^35,37,43,68,72,116,140^ Encouraging cases include chitosan-assisted phenolic systems retaining antibacterial zones after 10 washes, though at reduced levels;^33^ rice-straw-treated nylon retaining UPF and antioxidant activity, with only about 9–18% and 10–15% reductions, respectively, after 20 washes;^20^ Al-post-mordanted luteolin PA56 retaining a UPF of around 155, ABTS scavenging activity above 60%, and 75% antibacterial activity against *S. aureus* after 10 washes;^97^ and industrial madder-dyed polyamide retaining a *K*/*S* of 5.249 after 20 washes.^76^ In contrast, some studies report only initial functional performance, such as >99.9% *S. aureus* reduction and a UPF of 177.53 for spent-coffee-ground nylon, without wash-retention data.^140^ Thus, rice straw, luteolin/Al^3+^ coordination, AgNP-assisted plant systems, and selected industrial trials currently provide stronger functional evidence.^20,76,97,173^ Future work must test repeated laundering, light ageing, rubbing, perspiration, storage, pH cycling, functional retention, and leaching.

### 9.5 Environmental and economic verification

The primary sustainability challenge is the lack of a verified cleaner process in relation to a bio-based source. There are numerous studies reporting beneficial uses of plant waste, microbial pigments, biomordants, or mordant-free methods, but none that include COD, BOD, residual color, aquatic toxicity, solvent residues, metal release, salt load, shedding of nanoparticles or bath biodegradability.^20,33,35,44,57,140,158,173^ There are valuable exceptions, although they are not yet completed, *e.g.*, olive vegetable water dyeing was assessed for polyphenols, COD, and BOD5, but COD remained high after ultrasonic dyeing, at 61.04 g L^−1^.^43^ Likewise, production-level LCA/TEA for biosynthetic indigoids indicated scenario-specific reductions in GWP and water use but did not include any dye-house effluent indicators.^157^ Further studies should incorporate LCA, water footprint, energy inventory, toxicity, metal release, cost/sensitivity analysis, and direct comparison with conventional acid or disperse dyeing. Natural origin alone does not ensure low demand for wastewater treatment, especially with effluents showing very high COD/BOD values.^37,43,123^

### 9.6 Pilot-scale processing and industry compatibility

The majority of evidence can be found in laboratory-scale studies, often in small swatches, high liquor ratios, isolated extracts, or controlled dyepots. Studies using commercial equipment, bath reuse, commercial digital printing, or pilot-scale production are more industrially relevant. The strongest example is industrial polyamide dyeing with catechu, madder, pomegranate peel, and mulberry leaf, where pomegranate peel reached a *K*/*S* of 6.466, madder retained a *K*/*S* of 5.249 after 20 washes, and all samples reached UPFs of 1818–2000, although light fastness remained limited.^76^ Future validations should include jet, package, continuous, padding/steaming, industrial microwave, ultrasound, inkjet printing, commercial washing/finishing, shade matching, bath-circulation control, and low-liquor processing. Recycled nylon, nylon/elastane, and silk/polyamide blends also must be tested.

### 9.7 Staged research roadmap

**Fig. 6:**
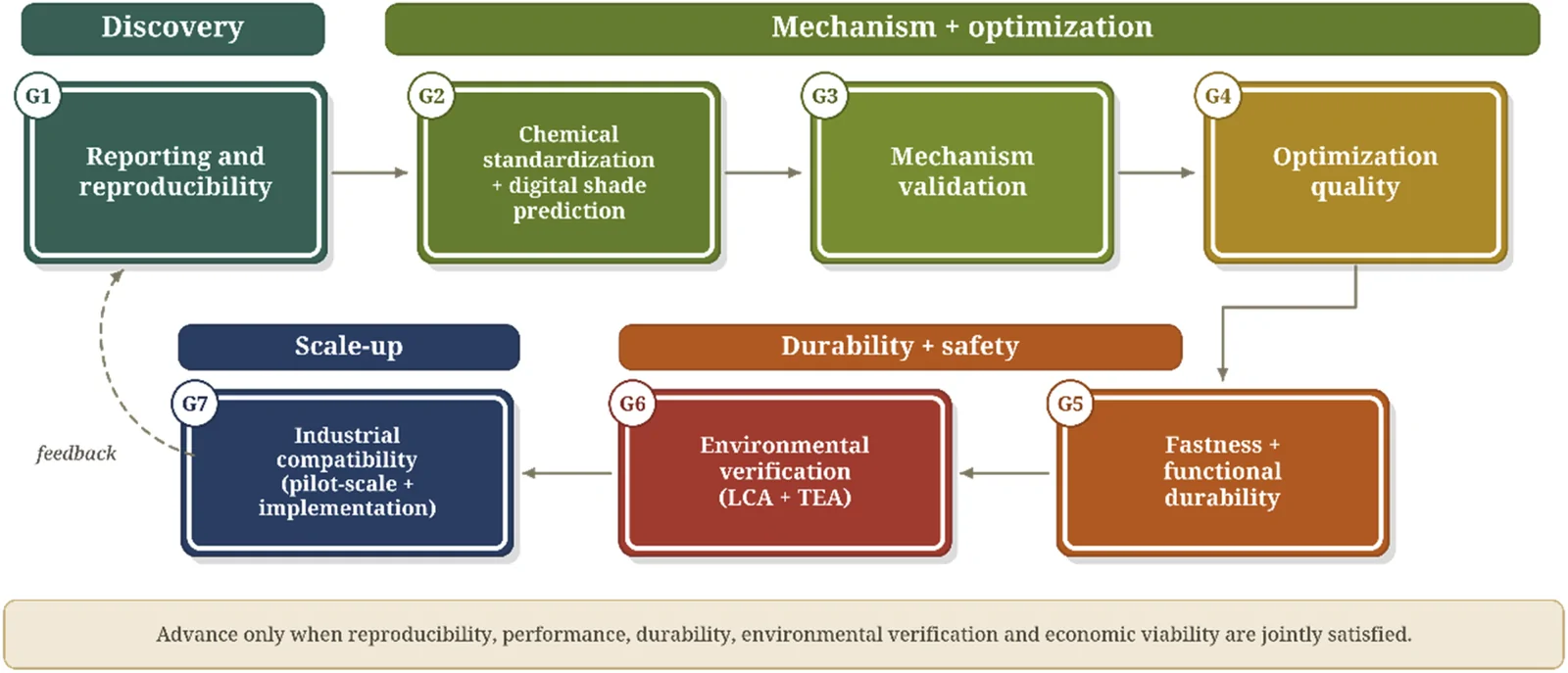
Research and commercialization roadmap for the cleaner natural-dye coloration of nylon.

A staged roadmap should have a series of decision points between discovery and commercialization. The roadmap is organized into seven interconnected stages, labeled G1–G7, to ensure that all its sections are in direct correspondence with the priority research gaps summarized in Table 8. G1, reporting and reproducibility, must include the complete identification of the nylon substrate, the colourant source, processing conditions, test methods, *etc.*, as well as replication and controls, to provide a solid basis for comparison. G2, chemical standardization, should be accompanied by marker-compound or pigment-purity information and dye-liquor fingerprints, and should include digital shade prediction and batch correction to convert variability in extracts and pigments into reproducible CIELAB and *K*/*S* targets. G3, mechanism validation, requires proposed dye–fiber interactions to be verified on the relevant nylon/polyamide substrate rather than extrapolated from wool, silk, cotton, or polyester. G4, optimization quality, should balance color strength, levelness, fastness, energy, water, effluent quality, chemical hazard, and cost rather than maximizing *K*/*S* alone. G5, fastness and functional durability, requires color and functional retention to be confirmed under repeated, and where appropriate, combined washing, rubbing, perspiration, light-ageing, storage, and functional-use conditions. G6, environmental verification, requires effluent quality, toxicity, chemical release, water and energy demand to be assessed alongside life-cycle and techno-economic performance. G7, industrial compatibility, should then validate systems that satisfy the preceding performance, safety, reproducibility, environmental, and economic criteria using pilot-scale equipment, shade-matching and batch-reproducibility trials before progression to industrial implementation. The staged research and commercialization route is presented in Fig. 6, with G1–G7 corresponding directly to the priority research gaps and recommended actions summarized in Table 8, thereby linking each identified evidence gap to its respective stage in the pathway toward commercialization.

**Table 8:** Priority research gaps and future roadmap actions for the cleaner natural-dye coloration of nylon/polyamide textiles

Research gap	Critical evidence from the reviewed literature	Future roadmap action	Ref.
Reporting and reproducibility	Incomplete nylon grade, fabric construction, extract yield, active dye concentration, liquor ratio, replication, and test standards; studies span PA6, PA6,6, PA56, PA5,10, recycled nylon and blends	Adopt a mandatory reporting template and standardized ISO/AATCC/GB testing; include statistics and raw process conditions	20, 33, 35, 57, 76 and 158
Chemical standardization	Crude extracts often contain multiple phenolics, flavonoids, tannins, carotenoids, alkaloids, proteins, or microbial residues; therefore, shade variation is likely	Use UV-vis/HPLC/LC-MS fingerprints, marker-compound quantification, extract-yield data, and batch correction, and digital shade-prediction models linked to CIELAB/*K*/*S* targets	20, 35, 57, 140 and 158
Mechanism validation	Mechanisms are mostly inferred from FTIR/SEM/*K*/*S*; abnormal pH and mordant effects show that ionic bonding alone is insufficient	Combine XPS, zeta potential, desorption, adsorption modeling, surface/depth analysis, and molecular simulation	20, 36, 40, 44, 61 and 79
Optimization quality	Many studies optimize only *K*/*S* or use single-factor designs; acidic pH and high temperature often improve color depth but may increase chemical, water, or energy burdens	Apply multi-objective RSM/AI optimization for *K*/*S*, levelness, fastness, energy, water, hazard, fiber damage, effluent, and cost, plus shade reproducibility across extract batches	60, 92, 102 and 127
Fastness and functional durability	Washing and rubbing fastness are often acceptable, but light fastness is frequently weak; many antibacterial/UV/antioxidant results are initial values only. Light fastness is the most recurring weakness across otherwise promising systems	Test repeated washing, light ageing, rubbing, perspiration, storage, functional retention, and leaching	20, 33, 76, 97, 140 and 167
Environmental verification	Renewable sources are often equated with cleaner dyeing despite missing COD, BOD, residual color, toxicity, solvent and metal-release data	Measure wastewater quality, toxicity, biodegradability, metal/nanoparticle release, LCA, water footprint and TEA	37, 43, 44 and 157
Industrial compatibility	Most work uses laboratory swatches and high liquor ratios; only a few studies include industrial-scale polyamide dyeing or equipment-compatible routes	Validate jet, package, continuous, printing, industrial microwave/ultrasound, and commercial finishing routes. Include shade matching, bath-circulation control, low-liquor processing, and nylon/elastane compatibility	42, 62, 76, 153 and 163

The evidence shows that natural dyes are feasible for nylon, but the most mature candidates differ by end use. Phenolic and tannin-rich waste extracts are promising for brown/yellow-brown shades, UV protection, and circularity when repeated-wash performance is verified.^20,37,76^ Curcumin, baicalin, luteolin, microbial pigments, and AgNP-assisted systems are more promising for functional textiles, but leaching, light stability, and safety need stricter confirmation.^47,97,158,172,173^ Yellow systems such as curcumin, baicalin, luteolin, saffron, weld, and turmeric are best suited to indoor multifunctional or smart textiles unless photostability improves. Red/orange systems such as Brazilwood, cochineal, madder, dragon's blood, and fungal anthraquinones provide attractive shades and sometimes high *K*/*S*, yet light fastness, washing-related color change, or metal-mordant dependence limit their broad use in apparel.^78,98,102,167^ Blue indigoid routes are the strongest nylon blue candidates when reduction chemistry, oxidation control, and spent-bath quality are verified; bio-indigo is especially promising because initial fastness can reach grades 4–5 to 5, but laundering and wastewater validation remain necessary.^34,41,80^ Therefore, cleaner nylon coloration should be judged by quantified color performance, durability, effluent quality, toxicity, resource demand, cost, and dyehouse compatibility, not by natural origin alone.

## 10 Conclusion

This critical review shows that nylon is indeed chemically compatible with several classes of natural colorants, such as tannins, flavonoids, anthraquinones, curcuminoids, indigoids and some microbial pigments. This compatibility is due to the synergistic effects of the terminal amino and carboxyl groups, amide carbonyl and N–H sites, hydrophobic methylene segments, and accessible amorphous regions. Acidic dye baths promote uptake in most anionic phenolic and related systems by protonation of terminal amino groups, thus generating positively charged sites. However, the processing window is not always optimal under acidic conditions and must be determined according to the dye charge, colorant stability, nylon grade, and potential for fiber damage.

The evidence also shows that the performance of the coloration depends not only on the chemical affinity, but also on the diffusion and fixation processes. Color strength and resistance to washing and rubbing are influenced by nylon crystallinity and free volume, textile construction, processing temperature and time, liquor ratio, pretreatment, and colorant aggregation. Low-crystallinity polyamides may thus have higher dye uptake than more compact grades, while higher temperature or acidity can result in higher apparent color strength but may cause losses in fiber integrity or pigment stability. Microwave, ultrasound, plasma, and enzymatic processing can be used to promote the extraction process, enhance mass transfer, activate the fiber surface, or achieve milder coloration. However, these approaches are not necessarily considered cleaner simply because they reduce the treatment time or remove a processing step, as their environmental impact is related to the specific energy consumption, the consumption of solvents and auxiliary materials, the efficiency of the equipment, fiber damage, and scalability.

The adsorption isotherms and kinetic models are usually fitted with reasonable statistical precision, and the mechanistic meaning of the fitted models is then often exaggerated. Agreement with Langmuir, Freundlich, pseudo-first-order or pseudo-second-order models does not imply monolayer adsorption, chemisorption, or the presence of a particular dye–fiber bond. Direct molecular characterization, mass-balance analysis, desorption experiments, and validation of dye location within the polyamide structure are needed to strengthen the conclusions.

Light fastness, batch-to-batch reproducibility, wet durability properties, and the retention of antibacterial, antioxidant, UV protection, or sensing properties after repeated use are still major limitations, despite encouraging color strength and multifunctionality. Metallic mordants can be advantageous in increasing shade depth and durability; however, some metallic mordants, including chromium, copper, tin, and other metals, can directly oppose the aims of cleaner production due to toxicity, effluent contamination, and release during laundering. Overall, coloration evidence is much stronger than environmental, economic, and scale-up evidence.

Chemically standardized extracts, clearly identified nylon substrates, harmonized testing, repeated laundering and light-ageing studies, complete water-energy-chemical inventories, effluent and toxicity assessment, life-cycle and cost analyses and pilot trials with industrial equipment are needed for future progress. The field now needs to shift its focus from demonstrating that nylon can be naturally dyed to demonstrating that it can be colored reproducibly, durably, safely and competitively at scale.

## Author contributions

MAC: conceptualization, investigation, data curation, formal analysis, writing – original draft, writing – review & editing. MMA: investigation, formal analysis, validation, writing – review & editing. NA: investigation, data curation, formal analysis, visualization, writing – original draft, writing – review & editing. MSI: supervision, validation, formal analysis, writing – review & editing. BCM: conceptualization, methodology, supervision, validation, formal analysis, visualization, project administration, writing – original draft, writing – review & editing. All authors reviewed, revised, and approved the final version of the manuscript.

## Conflicts of interest

There are no conflicts to declare.

## Supplementary Material

RA-OLF-D6RA07080A-s001

RA-OLF-D6RA07080A-s002

## Data Availability

No primary experimental data were generated. The literature data supporting this review are available within the article and its cited references. Supplementary information (SI) is available. See DOI: https://doi.org/10.1039/d6ra07080a.
