# Supplementary material for: Coloration of nylon textiles with natural dyes: a critical review of recent progress, dye–fiber interactions, fastness, and functional performance
Source: RSC Adv. 2026 Sep 25;16(45):56366–415. doi: 10.1039/d6ra07080a (PMC13613645; doi:10.1039/d6ra07080a)
Supplement: Supplementary file 1 [file RA-016-D6RA07080A-s001.pdf]

Supplementary Information (SI) for RSC Advances.
This journal is © The Royal Society of Chemistry

Supplementary Methods and Source Data for Figure 4

Cleaner Coloration of Nylon Textiles with Natural Dyes: A Critical Review of Recent Progress, Dye-Fiber Interactions, Fastness, and Functional Performance

Muksit Ahamed Chowdhury [1], Mohammad Mahbubul Alam [1], Nayeem Ahmed [1], Md. Shahinoor Islam [3], and Badhon C. Mazumder [2*]

Supplementary Note S1. Scope and data provenance

This supplementary document provides the complete scoring rules, category definitions, evidence-strength calculation, and final plotted coordinates used to construct Figure 4. The map is based on a transparent semi-quantitative multi-criteria model rather than visual judgment.

The representative operating conditions, K/S or exhaustion values, fastness results, levelness observations, fiber-integrity observations, and evidence counts were taken from the studies synthesized in Section 5 of the main manuscript. The indices are dimensionless and are intended for comparative interpretation within this review.

Supplementary Table S1. Symbols and abbreviations used in the Figure 4 scoring framework.

Symbol	Definition
PSI_j	Process Severity Index for route j (0-100)
PPI_j	Coloration and durability Performance Index for route j (0-100)
ESI_j	Evidence Strength Index for route j (0-100)
T_j*	Normalized representative process-temperature score
t_j*	Normalized representative treatment-time score
LR_j*	Normalized material-to-liquor-ratio score
C_j*	Chemical-burden score
E_j*	Equipment-burden score
CR_j	Color-response score
F_j	Fastness score
L_j	Levelness score
I_j	Fiber-integrity score
n_j	Number of distinct supporting studies for route j
n_max	Maximum supporting-study count across routes (17)
A_j	Bubble plotting area used for route j

Supplementary Note S2. Process Severity Index

The horizontal coordinate was calculated as:

$$PSI_j = 20(T_j^* + t_j^* + LR_j^* + C_j^* + E_j^*) \quad (S1)$$

PSI ranges from 0 to 100. Temperature, time, water demand, chemical burden, and equipment burden were given equal weights of 20% because the reviewed literature did not provide a defensible basis for prioritizing one criterion over another.

S2.1. Normalized quantitative criteria

$$T_j^* = \min[1, \max(0, (T_j - 25)/(120 - 25))] \quad (S2)$$

T_j is the representative process temperature in degrees Celsius. The lower and upper limits represent ambient processing and the approximate upper temperature used by the reviewed intensified nylon systems.

$$t_j^* = \min(1, t_j/240) \quad (S3)$$

t_j is treatment time in minutes. Values above 240 min were capped at 1 so that the 24 h microbial process did not dominate the axis.

$$LR_j^* = \min(1, LR_j/50) \quad (S4)$$

LR_j is the material-to-liquor ratio expressed as liters per kilogram or the equivalent numerical ratio. Zero-water routes were assigned $LR_j = 0$; ratios of 1:50 or higher received the maximum water score.

S2.2. Chemical-burden score

Supplementary Table S2. Predetermined chemical-burden scoring rules (C_j^*).

Score	Definition
0.00	No fixation chemical or dry/CO_2-based treatment
0.50	pH adjustment, enzyme/H_2O_2, bio-based auxiliary, or relatively mild chemical assistance
1.00	Metallic mordant, nanoparticle system, deep eutectic solvent, organic carrier, or strong reduction/fixation chemistry

S2.3. Equipment-burden score

Supplementary Table S3. Predetermined equipment-burden scoring rules (E_j^*).

Score	Definition
0.00	Conventional dyeing or fixation equipment
0.50	Microwave, ultrasound, atmospheric plasma, bioreactor, digital system, or nanoparticle-preparation equipment
1.00	High-pressure supercritical equipment or ionizing-radiation system

Chemical- and equipment-burden values were assigned using the same predetermined rules for every route; they were not adjusted according to whether a route appeared cleaner or more favorable.

Supplementary Note S3. Coloration and durability Performance Index

The vertical coordinate was calculated as:

$$PPI_j = 25(CR_j + F_j + L_j + I_j) \quad (S5)$$

Each criterion contributes 25%, giving a final PPI of 0-100. Color strength and exhaustion were not combined as raw values because the included studies used different colorants, wavelengths, substrates, and measurement conditions.

S3.1. Color-response score

Supplementary Table S4. Color-response scoring rules (CR_j).

Score	Reported color response
0.25	K/S < 5 or exhaustion < 60%
0.50	K/S 5 to <10 or exhaustion 60% to <75%
0.75	K/S 10 to <15 or exhaustion 75% to <85%
1.00	K/S >= 15 or exhaustion >= 85%

Illustrative route-level assignments reported in the source dataset were: microwave-assisted dyeing, 1.00 (93% exhaustion); ultrasound-assisted dyeing, 0.75 (81% exhaustion); plasma activation, 0.75 (K/S = 10.7); and enzymatic coloration, 0.25 (K/S approximately 4.5).

S3.2. Fastness score

Supplementary Table S5. Fastness scoring rules (F_j).

Score	Overall durability interpretation
0.25	Poor fastness, generally <=2
0.50	Fair, variable, or incompletely reported performance
0.75	Good performance, generally around grade 3-4 or equivalent
1.00	Very good/excellent performance, generally >=4-5 or high blue-wool ratings

Washing, rubbing, and light fastness were considered together. A route was not assigned the highest score solely because one fastness property was high.

S3.3. Levelness score

Supplementary Table S6. Levelness scoring rules (L_j).

Score	Interpretation
0.25	Clearly poor or uneven coloration
0.50	Mixed or not adequately quantified
0.75	Generally acceptable levelness
1.00	Explicitly reported high levelness or highly controlled local deposition

S3.4. Fiber-integrity score

Supplementary Table S7. Fiber-integrity scoring rules (I_j).

Score	Interpretation
0.25	Clear melting, cracking, hardening, or major strength loss
0.50	Integrity not adequately quantified
0.75	Minor strength loss of approximately <=10% or limited damage risk
1.00	Negligible damage or explicitly demonstrated fiber preservation

A score of 0.50 was used as a neutral missing-information value. Missing information was therefore treated as neither successful nor failed performance.

Supplementary Note S4. Evidence Strength Index and bubble area

Bubble size was calculated from the number of distinct supporting studies:

$$ESI_j = 100 \times \ln(1 + n_j) / \ln(1 + n_{max}) \quad \text{(S6)}$$

Here, n_j is the number of supporting studies for route j and n_{max} = 17, corresponding to biomordanting. The logarithmic transformation prevents heavily studied routes from visually overwhelming routes supported by fewer studies.

The bubble plotting area was calculated as:

$$A_j = 80 + 9(ESI_j) \quad \text{(S7)}$$

A_j affects only bubble size. It does not alter the route's x-coordinate or y-coordinate.

Supplementary Table S8. Final route coordinates and bubble areas used in Figure 4

PSI and PPI are the plotted coordinates. ESI and A_j determine the relative bubble size. Values are reported to two decimal places.

Code	Processing route	PSI (x)	PPI (y)	Studies (n)	ESI	Bubble area A_j	PPI - PSI
CED	Conventional exhaust dyeing	42.06	56.25	8	76.02	764.18	14.19
MF	Mordant-free dyeing	40.68	87.50	7	71.94	727.46	46.82
MM	Metallic mordanting	50.68	81.25	14	93.69	923.21	30.57
BM	Biomordanting	43.18	87.50	17	100.00	980.00	44.32
DLL	Digital/low-liquor systems	38.08	75.00	5	61.99	637.91	36.92
MW	Microwave-assisted dyeing	49.02	81.25	7	71.94	727.46	32.23
US	Ultrasound-assisted dyeing	52.17	75.00	5	61.99	637.91	22.83
PL	Plasma activation	10.83	81.25	4	55.68	581.12	70.42
LE	Laser/electron-beam activation	21.89	81.25	2	38.01	422.09	59.36
ENZ	Enzymatic coloration	53.32	56.25	3	47.96	511.64	2.93
MIC	Microbial in situ coloration	49.05	62.50	3	47.96	511.64	13.45
NF	Nanoformulation	52.47	62.50	3	47.96	511.64	10.03
DES	Deep eutectic solvent	42.37	56.25	1	23.98	295.82	13.88
SC	Supercritical CO_2	43.95	56.25	2	38.01	422.09	12.30

The final column is a derived balance value. Positive values indicate that PPI exceeds PSI; this is equivalent to a point lying above the PPI = PSI reference line.

Supplementary Note S5. Interpretation and limitations

Plasma and laser/electron-beam activation: These routes occupy the upper-left region because they combine comparatively low water and thermal demands with high reported performance. Their smaller bubbles indicate that the conclusion is supported by fewer studies.

Biomordanting and mordant-free dyeing: These routes have the highest calculated PPI values. Metallic mordanting also performs strongly, but its PSI is higher because metal-based chemical burden is explicitly included.

Conventional exhaust dyeing: This route has a mature evidence base but a moderate PPI because performance varies among dyes and often depends on substantial water, heat, and afterwashing.

The reference line used in the figure is:

$$PPI = PSI \tag{S8}$$

Points above the line have a higher performance score than process-severity score. The line is a visual reference and not a statistical regression.

S5.1. Methodological limitations

- The framework is semi-quantitative and depends on route-level representative values synthesized from heterogeneous studies.
- Equal weighting was used because the available literature did not support an alternative weighting hierarchy.
- K/S and exhaustion were converted to fixed bands rather than pooled as directly comparable continuous outcomes.
- A neutral score of 0.50 was assigned when a performance criterion was inadequately reported.
- ESI reflects the number of supporting studies, not study quality, sample size, or certainty of effect.
- The indices are intended for comparison within this review and should not be interpreted as life-cycle assessment results.

Data availability statement for the supplementary file

All scoring equations, categorical thresholds, final route coordinates, evidence counts, and derived bubble areas used to construct Figure 4 are provided in this supplementary document. The underlying studies and route-level evidence are cited and discussed in Section 5 of the main manuscript.
