# Supplementary material for: Coloration of nylon textiles with natural dyes: a critical review of recent progress, dye–fiber interactions, fastness, and functional performance
Source: RSC Adv. 2026 Sep 25;16(45):56366–415. doi: 10.1039/d6ra07080a (PMC13613645; doi:10.1039/d6ra07080a)
Supplement: Supplementary file 2 [file RA-016-D6RA07080A-s002.pdf]

Supplementary Information (SI) for RSC Advances.
This journal is © The Royal Society of Chemistry

SUPPLEMENTARY INFORMATION

Cleaner Coloration of Nylon Textiles with Natural Dyes: A Critical Review of Recent Progress, Dye-Fiber Interactions, Fastness, and Functional Performance

Scope and provenance. This table lists the 158 unique strong-match articles recorded in the deduplicated Scopus and Web of Science screening workbook. Records are presented in the source workbook order and retain the bibliographic and screening terminology recorded there. Blank metadata fields are shown as "Not reported in screening workbook." The table documents the article inventory considered for review synthesis; final inclusion status should remain consistent with the manuscript and study-selection workflow.

Unique articles	158	Both databases	83	Scopus only	56	Web of Science only	19
Deduplication basis recorded in the source workbook: exact DOI match, followed by exact normalized-title match, followed by high title-similarity matching across Scopus and Web of Science.							

Supplementary Table S1. Full article inventory and key bibliographic, database, colorant-source, and nylon/polyamide-substrate metadata.

ID	Year	Article title	Authors	Source / DOI	Database coverage	Natural dye or source	Nylon / polyamide substrate
1	2026	Biocatalytic oxidative coloration of bio-based Nylon 5,10 using natural phenolics	Baek, N.-W.	*Enzyme and Microbial Technology* 10.1016/j.enzmictec.2026.110858	Both databases	Natural/bio-derived colorant not specified in title/abstract	nylon 6; nylon; polyamide
2	2026	Changes in the Chemical and Physical Properties of Untreated and Finished Polyamide 6.6 Fabrics Buried in Different Soil Matrices, from the Lab-Scale to a House Garden	Piccioni, M; Peila, R; Tummino, ML	*SUSTAINABLE CHEMISTRY* 10.3390/suschem7010013	Web of Science only	Not reported in screening workbook	Not reported in screening workbook
3	2026	Chitosan-templated enzymatic oxidative polymerization of phenolic pigments on bio-based polyamide 5,10	Baek, NW; Lee, SH	*COLLOIDS AND SURFACES A-PHYSICOCHEMICAL AND ENGINEERING ASPECTS* 10.1016/j.colsurfa.2026.140651	Web of Science only	Not reported in screening workbook	Not reported in screening workbook
4	2026	Colorimetric properties of Brazilwood-dyed textiles under various dyeing conditions	Park, Y.K.; Kang, D.; Cho, H.S.; Choi, H.Y.; Lee, J.S.	*Journal of the Textile Institute* 10.1080/00405000.2026.2639062	Both databases	Brazilwood	nylon
5	2026	Eco-friendly development of multi-functional textiles using rice straw extract	Abou-Taleb, M.; Zaher, K.S.A.; Mowafi, S.; Nawwar, G.A.M.	*Scientific Reports* 10.1038/s41598-026-43684-5	Both databases	rice straw extract	nylon 6; nylon
6	2025	Effect of soy milk pre-treatment on the dyeability of polyamide with annatto dye	Francisco, I.B.M.; Silva de Oliveira, C.R.; da Silva Júnior, A.H.; de Aguiar, C.R.L.	*Pigment and Resin Technology* 10.1108/PRT-07-2025-0072	Both databases	annatto/norbixin/bixin; soy milk pre-treatment; carotenoids/tomato waste	polyamide
7	2026	Engineering artificial biosynthetic pathway enables simultaneous production and in-situ bio-dyeing of indigoids for textiles	Na, Q.; Zhang, X.; Long, L.; Qi, S.; Zhang, Z.; Zhi, R.; Tan, Z.; Ying, H.; Zhu, C.	*Nature Communications* 10.1038/s41467-025-67935-7	Scopus only	Not reported in screening workbook	nylon
8	2026	Enhancing the fastness characteristics of weld-dyed cotton and recycled nylon 6,6 fabrics via sodium alginate and titanium dioxide	Karadag, R.; Sagirli, F.Z.E.; Pars, A.	*Coloration Technology* 10.1111/cote.70071	Both databases	weld/Reseda luteola	nylon 6,6 / nylon 66; nylon 6; nylon
9	2026	Intense and vibrant color construction and functionalization for protein macromolecule/polyamide two-component fabric by sustainable microbial nano prodigiosins based on adjustable pigment allocation	Zheng, Q.; Fu, R.; Du, X.; Wu, J.; Luo, A.; Ren, Y.	*International Journal of Biological Macromolecules* 10.1016/j.ijbiomac.2026.151986	Scopus only	prodigiosin	polyamide; silk/polyamide blend
10	2026	Multifunctional modification of bio-based polyamide 56 fabric via luteolin for durable colouristic, antioxidant, antibacterial and UV protective properties	Zhang, X; Li, MY; Zhang, X; Hou, XN; Wang, XR	*SUSTAINABLE CHEMISTRY AND PHARMACY* 10.1016/j.scp.2025.102295	Web of Science only	Not reported in screening workbook	Not reported in screening workbook

11	2026	Oleaeuropea (olive leaf) extract as a sustainable material for enhancing some appearance and performance properties of polyamide 6 fabric	Hassan, H.; Mowafi, S.; Bakry, M.; El-Sayed, H.	*Journal of Adhesion Science and Technology* 10.1080/01694243.2025.2573639	Both databases	olive leaf/vegetable water	polyamide 6 / PA6; polyamide
12	2025	Pollution-free and sustainable coloration of nylon/cotton blended textiles with natural colorant	Rehman, A.; Naveed, M.; Iqbal, K.; Siyal, M.I.	*Pigment and Resin Technology* 10.1108/PRT-02-2025-0013	Both databases	Natural/bio-derived colorant not specified in title/abstract	nylon; nylon/cotton blend
13	2026	Research on overdyeing process of indigo and turmeric on nylon knitted fabrics	Wu, Y.; Yuan, X.; Chen, X.; Li, X.; Shi, X.; Dong, F.; Zhang, J.	*Scientific Reports* 10.1038/s41598-026-45744-2	Both databases	turmeric/curcumin	nylon
14	2026	Sustainable Approach to Mordant-Free Natural Dyeing of Nylon Fabric with Neem Leaf Extract	Azom, M.G.; Uddin, M.Z.	*Textile and Leather Review* 10.31881/TLR.2026.100	Scopus only	neem leaf	nylon
15	2026	Sustainable multifunctional dyeing of nylon with Babool (Acacia nilotica) bark extract: Enhancing color metrics, fabric performance, UV protection, and antibacterial activity	Sarker, P.; Rahaman, M.T.; Al Mamun, M.A.; Liza, U.A.; Khan, M.A.; Hossain, M.M.	*Hybrid Advances* 10.1016/j.hybadv.2026.100599	Scopus only	babool/Acacia nilotica	nylon
16	2025	Application of different concentrations of the natural dye potassium norbixinate (annatto) in polyamide 6.6 fabrics	Gonçalves, M.J.; de Oliveira, A.C.V.; Colla Prando, A.; Krebs de Souza, C.; Siqueira Curto Valle, R.D.C.; Barcellos, I.O.; Tavares, L.B.B.	*Natural Product Research* 10.1080/14786419.2023.2280178	Both databases	annatto/norbixin/bixin	polyamide 6,6 / PA66; polyamide 6 / PA6; polyamide
17	2025	Bio- colouration of nylon fabric using natural dyes and mordants	Chowdhury, T.A.; Khandaker, J.I.; Gafur, M.A.; Repon, M.R.; Hossain, A.; Islam, M.K.; Shanto, N.K.	*Materials Research Innovations* 10.1080/14328917.2024.2386193	Scopus only	neem leaf	nylon
18	2025	A comparative study on recovery and recycle of acid dye and silk fibroin from colored waste silk textiles through dye stripping and silk dissolution	Fan, S; Rather, LJ; Pu, JY; Li, Q	*SUSTAINABLE CHEMISTRY AND PHARMACY* 10.1016/j.scp.2025.102090	Web of Science only	Not reported in screening workbook	Not reported in screening workbook
19	2025	Curcumin Nanoparticles as a Pigment of Polyamide Textiles: Influence of Conditions in the Exhaust Dyeing and Evaluation of Wastewater Toxicity	Moraes, K.S.; de Oliveira, A.; Ferreira, B.T.M.; Bona, E.; Carneiro, J.; Peron, A.P.; Fernandes, M.; Zille, A.; Leimann, F.V.; Lange de Aguiar, C.R.; da Silva, M.G.; Ferreira, A.J.S.; Vila, N.T.; Hess Gonçalves, O.	*ACS Sustainable Chemistry and Engineering* 10.1021/acssuschemeng.5c06849	Both databases	turmeric/curcumin	polyamide 6,6 / PA66; polyamide 6 / PA6; polyamide
20	2025	Development of Antibacterial and Halochromic Textiles by Modification of Emodin from Japanese Knotweed	Avbelj, M.; Zagorec, M.H.; Petković, H.; Gorjanc, M.; Iskra, J.	*Journal of Natural Fibers* 10.1080/15440478.2024.2445579	Both databases	emodin/dermocybin	polyamide
21	2025	Dyeing Characteristics and Performance of Natural Dye derived from Spent Coffee Grounds on Cotton, Silk and Nylon Fabrics using Eco-friendly Dyeing Process	Setthayanond, J.; Tooptompong, P.; Suwanruji, P.; Srisuwan, P.	*Chiang Mai Journal of Science* 10.12982/CMJS.2025.002	Both databases	spent coffee/coffee pomace	nylon
22	2025	Dyeing Properties and Skin Health Functions of Cotton and Nylon/PU Knits Dyed with Sea Buckthorn Leaves	Badmaanyambuu, S.; Yi, E.	*Journal of the Korean Society of Clothing and Textiles* 10.5850/JKSCT.2025.49.2.419	Scopus only	sea buckthorn	nylon
23	2025	Eco-friendly approach of bio-indigo synthesis and developing purification methods towards isolation of indigo from indirubin and bacterial fragments	Manivannan, R.; Prabakaran, K.; Son, Y.-A.	*Sustainable Chemistry and Pharmacy* 10.1016/j.scp.2025.101954	Both databases	Natural/bio-derived colorant not specified in title/abstract	nylon
24	2025	Eco-friendly Dyeing Optimization of Wool and Polyamide Fabrics with Ground Coffee Pomace: a Box-Behnken Design Approach	Khedher, F.; Jaouachi, B.	*Fibers and Polymers* 10.1007/s12221-025-01184-9	Both databases	spent coffee/coffee pomace; lignin nanoparticles	polyamide
25	2025	Eco-Friendly Extraction of Curcumin from Turmeric and Dyeability of Textile Fibers	Popescu, V.; Alexandrescu, A.-D.; Popescu, G.; Vasilache, V.	*Fibers* 10.3390/fib13060073	Both databases	turmeric/curcumin	polyamide

26	2025	Efficient production of lignin nanoparticle colloids and their feasibility for eco-friendly dyeing of natural and synthetic textile fabrics	Pereira, P.H.F.; Costa, S.A.; Costa, S.M.; Arantes, V.	*Industrial Crops and Products* 10.1016/j.indcrop.2024.120390	Both databases	lignin nanoparticles	polyamide
27	2025	Exploration of the natural dye alizarin for supercritical carbon dioxide dyeing of polyester, polyamide and cellulose acetate fibres	Schmidt, M.; Falkowski, R.; Räisänen, R.; Williams Cherry, T.N.	*Coloration Technology* 10.1111/cote.70000	Both databases	madder/alizarin/purpurin	polyamide
28	2025	Extraction, characterization, and application of a polyphenolic compound as a natural dye for polyamide fabrics	Otutu, J.O.; Clark, P.D.; Osharode, M.E.; Otutu, E.C.; Ndukwe, G.I.	*Journal of Chemistry Letters* 10.22034/jchemlett.2024.474301.1221	Scopus only	Natural/bio-derived colorant not specified in title/abstract	polyamide
29	2025	Green extraction of colorants from achiote seeds for dyeing mordanted nylon fabric	Adeel, S.; Ur-Rehman, F.; Haji, A.; Tahir, M.A.; Mia, R.; Imran, M.	*Surface Innovations* 10.1680/jsuin.24.00073	Both databases	annatto/norbixin/bixin; pomegranate peel/rind	nylon
30	2025	Green synthesis of silver nanoparticles on polyamide fabrics using Scrophularia striata Boiss extract: Characterization, dyeing, and antibacterial properties	Sadeghi-Kiakhani, M.; Hashemi, E.; Norouzi, M.-M.; Soleimani, P.; Babaahmadi, V.	*Journal of Industrial and Engineering Chemistry* 10.1016/j.jiec.2024.10.069	Both databases	Natural/bio-derived colorant not specified in title/abstract	polyamide
31	2025	Harnessing soybean okara for enhanced microbial production of indigoidine	Chen, Z.; Liu, W.; Zhang, S.; Song, Y.; Zhang, Z.; Yao, G.; Zhao, G.-R.; Zhu, H.; Zhang, D.; Qiao, J.; Zhao, H.; Liu, J.	*Bioresource Technology* 10.1016/j.biortech.2025.132808	Scopus only	Natural/bio-derived colorant not specified in title/abstract	nylon
32	2025	Insight into the Dyeability of Bio-Based Polyamide 56 by Natural Dyes	Sun, C.; Wu, J.; Wang, Y.	*Chemistry (Switzerland)* 10.3390/chemistry7030095	Both databases	turmeric/curcumin; madder/alizarin/purpurin; catechu/Areca catechu	polyamide 6,6 / PA66; polyamide 6 / PA6; polyamide
33	2025	Investigation into the Application of Natural Dyes Obtained from Annatto Seeds and Eucalyptus Leaves in Dyeing Textile Substrates Using Biomordants	Barbosa, L.D.S.; Alves, S.H.; Felipe, B.H.S.; de Oliveira, C.R.S.; de Aguiar, C.R.L.	*Colorants* 10.3390/colorants4030026	Scopus only	annatto/norbixin/bixin; eucalyptus; soy milk pre-treatment	polyamide
34	2025	Investigation of mordant application in direct dyeing of nylon fabrics	Baran, MA; Becerir, B	*TEXTILE RESEARCH JOURNAL* 10.1177/00405175251333663	Web of Science only	Not reported in screening workbook	Not reported in screening workbook
35	2025	Isolation and characterization of natural dyes from Persea americana leaves and their application on polyamide fabrics	Clark, PD; Otutu, JO; Asiagwu, KA; Ndukwe, GI; Otutu, EC; Idibie, CA	*OVIDIUS UNIVERSITY ANNALS OF CHEMISTRY* 10.2478/auoc-2025-0011	Web of Science only	Not reported in screening workbook	Not reported in screening workbook
36	2025	New insights into the role of color extraction from (Aegle Marmelos leaf) using a non-traditional heating source	Sobh, N.; Elshemy, N.; Nassar, S.; Ali, M.	*Pigment and Resin Technology* 10.1108/PRT-05-2023-0041	Scopus only	Extract-based colorant/finish	polyamide
37	2025	Optimizing the eco-friendly dyeing of wool and nylon fabrics with Prangos ferulacea (L.) Lindl using artificial intelligence	Shokrzadeh, S.; Razbin, M.; Ghaheh, F.S.; Tehrani, M.; Sadrjahani, M.	*Scientific Reports* 10.1038/s41598-025-97551-w	Both databases	Prangos ferulacea	nylon
38	2025	Recyclable, eco-friendly dyeing of silk/polyamide two-component fabric using sustainable Monascus pigments based on a nano suspension system	Wang, Y.; Wu, L.; Wu, J.; Zheng, Q.; Fu, R.; Ren, Y.	*International Journal of Biological Macromolecules* 10.1016/j.ijbiomac.2025.141265	Both databases	Monascus pigments	polyamide; silk/polyamide blend
39	2025	Sustainable Eco-Dyeing of Nylon 6 Using Papaver Rhoeas Flower Extract: Optimization and Application of Natural Dyes with Biomordants	Shahmoradi Ghaheh, F.; Haji, A.; Tavakkol, E.; Repon, M.R.	*ChemistrySelect* 10.1002/slct.202405210	Both databases	walnut rind/shell; Papaver rhoeas	nylon 6; nylon
40	2025	Valorisation of Waste kapok leaves in Functional dyeing of nylon fabric	Azeem, M.; Shaiwale, N.; Sheikh, J.	*Sustainable Chemistry One World* 10.1016/j.scowo.2025.100095	Scopus only	kapok leaves	nylon
41	2024	Adsorption, kinetic, and thermodynamic studies of natural curcumin dye on cotton and polyamide fabric and the liberation of its active principle	Schmidt, M.; Bierhalz, A.C.K.; de Aguiar, C.R.L.	*Canadian Journal of Chemical Engineering* 10.1002/cjce.25277	Both databases	turmeric/curcumin	polyamide
42	2024	Bio Dyeing of Nylon 6 Fabrics Using Beetroot and Natural	Shahmoradi Ghaheh, F.; Haji, A.;	*ChemistrySelect*	Both databases	walnut rind/shell;	nylon 6; nylon

		Mordants	Daneshvar, E.; Repon, M.R.	10.1002/slct.202402783		pomegranate peel/rind; beetroot; Terminalia/Thespesia	
43	2024	Clean synthesis of silver nanoparticles (AgNPs) on polyamide fabrics by Verbascum thapsus L. (mullein) extract: characterization, colorimetric, antibacterial, and colorfastness studies	Sadeghi-Kiakhani, M.; Hashemi, E.; Norouzi, M.-M.	*Environmental Science and Pollution Research* 10.1007/s11356-024-33373-z	Scopus only	Verbascum/mullein	nylon; polyamide
44	2024	Eco-Friendly Dyeing Processes of Nylon 6.6 Woven Fabrics with Used Coffee Grounds (UCG)	Becker, P.; Howarth, S.; Ciesielska-Wrobel, I.	*Sustainability (Switzerland)* 10.3390/su16208919	Both databases	spent coffee/coffee pomace	nylon 6,6 / nylon 66; nylon 6; nylon
45	2024	Environmentally Friendly Coloration of Polyamide Fabrics with the Use of Natural Dyes: A Study Including Results of Industrial Scale Applications	Atav, R.; Soysal, S.; Hajı, A.	*Fibers and Polymers* 10.1007/s12221-024-00576-7	Both databases	madder/alizarin/purpurin; catechu/Areca catechu; pomegranate peel/rind	nylon; polyamide
46	2024	Exploring the Potential of Dacryodes Edulis Leaf Extract as Natural Colourant on Polyamide Fabrics: Extraction, Characterization and Application	Clark, P.D.; Otutu, J.O.; Asiagwu, A.K.; Ndukwe, G.	*Substantia* 10.36253/Substantia-2604	Scopus only	Dacryodes edulis	polyamide
47	2024	An Investigation of Verbascum thapsus Flower as a Natural Source for Nylon 6 Fabric Dyeing	Shahmoradi Ghaheh, F.; Haji, A.; Tavakkol, E.	*Fibers and Polymers* 10.1007/s12221-024-00541-4	Both databases	pomegranate peel/rind; Verbascum/mullein; Terminalia/Thespesia	nylon 6; nylon; polyamide
48	2024	Physicochemical Properties of Warp Knitted Fabrics Colored with Madder and Gall Oak for Sportswear	Karadag, R.; Pars, A.	*Journal of Natural Fibers* 10.1080/15440478.2024.2378854	Both databases	madder/alizarin/purpurin	polyamide; polyamide/elastane blend
49	2023	Dyeing Improvement and Stability of Antibacterial Properties in Chitosan-Modified Cotton and Polyamide 6,6 Fabrics	Piccioni, M.; Peila, R.; Varesano, A.; Vineis, C.	*Journal of Functional Biomaterials* 10.3390/jfb14100524	Both databases	cochineal/carmine	polyamide 6,6 / PA66; polyamide 6 / PA6; polyamide
50	2023	Dyeing of Nylon Fabric with Two Natural Dyes, Saffron (Crocus sativus L.) and Weld (Reseda luteola L.), and Study Their Dyeing, Antioxidant, and Antibacterial Properties	Sadeghi-Kiakhani, M.; Hashemi, E.; Miri, F.S.; Tehrani-Bagha, A.R.; Etezad, S.M.	*Fibers and Polymers* 10.1007/s12221-023-00040-y	Both databases	weld/Reseda luteola; saffron/Crocus sativus	nylon
51	2023	Dyeing of polyamide 6 fabric with new bio-colorant and bio-mordants	Haji, A.; Shahmoradi Ghaheh, F.; Mohammadi, L.	*Environmental Science and Pollution Research* 10.1007/s11356-022-24862-0	Both databases	pomegranate peel/rind; dragon's blood resin	nylon; polyamide 6 / PA6; polyamide
52	2023	Ecofriendly Dyeing of Textile Materials with Natural Colorants from Date Palm Fiber Fibrillium	Baaka, N.; Khiari, R.; Haji, A.	*Sustainability (Switzerland)* 10.3390/su15021688	Scopus only	date palm fibrillium	nylon
53	2023	Study of the dyeing properties of saffron and ultrafiltrated saffron powders, as colourants for natural and synthetic fibres	Naidis, P.; Lykidou, S.; Mischopoulou, M.; Vouvoudi, E.; Nikolaidis, N.F.	*Coloration Technology* 10.1111/cote.12670	Both databases	saffron/Crocus sativus	nylon
54	2023	The Sustainable Bioactive Dyeing of Textiles: A Novel Strategy Using Bacterial Pigments, Natural Antibacterial Ingredients, and Deep Eutectic Solvents	Mouro, C.; Gomes, A.P.; Costa, R.V.; Moghtader, F.; Gouveia, I.C.	*Gels* 10.3390/gels9100800	Both databases	prodigiosin; lignin nanoparticles	nylon
55	2023	Sustainable Dyeing Process for Nylon 6 Fabrics by Rhubarb Flower Using Different Bio-Mordants	Shahmoradi Ghaheh, F.; Haji, A.; Daneshvar, E.	*Sustainability (Switzerland)* 10.3390/su15129232	Both databases	spent coffee/coffee pomace; walnut rind/shell; rhubarb/Rheum	nylon 6; nylon
56	2023	Waterless dyeing with plant-based natural dyes as colorants	Freeman, H.S.; Räisänen, R.	*Acta Horticulturae* 10.17660/ActaHortic.2023.1361.16	Both databases	madder/alizarin/purpurin; emodin/dermocybin; weld/Reseda luteola	nylon
57	2022	Agricultural waste of Ipomoea batatas leaves as a source of natural dye for green coloration and bio-functional finishing for textile fabrics	Fang, J.; Meng, C.; Zhang, G.	*Industrial Crops and Products* 10.1016/j.indcrop.2021.114440	Both databases	Ipomoea batatas leaves	nylon

58	2022	Deposition of Silver Nanoparticles on Nylon Fabric and Dyeing through Green Synthesis with White Sugar	Rania, SA; Gomaa, EH; Emam, AG; Daher, AM	*EGYPTIAN JOURNAL OF CHEMISTRY* 10.21608/EJCHEM.2022.109662.5000	Web of Science only	Not reported in screening workbook	Not reported in screening workbook
59	2022	Eco-friendly Approach for Dyeing Synthetic Fabrics with Natural Dyes Using Electron Beam Irradiation	Elmaaty, T.A.; Abouelenin, S.; Elsisi, H.; Okubayashi, S.	*Fibers and Polymers* 10.1007/s12221-022-3144-1	Scopus only	Natural/bio-derived colorant not specified in title/abstract	nylon 6; nylon
60	2022	Eco-friendly bio-dyeing of bio-treated nylon fabric using Esfand (P. harmala) based yellow natural colorant	Adeel, S; ul Hasan, M; Batool, F; Ozomay, M; Hosseinnezhad, M; Amin, N; Hussaan, M	*JOURNAL OF ENGINEERED FIBERS AND FABRICS* 10.1177/15589250221091265	Web of Science only	Not reported in screening workbook	Not reported in screening workbook
61	2022	Ecofriendly Dyeing of Textiles with Natural Dyes Extracted from Commercial Food Processing Waste Materials	Amutha, K.; Annapoorani, S.G.; Sakthivel, P.; Sudhapriya, N.	*Journal of Natural Fibers* 10.1080/15440478.2021.1993506	Both databases	spent coffee/coffee pomace	nylon
62	2022	Herbal finishing of nylon fabric with lawsonia inermis	Das, S.; Krishnapriya, R.G.	*Man-Made Textiles in India* *Not reported in screening workbook*	Scopus only	henna/Lawsonia inermis	nylon
63	2022	Insight into Fuzzy Logic and Response Surface Methodologies for Predicting Wool and Polyamide Dyeing Behaviors with a Biological Extract of Juglans Regia	Ghanmi, H; Sebeia, N; Jabli, M; Al-Ghamdi, YO; Algohary, AM	*FIBERS AND POLYMERS* 10.1007/s12221-022-4552-y	Web of Science only	Not reported in screening workbook	Not reported in screening workbook
64	2022	Microwave-Assisted Exploration of Yellow Natural Dyes for Nylon Fabric	Rehman, F.U.; Adeel, S.; Haddar, W.; Bibi, R.; Azeem, M.; Mia, R.; Ahmed, B.	*Sustainability (Switzerland)* 10.3390/su14095599	Both databases	turmeric/curcumin; pomegranate peel/rind; saffron/Crocus sativus	nylon; polyamide
65	2022	Mordant-free dyeing of nylon fabric with mahogany (Swietenia mahagoni) seed pods: A cleaner approach of synthetic fabric coloration	Mamun, A.A.; Mahbubul Bashar, M.; Khan, S.; Roy, M.N.; Hossain, M.M.; Khan, M.A.	*Textile Research Journal* 10.1177/00405175211050526	Both databases	mahogany seed pods	nylon
66	2022	Obtaining colored patterns on polyamide fabric with laser pretreatment and pomegranate peel dyeing	Tian, Y.; Liu, Q.; Zhang, Y.; Hou, X.; Zhang, Y.	*Textile Research Journal* 10.1177/00405175221076038	Both databases	pomegranate peel/rind	polyamide
67	2022	Performances and Biocompatibility of Prepared pH-Sensitive Cotton/Polyamide 6 Medical Dressings Dyed with Halochromic Dyes	Peila, R; Varesano, A; Vineis, C; Cavalli, R; Argenziano, M; Cravello, B; Tonetti, C	*FIBERS AND POLYMERS* 10.1007/s12221-022-4990-6	Web of Science only	Not reported in screening workbook	Not reported in screening workbook
68	2022	Sustainable application of Cassia obovata–based chrysophanic acid as potential source of yellow natural colorant for textile dyeing	Hasan, M.; Adeel, S.; Batool, F.; Ahmad, T.; Tang, R.-C.; Amin, N.; Khan, S.R.	*Environmental Science and Pollution Research* 10.1007/s11356-021-16447-0	Both databases	Natural/bio-derived colorant not specified in title/abstract	nylon
69	2021	Alizarin Grafting onto Ultrasmall ZnO Nanoparticles: Mode of Binding, Stability, and Colorant Studies	De Haan, M.P.; Balakrishnan, N.; Kuzmyn, A.R.; Li, G.; Willemen, H.M.; Seide, G.; Derksen, G.C.H.; Albada, B.; Zuilhof, H.	*Langmuir* 10.1021/acs.langmuir.0c02981	Both databases	madder/alizarin/purpurin	nylon; polyamide
70	2021	Analysis of the coloring and antibacterial effects of natural dye: Pomegranate peel	Bouaziz, A.; Dridi, D.; Gargoubi, S.; Chelbi, S.; Boudokhane, C.; Kenani, A.; Aroui, S.	*Coatings* 10.3390/coatings11111277	Both databases	pomegranate peel/rind	polyamide
71	2021	Ecological Dyeing of Polyamide 6, 6(PA66) Fabrics with Monascus Pigments in Decamethylcyclopentasiloxane(D5) Solvent	Feng, L.; Ren, L.; Zhao, Y.; Dong, C.; Qiu, Y.; Zhang, H.	*Journal of Donghua University (English Edition)* 10.19884/j.1672-5220.202107011	Scopus only	Monascus pigments	polyamide 6,6 / PA66; polyamide 6 / PA6; polyamide
72	2021	Extraction and application of natural colourant from the bark of berlinia grandiflora on cotton and polyamide 6 fabrics	Asiagwu, A.K.; Otutu, J.O.; Onyesom, I.E.; Ebigwai, J.K.	*Iraqi Journal of Science* 10.24996/ijs.2021.62.6.1	Scopus only	Berlinia grandiflora	nylon 6; nylon; polyamide 6 / PA6; polyamide
73	2021	Fabric dyeing with colorimetric pH-responsive colours	Peila, R; Varesano, A; Vineis, C; Bobba, V; Bobba, J; Piriou, A; Barazzotto, M	*COLORATION TECHNOLOGY*	Web of Science only	Not reported in screening workbook	Not reported in screening workbook

				10.1111/cote.12511			
74	2021	The Influence of a Natural UV Absorber (Areca catechu) on the UV Protection and Antimicrobial Properties of Silk and Nylon Fabrics	Kabir, S.M.M.; Dhar, A.K.; Koh, J.	*Fibers and Polymers* 10.1007/s12221-021-9950-z	Scopus only	catechu/Areca catechu	nylon
75	2021	Microwave Induced Sustainable Isolation of Laccaic Acid from Lac Insect for Nylon Dyeing	Rehman, F.-U.; Adeel, S.; Pervaiz, M.; Haji, A.; Haddar, W.; Hussaan, M.; Amin, N.; Guesmi, A.	*Iranian Journal of Chemistry and Chemical Engineering* 10.30492/ijcce.2020.119496.3907	Both databases	turmeric/curcumin; lac/laccaic acid	nylon
76	2021	Study on the Dyeing Properties of Curcumin on Natural and Synthetic Fibers and Antioxidant and Antibacterial Activities	Lykidou, S.; Pashou, M.; Vouvoudi, E.; Nikolaidis, N.	*Fibers and Polymers* 10.1007/s12221-021-0412-4	Both databases	turmeric/curcumin	polyamide
77	2021	Ultrasound-assisted extraction of natural dyes from Hawthorn fruits for dyeing polyamide fabric and study its fastness, antimicrobial, and antioxidant properties	Sadeghi-Kiakhani, M.; Tehrani-Bagha, A.R.; Safapour, S.; Eshaghloo-Galugahi, S.; Etezad, S.M.	*Environment, Development and Sustainability* 10.1007/s10668-020-01017-0	Both databases	hawthorn fruits	nylon 6; nylon; polyamide
78	2020	Dyeing of textiles with natural dyes extracted from Terminalia arjuna and Thespesia populnea fruits	K, A.; S, G.A.; N, S.	*Industrial Crops and Products* 10.1016/j.indcrop.2020.112303	Scopus only	Terminalia/Thespesia	nylon
79	2020	An overview of faba bean: utilisation of husks in natural dyeing	Erdem Ismal, Ö.; Yıldırım, L.	*International Journal of Clothing Science and Technology* 10.1108/IJCST-02-2019-0014	Both databases	Natural/bio-derived colorant not specified in title/abstract	polyamide; polyamide/elastane blend
80	2020	Sustainable dyeing of selected natural and synthetic fabrics using waste teak leaves (Tectona Grandis L.)	Agrawal, A.; Chopra, S.	*Research Journal of Textile and Apparel* 10.1108/RJTA-05-2020-0046	Both databases	teak leaf	nylon
81	2020	The use of natural Areca catechu dyes for silk and nylon and its halochromic effect	Kabir, S.M.M.; Dhar, A.K.; Bhattacharjee, M.	*Journal of the Textile Institute* 10.1080/00405000.2019.1674542	Both databases	catechu/Areca catechu	nylon
82	2019	Assessment of bacterial pigments as textile colorants	Agha, Y.Y.; Bahjat, S.A.; Thanoon, M.F.	*Indian Journal of Public Health Research and Development* 10.37506/v10/i12/2019/ijphrd/192431	Scopus only	Natural/bio-derived colorant not specified in title/abstract	nylon
83	2019	Banana peel in dyeing of polyamide/elastane blend fabric	Yıldırım, L.; Erdem İşmal, Ö.	*Research Journal of Textile and Apparel* 10.1108/RJTA-07-2018-0043	Both databases	banana peel	polyamide; polyamide/elastane blend
84	2019	Colorimetric study of absorption behavior of madder natural dye on nylon using scanner	Nateri, A.S.; Hajipour, A.; Dehnavi, E.	*Indian Journal of Fibre and Textile Research* *Not reported in screening workbook*	Both databases	madder/alizarin/purpurin	nylon
85	2019	Creation of unique designs through resist dyeing with natural dyes	Yildirim, L.; Ismal, Ö.E.; Saiduyu, B.G.	*Journal of the Textile Association* *Not reported in screening workbook*	Scopus only	walnut rind/shell; pomegranate peel/rind; banana peel	polyamide; cotton/polyamide blend
86	2019	Dyeing nylon 66 fabric with riboflavin produced by engineering Bacillus subtilis	Feng, L.-L.; Dong, C.-M.; Li, Y.-P.; Zhang, H.-J.	*Textile Bioengineering and Informatics Symposium Proceedings, TBIS* *Not reported in screening workbook*	Scopus only	riboflavin	nylon 6,6 / nylon 66; nylon
87	2019	Dyeing parameters, fastness and ultraviolet protection properties of nylon dyed with mangrove bark extract	Vuthiganond, N.; Nakpathom, M.; Somboon, B.; Narumol, N.; Rungruangkitkrai, N.; Mongkholrattanasit, R.	*Materials Today: Proceedings* 10.1016/j.matpr.2019.06.254	Both databases	mangrove bark	nylon
88	2019	Energy-efficient dyeing of nylon 6 using indigo powder dyestuff after atmospheric plasma treatment at ambient pressure	Fan, F; Wu, YZ; Wu, XX	*COLORATION TECHNOLOGY*	Web of Science only	Not reported in screening workbook	Not reported in screening

				10.1111/cote.12407			workbook
89	2019	Improving the efficiency of Nesogordonia papaeverifera (Danta) as a natural dye in textile making industry	Otutu, J.O.; Asiagwu, A.K.; Onyesom, I.E.; Ebigwai, J.K.; Itoya, P.O.	*Bulletin of the Chemical Society of Ethiopia* 10.4314/bcse.v33i3.3	Both databases	Nesogordonia papaeverifera	nylon 6,6 / nylon 66; nylon 6; nylon
90	2019	Inkjet Printing of Curcumin-Based Ink for Coloration and Bioactivation of Polyamide, Silk, and Wool Fabrics	Zhou, YY; Yu, JC; Biswas, TT; Tang, RC; Nierstrasz, V	*ACS SUSTAINABLE CHEMISTRY & ENGINEERING* 10.1021/acssuschemeng.8b04650	Web of Science only	Not reported in screening workbook	Not reported in screening workbook
91	2019	Simple crystallization approach for enhancing function of plant-based madder dye and performance of dyed fabric	Du, H.; Wang, Q.; Tian, M.; Yang, X.	*Heliyon* 10.1016/j.heliyon.2019.e02232	Both databases	madder/alizarin/purpurin	nylon
92	2019	Studies on the dyeing and functional properties of modified berberine for a variety of fabrics	Liu, Y.; Cao, J.; Niu, W.; Liu, M.; Guo, X.	*AATCC Journal of Research* 10.14504/ajr.6.4.2	Both databases	berberine	polyamide
93	2019	A Study of Behavior of Sesame Dyeing on Nylon	Zhou, CE; Zhang, Q; Kan, CW	*AATCC JOURNAL OF RESEARCH* 10.14504/ajr.6.1.4	Web of Science only	Not reported in screening workbook	Not reported in screening workbook
94	2018	Application of Prunus amygdalus By-products in Eco-friendly Dyeing of Textile Fabrics	Moussa, I.; Baaka, N.; Khiari, R.; Moussa, A.; Mortha, G.; Mhenni, M.F.	*Journal of Renewable Materials* 10.7569/JRM.2017.634141	Both databases	lignin nanoparticles	polyamide
95	2018	Dyeing of Polyester and Nylon with Semi-synthetic Azo Dye by Chemical Modification of Natural Source Areca Nut	Pawar, AB; More, SP; Adivarekar, RV	*NATURAL PRODUCTS AND BIOPROSPECTING* 10.1007/s13659-017-0144-8	Web of Science only	Not reported in screening workbook	Not reported in screening workbook
96	2018	Silkworm excrement used for dyeing textiles	Vila, NT; Silva, MG; Musialak, AL; Fernandes, M; Souto, AP; Ferreira, AJS	*18TH WORLD TEXTILE CONFERENCE (AUTEX 2018)* 10.1088/1757-899X/460/1/012028	Web of Science only	Not reported in screening workbook	Not reported in screening workbook
97	2018	Surface activation of textile fibers by plasma DBD for dyeing with teak leaf	Ferreira, A.J.S.; Goede, M.B.; Vila, N.T.; Silva, M.G.; Fiori, S.; Nascimento, T.A.; Fernandes, M.; Souto, A.P.	*IOP Conference Series: Materials Science and Engineering* 10.1088/1757-899X/460/1/012030	Both databases	teak leaf	polyamide 6,6 / PA66; polyamide 6 / PA6; polyamide
98	2018	Thermodynamic and kinetic studies of the adsorption behaviour of the natural dye cochineal on polyamide 66	Sadeghi-Kiakhani, M.; Safapour, S.; Mirnezhad, S.	*Coloration Technology* 10.1111/cote.12342	Both databases	cochineal/carmine	polyamide 6,6 / PA66; polyamide
99	2017	Chemical analysis of the characteristics of Tunisian Juglans regia L. fractions: Antibacterial potential, gas chromatography-mass spectroscopy and a full investigation of their dyeing properties	Jabli, M; Sebeia, N; Boulares, M; Faidi, W	*INDUSTRIAL CROPS AND PRODUCTS* 10.1016/j.indcrop.2017.07.032	Web of Science only	Not reported in screening workbook	Not reported in screening workbook
100	2017	Optimisation of the recovery of carotenoids from tomato processing wastes: application on textile dyeing and assessment of its antioxidant activity	Baaka, N.; El Ksibi, I.; Mhenni, M.F.	*Natural Product Research* 10.1080/14786419.2016.1226828	Both databases	carotenoids/tomato waste	polyamide
101	2017	Reversible and Washing Resistant Textile-Based Optical pH Sensors by Dyeing Fabrics with Curcuma	Giachet, FT; Vineis, C; Ramirez, DOS; Carletto, RA; Varesano, A; Mazzuchetti, G	*FIBERS AND POLYMERS* 10.1007/s12221-017-6757-z	Web of Science only	Not reported in screening workbook	Not reported in screening workbook
102	2017	Waste from eucalyptus wood steaming as a natural dye source for textile fibers	Rossi, T.; Silva, P.M.S.; De Moura, L.F.; Araújo, M.C.; Brito, J.O.; Freeman, H.S.	*Journal of Cleaner Production* 10.1016/j.jclepro.2016.12.109	Both databases	eucalyptus	nylon
103	2017	Wood-rotting fungal pigments as colorant coatings on oil-based textile dyes	Agurto, M.E.P.; Gutierrez, S.M.V.; Chen, H.-L.; Robinson, S.C.	*Coatings* 10.3390/coatings7100152	Scopus only	Natural/bio-derived colorant not specified in title/abstract	nylon

104	2016	Dyeing of enzymatically modified polyamide fabrics with natural dyes	Bahtiyari, M.I.; Benli, H.	*Industria Textila* *Not reported in screening workbook*	Both databases	Natural/bio-derived colorant not specified in title/abstract	polyamide
105	2016	Dyeing of nylon fabric with natural dye from cassia fistula fruit: A research on effect metal mordants concentration	Mongkholrattanasit, R.; Klaichoi, C.; Rungruangkitkrai, N.; Sasivatchutikool, P.	*Materials Science Forum* 10.4028/www.scientific.net/MSF.857.487	Scopus only	cassia fistula	nylon
106	2016	Dyeing of polyamide fibre with cochineal natural dye	Shams Nateri, A.; Dehnavi, E.; Hajipour, A.; Ekrami, E.	*Pigment and Resin Technology* 10.1108/PRT-05-2015-0043	Both databases	cochineal/carmine	polyamide
107	2016	Dyeing properties of nylon fabrics with mangrove bark extract via mordanting methods	Punrattanasin, N.; Nakpathom, M.; Somboon, B.; Narumol, N.; Rungruangkitkrai, N.; Mongkholrattanasit, R.	*Materials Science Forum* 10.4028/www.scientific.net/MSF.857.495	Scopus only	mangrove bark	nylon
108	2016	Eco-friendly dyeings of textiles with extract from pomegranate arils with seeds spectroscopic, colorimetric and statistical assessment	Popescu, V.; Pui, A.; Sandu, I.C.A.; Popescu, G.	*Revista de Chimie* *Not reported in screening workbook*	Both databases	pomegranate peel/rind	polyamide
109	2016	Effect of ozonation process on dyeing of polyamide fabrics with a natural dye: Walnut rind	Atav, R.; Namirti, O.	*Industria Textila* *Not reported in screening workbook*	Scopus only	walnut rind/shell	polyamide
110	2016	Extraction of polyphenolic dyes from henna, pomegranate rind, and Pterocarya fraxinifolia for nylon 6 dyeing	Ebrahimi, I.; Parvinzadeh Gashti, M.	*Coloration Technology* 10.1111/cote.12204	Both databases	henna/Lawsonia inermis; pomegranate peel/rind	nylon 6; nylon
111	2016	Investigation of ecofriendly ultrasonic coloring with natural dyes	Coman, D.; Vrinceanu, N.; Oancea, S.	*Academic Journal of Manufacturing Engineering* *Not reported in screening workbook*	Scopus only	walnut rind/shell	polyamide
112	2016	Study of textile materials behavior at dyeing with colored aqueous extract obtained by boiling peels, arils and seeds of Punica Granatum Spectroscopic, colorimetric and statistic evaluations	Popescu, V.; Sandu, I.C.A.; Popescu, G.	*Revista de Chimie* *Not reported in screening workbook*	Both databases	pomegranate peel/rind	polyamide
113	2015	Eco-friendly dyeing of wool and nylon using madder as a natural dye: Kinetic and adsorption isotherm studies	Sadeghi-Kiakhani, M.	*International Journal of Environmental Science and Technology* 10.1007/s13762-015-0770-9	Both databases	madder/alizarin/purpurin	nylon
114	2015	Use of ultrasonic energy for enhancing the dyeing performances of polyamide fibers with olive vegetable water	Haddar, W.; Baaka, N.; Meksi, N.; Ticha, M.B.; Guesmi, A.; Mhenni, M.F.	*Fibers and Polymers* 10.1007/s12221-015-4931-8	Both databases	olive leaf/vegetable water	polyamide
115	2014	Colorimetric Study on Polyamides Dyeing With Weld and Pomegranate Peel Natural Dyes	Shams-Nateri, A.; Hajipour, A.; Dehnavi, E.; Ekrami, E.	*Clothing and Textiles Research Journal* 10.1177/0887302X14525658	Both databases	pomegranate peel/rind; weld/Reseda luteola	nylon
116	2014	Crude bacterial extracts of two new Streptomyces sp. isolates as bio-colorants for textile dyeing	Kramar, A.; Ilic-Tomic, T.; Petkovic, M.; Radulović, N.; Kostic, M.; Jocic, D.; Nikodinovic-Runic, J.	*World Journal of Microbiology and Biotechnology* 10.1007/s11274-014-1644-x	Both databases	prodigiosin	polyamide
117	2014	Natural dyeing and antibacterial activity of atmospheric-plasma-treated nylon 6 fabric	Haji, A.; Mousavi Shoushtari, A.; Mirafshar, M.	*Coloration Technology* 10.1111/cote.12060	Both databases	berberine	nylon 6; nylon
118	2013	Adsorption properties of lac dyes on wool, silk, and nylon	Wei, B.; Chen, Q.-Y.; Chen, G.; Tang, R.-C.; Zhang, J.	*Journal of Chemistry* 10.1155/2013/546839	Scopus only	lac/laccaic acid	nylon
119	2013	Antibacterial dyeing of polyamide using turmeric as a natural dye	Mirjalili, M.; Karimi, L.	*Autex Research Journal* 10.2478/v10304-012-0023-7	Both databases	turmeric/curcumin	nylon 6,6 / nylon 66; nylon 6; nylon; polyamide

120	2013	Dyeing and functional finishing of silk, nylon 6 and polyester fabrics with baicalin	Cao, J.; Li, X.	*Advanced Materials Research* 10.4028/www.scientific.net/AMR.627.184	Both databases	baicalin	nylon 6; nylon
121	2013	Extraction and characterization of natural dye from green walnut shells and its use in dyeing polyamide: Focus on antibacterial properties	Mirjalili, M.; Karimi, L.	*Journal of Chemistry* 10.1155/2013/375352	Both databases	walnut rind/shell	polyamide
122	2013	Mutant breeding of serratia marcescens strain for enhancing prodigiosin production and application to textiles	Liu, X.; Wang, Y.; Sun, S.; Zhu, C.; Xu, W.; Park, Y.; Zhou, H.	*Preparative Biochemistry and Biotechnology* 10.1080/10826068.2012.721850	Both databases	prodigiosin	polyamide
123	2013	Natural dye extraction from waste barks of Turkish red pine (Pinus brutia Ten.) timber and eco-friendly natural dyeing of various textile fibers	Avinc, O.; Celik, A.; Gedik, G.; Yavas, A.	*Fibers and Polymers* 10.1007/s12221-013-0866-0	Scopus only	Natural/bio-derived colorant not specified in title/abstract	polyamide
124	2013	Study on dyeing properties of turmeric yellow on nylon fiber	Yin, M.Y.; Wang, X.R.	*Advanced Materials Research* 10.4028/www.scientific.net/AMR.821-822.577	Both databases	turmeric/curcumin	nylon
125	2012	Chromatographic and colorimetric characterizations of brominated indigoid dyeings	Koren, Z.C.	*Dyes and Pigments* 10.1016/j.dyepig.2012.06.016	Scopus only	Natural/bio-derived colorant not specified in title/abstract	nylon
126	2012	Dyeing parameters of hydroxynaphthoquinones extracted from Arnebia nobilis Rech.f	Arora, A.; Rastogi, D.; Gupta, D.; Gulrajani, M.L.	*Indian Journal of Fibre and Textile Research* *Not reported in screening workbook*	Both databases	Arnebia nobilis/Ratanjot	nylon
127	2012	Mathematical simulation of cochineal dyeing process of wool and nylon	Wei, B.; Tang, R.-C.; Chen, G.	*Advanced Science Letters* 10.1166/asl.2012.3375	Scopus only	cochineal/carmine	nylon
128	2011	Dyeing nylon using alder (Ainus glutnisa) forest tree wood disposals as natural dye	Mohammadzade, H.; Ekrami, E.	*World Applied Sciences Journal* *Not reported in screening workbook*	Scopus only	alder wood	nylon
129	2011	Dyeing of silk and nylon 6 fibers with baicalin	Li, X.; Cao, J.	*Advanced Materials Research* 10.4028/www.scientific.net/AMR.331.287	Both databases	baicalin	nylon 6; nylon
130	2011	Extraction, characterization and application of natural dyes from water opepe (Nuclea diderricchi) and water ekimi (Vanilya planofolia) on natural and synthetic fibres	Otutu, J.O.; Yakubu, M.K.	*Biosciences Biotechnology Research Asia* 10.13005/bbra/886	Scopus only	Natural/bio-derived colorant not specified in title/abstract	nylon
131	2011	Modifying the dyeability of nylon for natural dye lac	Pant, S.; Mangal, R.	*Man-Made Textiles in India* *Not reported in screening workbook*	Scopus only	lac/laccaic acid	nylon
132	2010	Creating multicoloured effect with natural dyes	Pant, S.; Bhalla, S.	*Colourage* *Not reported in screening workbook*	Scopus only	lac/laccaic acid; catechu/Areca catechu	nylon
133	2010	Dyeing of Nylon with natural colourants	Teli, M.D.; Shah, R.; Sabale, A.G.	*Colourage* *Not reported in screening workbook*	Scopus only	turmeric/curcumin; madder/alizarin/purpurin; henna/Lawsonia inermis; catechu/Areca catechu; pomegranate peel/rind; rhubarb/Rheum	nylon
134	2010	Dyeing of polyamide fabrics with a natural dye: White onion skin	Atav, R.; Namirti, O.	*Fiber Society Spring 2010 International Conference* *Not reported in screening workbook*	Scopus only	Natural/bio-derived colorant not specified in title/abstract	polyamide
135	2010	Extraction of natural dyes for textile dyeing from the by-	Otutu, J.O.; Osabohien, E.;	*Biosciences Biotechnology*	Scopus only	Natural/bio-derived	nylon 6,6 / nylon

		products of the timber industry	Efurhievwe, E.M.	*Research Asia*<br>*Not reported in screening workbook*		colorant not specified in title/abstract	66; nylon
136	2009	Application of natural dyes on modified nylon, nylon and silk - 'Catechu'	Adivarekar, R.V.; Kanoongo, N.V.; Manjrekar, S.G.; Teli, M.D.	*Journal of the Textile Association*<br>*Not reported in screening workbook*	Scopus only	catechu/Areca catechu	nylon; polyamide
137	2009	Dyeing of Nylon 6 fibres with Calendula officinalis & Casuarina cunninghamiana extracts	Mohamed, R.M.	*Colourage*<br>*Not reported in screening workbook*	Scopus only	calendula; casuarina	nylon 6; nylon; polyamide
138	2009	Dyeing of nylon with Rheum emodi	Maulik, S.R.; Roy, S.; Banerjee, A.	*Asian Dyer*<br>*Not reported in screening workbook*	Scopus only	rhubarb/Rheum	nylon
139	2009	Eco safe natural dyes & application on textile materials	Tarafder, N.	*Man-Made Textiles in India*<br>*Not reported in screening workbook*	Scopus only	Natural/bio-derived colorant not specified in title/abstract	nylon
140	2007	Berberine finishing for developing antimicrobial nylon 66 fibers: % Exhaustion, colorimetric analysis, antimicrobial study, and empirical modeling	Son, Y.-A.; Kim, B.-S.; Ravikumar, K.; Kim, T.-K.	*Journal of Applied Polymer Science*<br>10.1002/app.25364	Both databases	berberine	nylon 6,6 / nylon 66; nylon
141	2007	UPF characteristics of natural dyes and textiles dyed with them	Gupta, D.	*Colourage*<br>*Not reported in screening workbook*	Scopus only	Terminalia/Thespesia	nylon
142	2006	Solvent extraction of yellow colouring matter from natural source-flowers and its application on hydrophobic substrates	Sarkar, D.; Mazumdar, K.; Datta, S.	*Man-Made Textiles in India*<br>*Not reported in screening workbook*	Scopus only	Extract-based colorant/finish	nylon
143	2006	Ultrasonic assisted dyeing v. dyeing of nylon with lac as a natural dye	Mashaly, H.M.	*Man-Made Textiles in India*<br>*Not reported in screening workbook*	Scopus only	lac/laccaic acid	nylon
144	2004	Effect of mordants on polyamide fabric dyed with some eco-friendly natural textile colourants	Bhattacharya, S.K.; Chatterjee, S.M.; Dutta, C.	*Man-Made Textiles in India*<br>*Not reported in screening workbook*	Scopus only	pomegranate peel/rind	polyamide
145	2003	Dyeing of red sandalwood on wool and nylon	Gulrajani, M.L.; Bhaumik, S.; Oppermann, W.; Hardtmann, G.	*Indian Journal of Fibre and Textile Research*<br>*Not reported in screening workbook*	Scopus only	red sandalwood	nylon
146	2002	Dyeing of nylon with babool bark extract	Patel, B.H.; Agarwal, B.J.; Patel, H.M.	*Textile Asia*<br>*Not reported in screening workbook*	Scopus only	babool/Acacia nilotica	nylon
147	2002	Eco-friendly dyeing with Tulsi leave extract	Patel, K.J.; Patel, B.H.; Naik, J.A.; Bhavsar, A.M.	*Man-Made Textiles in India*<br>*Not reported in screening workbook*	Scopus only	Natural/bio-derived colorant not specified in title/abstract	nylon
148	2002	Evaluation of fastness characteristics and colour value of selected natural dyes on synthetic fibres	Gulrajani, M.L.; Maulik, S.R.	*Textile Trends*<br>*Not reported in screening workbook*	Scopus only	annatto/norbixin/bixin; berberine; Arnebia nobilis/Ratanjot	nylon
149	2002	Kinetic and thermodynamic studies on red sandalwood	Gulrajani, ML; Bhaumik, S; Oppermann, W; Hardtmann, G	*INDIAN JOURNAL OF FIBRE & TEXTILE RESEARCH*<br>*Not reported in screening workbook*	Web of Science only	Not reported in screening workbook	Not reported in screening workbook
150	2002	Some studies on natural dyed polyamide fibres	Bhattacharya, S.K.; Chatterjee,	*Journal of the Institution of*	Scopus only	Natural/bio-derived	nylon; polyamide

			S.M.	*Engineers (India), Part TX: Textile Engineering Division* *Not reported in screening workbook*		colorant not specified in title/abstract	
151	2001	Dyeing studies with hydroxyanthraquinones extracted from Indian madder. Part 1: Dyeing of nylon with purpurin	Gupta, D.; Kumari, S.; Gulrajani, M.	*Coloration Technology* 10.1111/j.1478-4408.2001.tb00084.x	Both databases	madder/alizarin/purpurin	nylon
152	2001	Dyeing studies with hydroxyanthraquinones extracted from Indian madder. Part 2: Dyeing of nylon and polyester with nordamncanthal	Gupta, D.; Kumari, S.; Gulrajani, M.	*Coloration Technology* 10.1111/j.1478-4408.2001.tb00085.x	Scopus only	madder/alizarin/purpurin	nylon
153	2001	Emodin and dermocybin natural anthraquinones as high-temperature disperse dyes for polyester and polyamide	Räisänen, R.; Nousiainen, P.; Hynninen, P.H.	*Textile Research Journal* 10.1177/004051750107101012	Both databases	emodin/dermocybin	polyamide
154	2001	Emodin and Dermocybin Natural Anthraquinones as Mordant Dyes for Wool and Polyamide	Räisänen, R.; Nousiainen, P.; Hynninen, P.H.	*Textile Research Journal* 10.1177/004051750107101113	Both databases	emodin/dermocybin	polyamide
155	2000	Dyeing of nylon with a natural colourant: Babool	Patel B.H., Agarwal B.J.	*Man-Made Textiles in India* *Not reported in screening workbook*	Scopus only	babool/Acacia nilotica	nylon
156	2000	Extraction, characterisation and application of henna (lele) on nylon 6,6 fabrics	Yakubu, M.K.; Baba, A.M.	*Man-Made Textiles in India* *Not reported in screening workbook*	Scopus only	henna/Lawsonia inermis	nylon 6,6 / nylon 66; nylon 6; nylon
157	2000	Isolation of bacteria producing bluish-purple pigment and use for dyeing	Shirata, A.; Tsukamoto, T.; Yasui, H.; Hata, T.; Hayasaka, S.; Kojima, A.; Kato, H.	*Japan Agricultural Research Quarterly* *Not reported in screening workbook*	Both databases	Natural/bio-derived colorant not specified in title/abstract	nylon
158	2000	Mechanism of dyeing synthetic fibres with natural dyes	Gupta, D.	*Colourage* *Not reported in screening workbook*	Scopus only	carotenoids/tomato waste	nylon
